# Supplementary figures and images for: CDK1 controls CHMP7-dependent nuclear envelope reformation (part 1 of 2)
Source: eLife. 2021 Jul 21;10:e59999. doi: 10.7554/eLife.59999 (PMC8324300; doi:10.7554/eLife.59999)

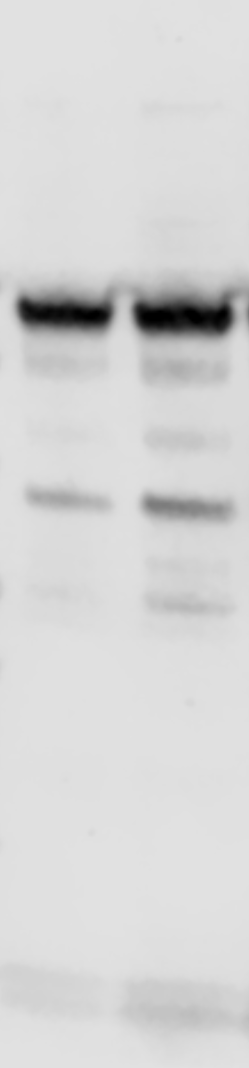

Supplement: Source data 1. [file elife-59999-data1.zip › Raw Cropped blots copy/Figure4_S1A_CHMP7alone_CHMP7_VC]

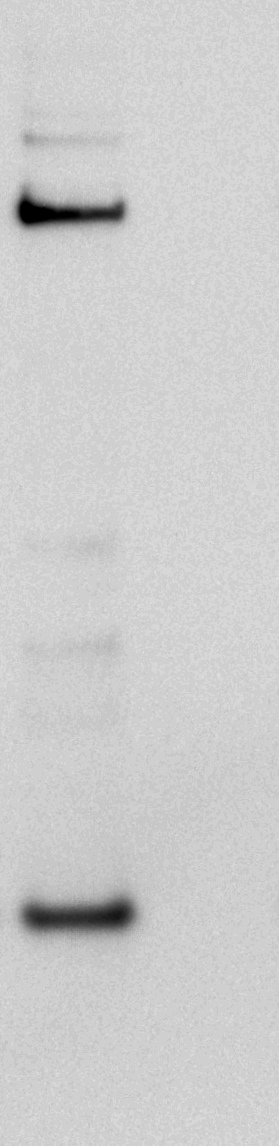

Supplement: Source data 1. [file elife-59999-data1.zip › Raw Cropped blots copy/Figure4_S1C_HALEM2_C7DD_HA_VC.tif]

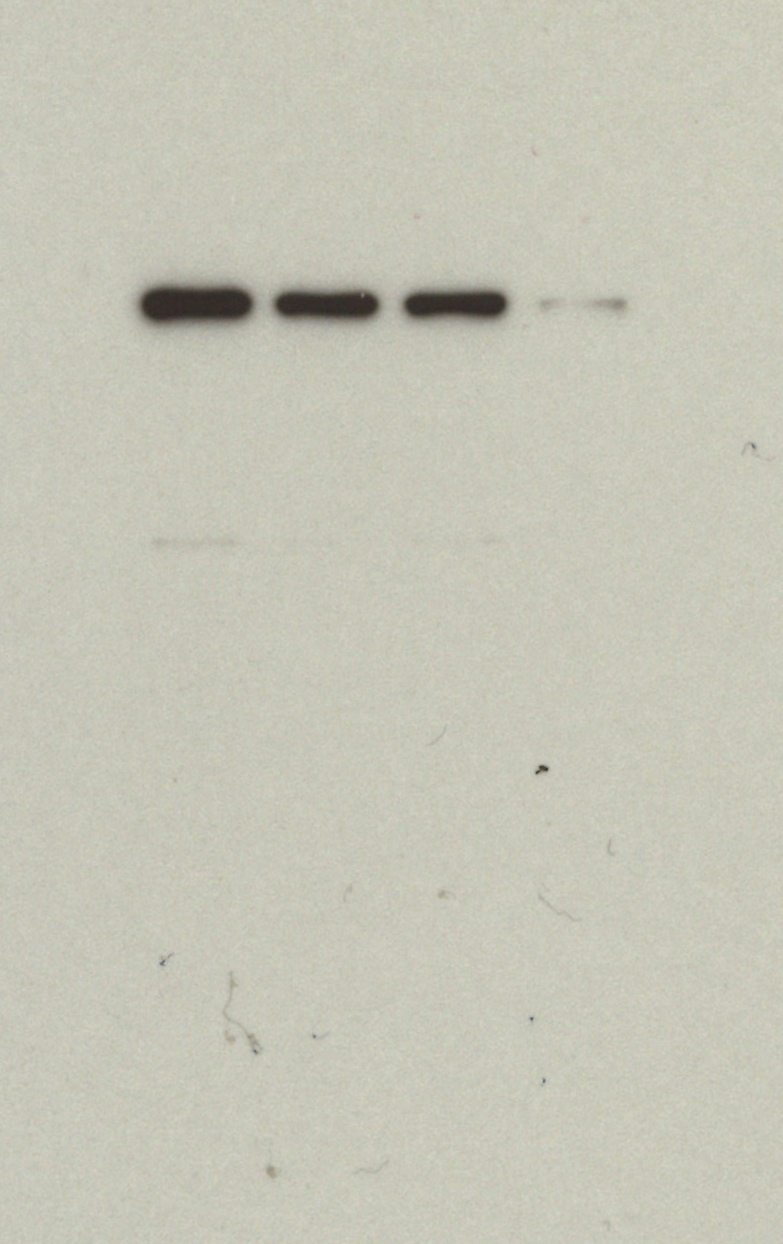

Supplement: Source data 1. [file elife-59999-data1.zip › Raw Cropped blots copy/FIgure5E_KH-pS-P_Pulldown_VC.tif]

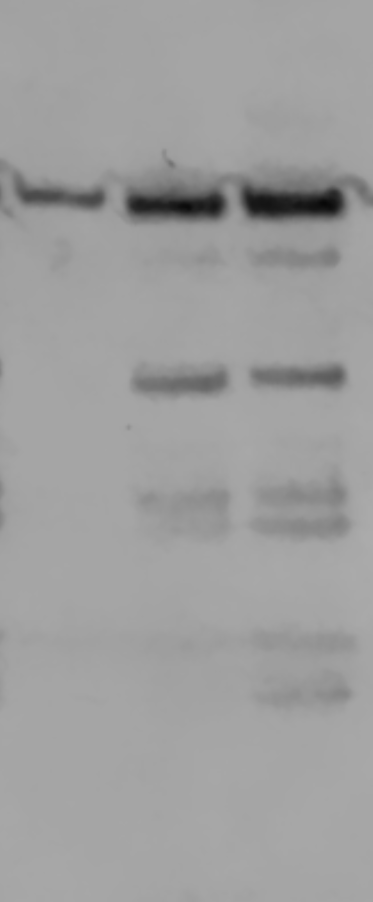

Supplement: Source data 1. [file elife-59999-data1.zip › Raw Cropped blots copy/Figure4G_input_CHMP7_VC]

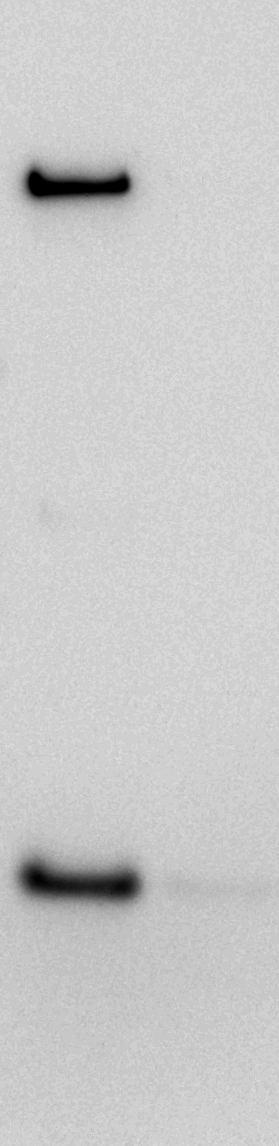

Supplement: Source data 1. [file elife-59999-data1.zip › Raw Cropped blots copy/Figure4_S1C_HALEM2alone_HA_VC]

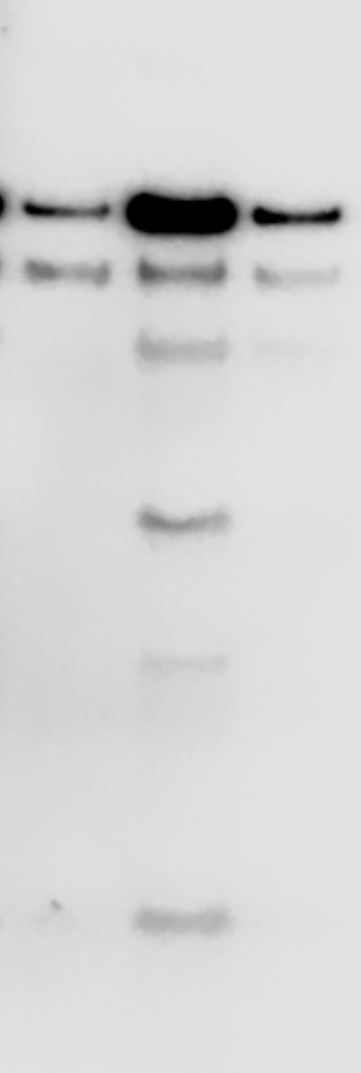

Supplement: Source data 1. [file elife-59999-data1.zip › Raw Cropped blots copy/Figure4G_inputpulldown_darkerexposure_HA_VC.tif]

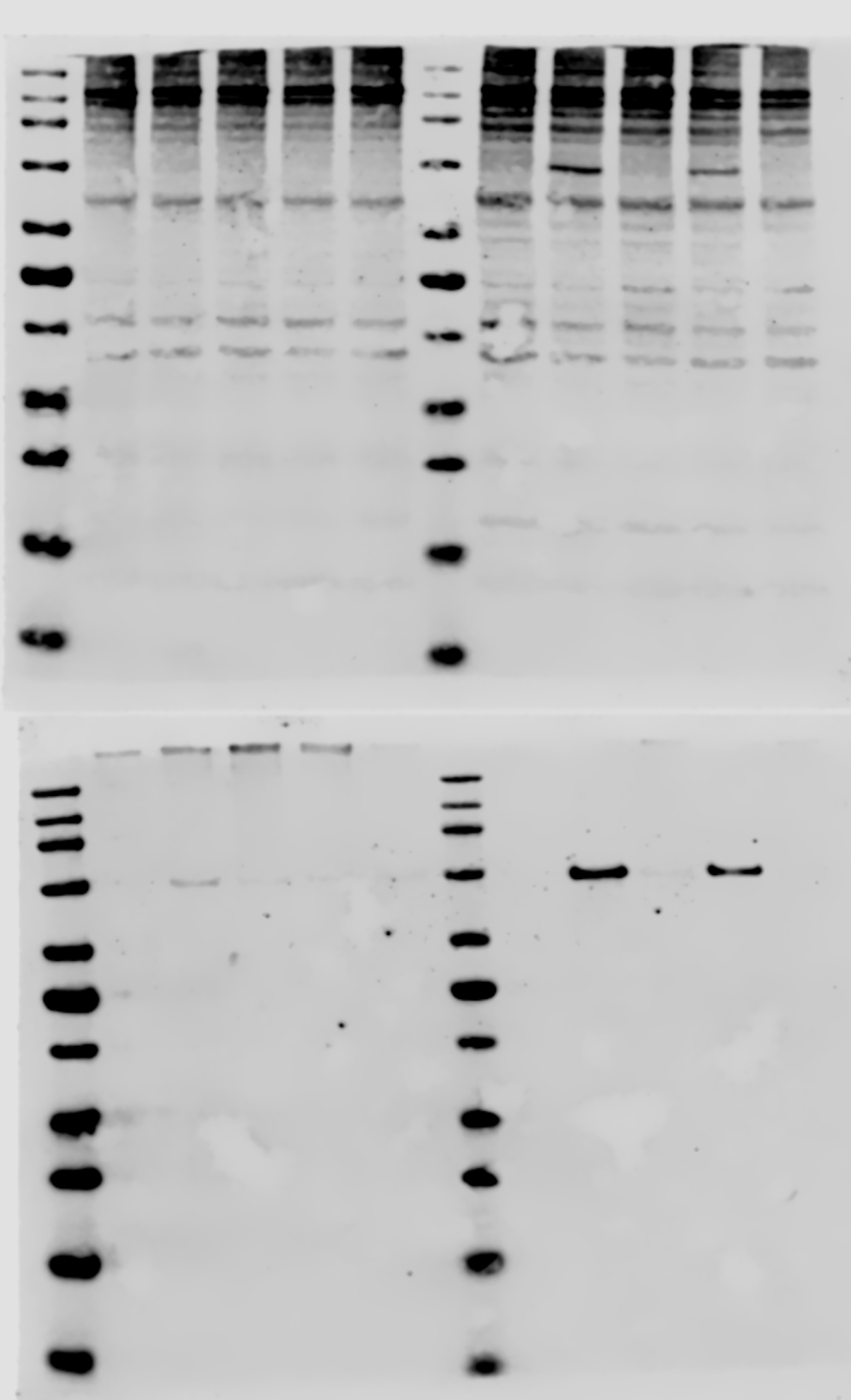

Supplement: Source data 1. [file elife-59999-data1.zip › Raw Cropped blots copy/Figure5_S2D_3892_IPtop_GTrapbottom_VC.tif]

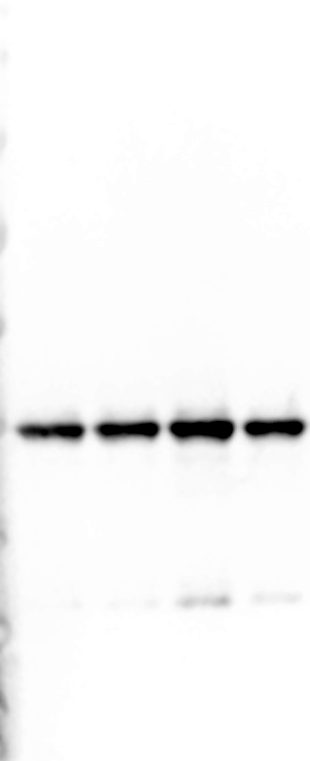

Supplement: Source data 1. [file elife-59999-data1.zip › Raw Cropped blots copy/Figure2_S2B_Input_VPS4MIT_GFP_VC]

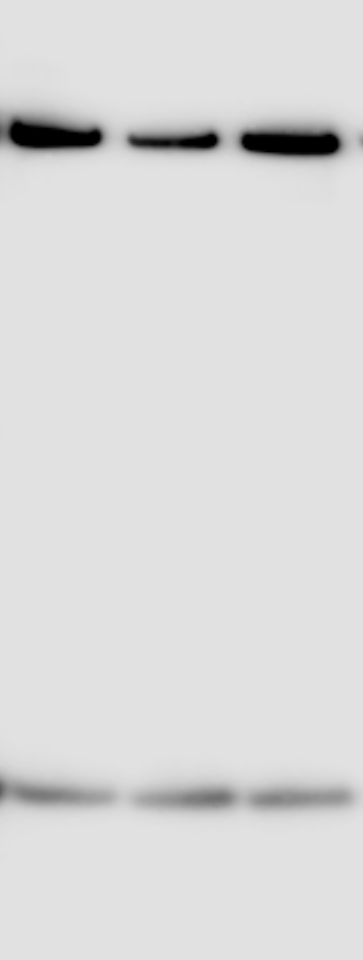

Supplement: Source data 1. [file elife-59999-data1.zip › Raw Cropped blots copy/Figure4G_inputpulldown_HA_VC.tif]

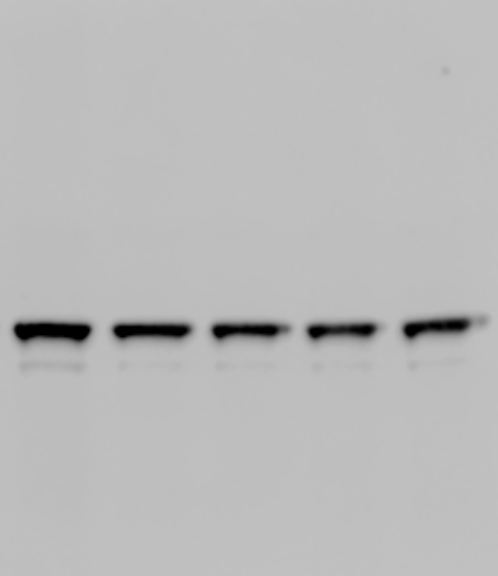

Supplement: Source data 1. [file elife-59999-data1.zip › Raw Cropped blots copy/Figure3_S1D_GAPDH_VC.tif]

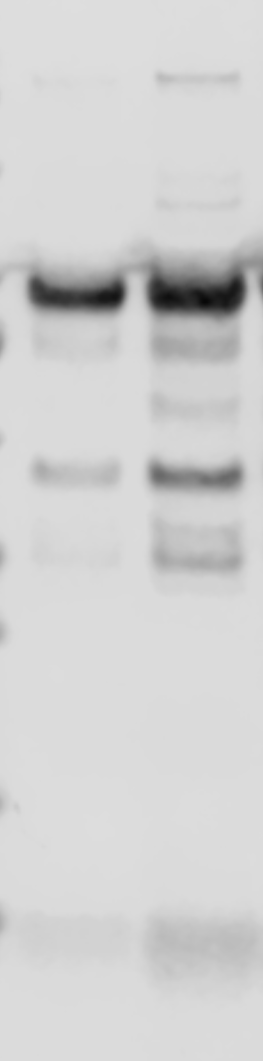

Supplement: Source data 1. [file elife-59999-data1.zip › Raw Cropped blots copy/Figure4C_CHMP7__WT_VC]

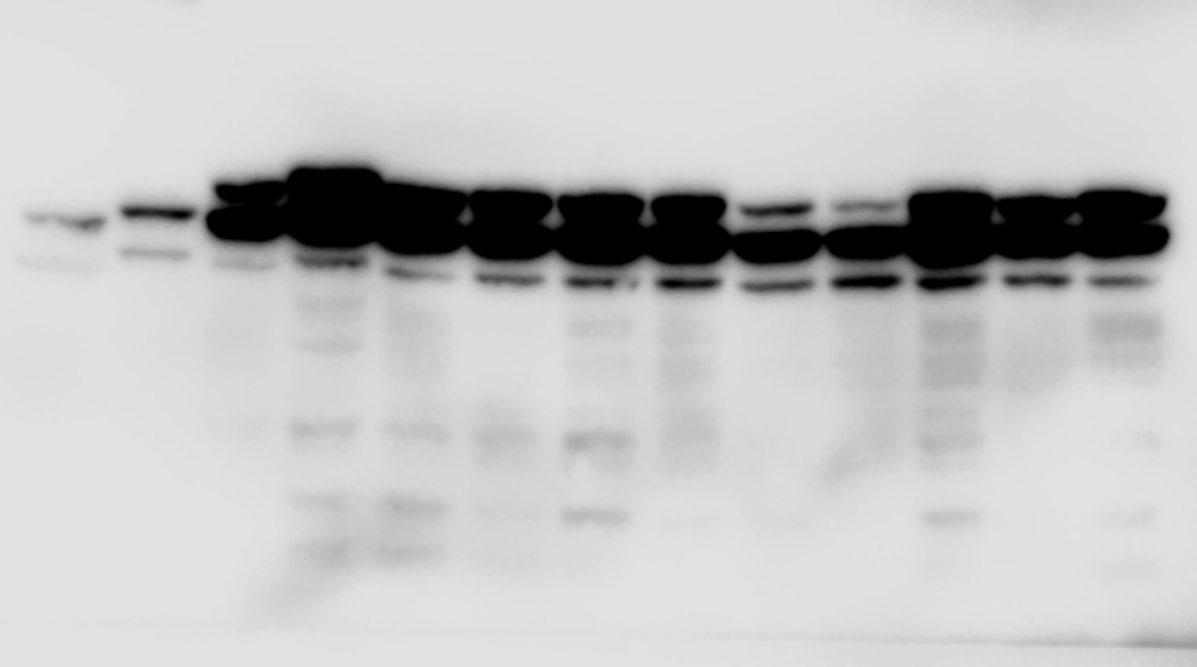

Supplement: Source data 1. [file elife-59999-data1.zip › Raw Cropped blots copy/Figure3_S1C_PhostagM27M59_GFP_darker_VC.tif]

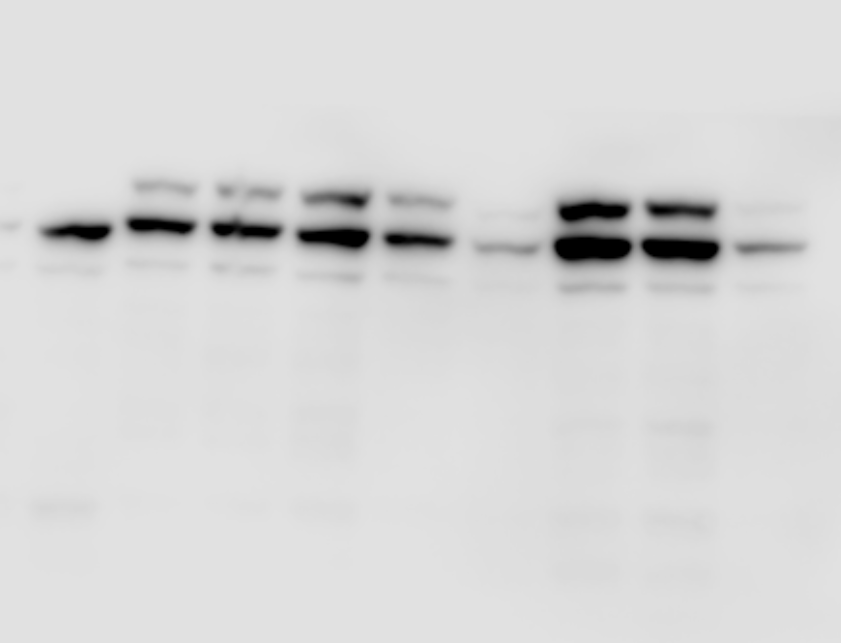

Supplement: Source data 1. [file elife-59999-data1.zip › Raw Cropped blots copy/Figure3_S1C_PhostagM1M23_GFP_VC.tif]

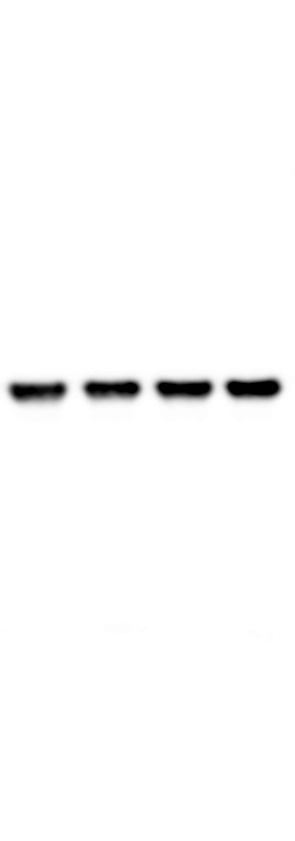

Supplement: Source data 1. [file elife-59999-data1.zip › Raw Cropped blots copy/Figure3_S1B_GAPDH_VC.tif]

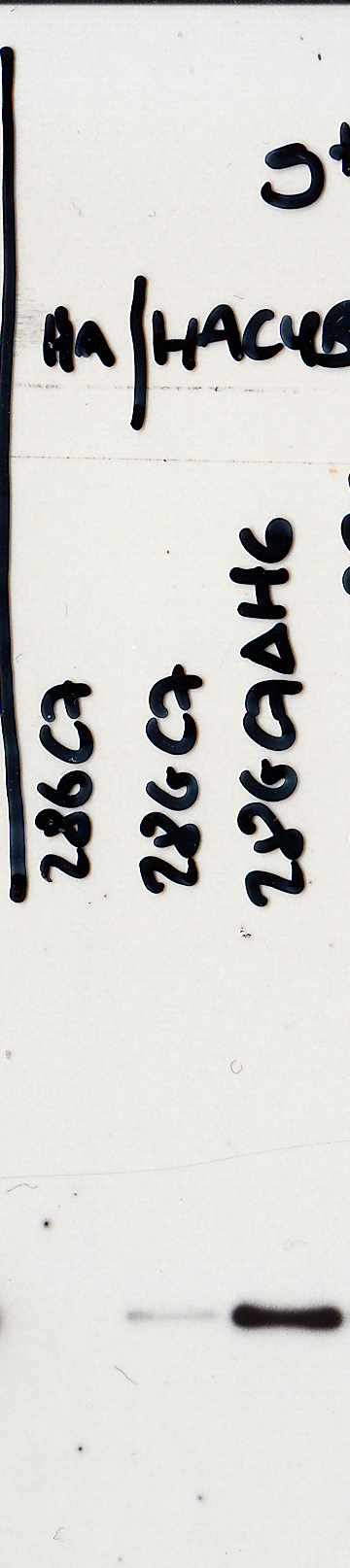

Supplement: Source data 1. [file elife-59999-data1.zip › Raw Cropped blots copy/Figure2_S3C_pulldown_HA_VC.tif]

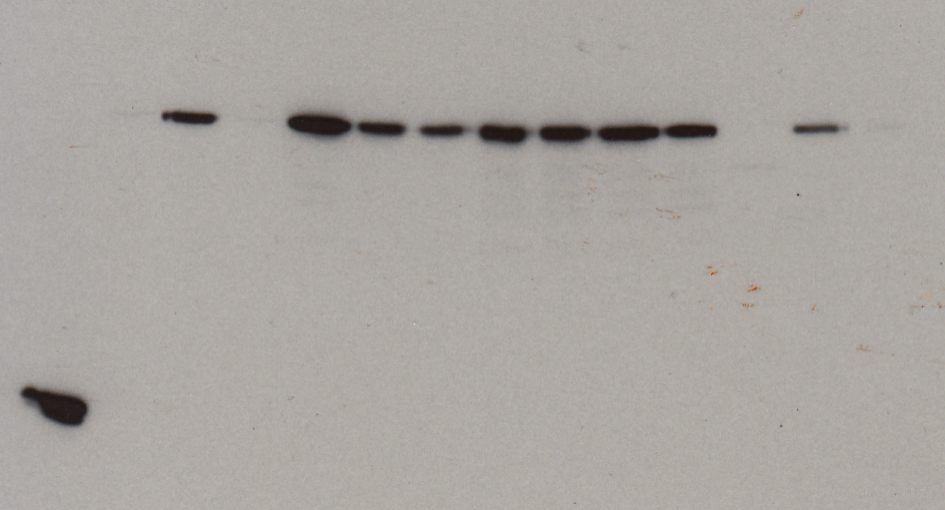

Supplement: Source data 1. [file elife-59999-data1.zip › Raw Cropped blots copy/Figure3_S1A_HK-pS-P_PD_VC.tif]

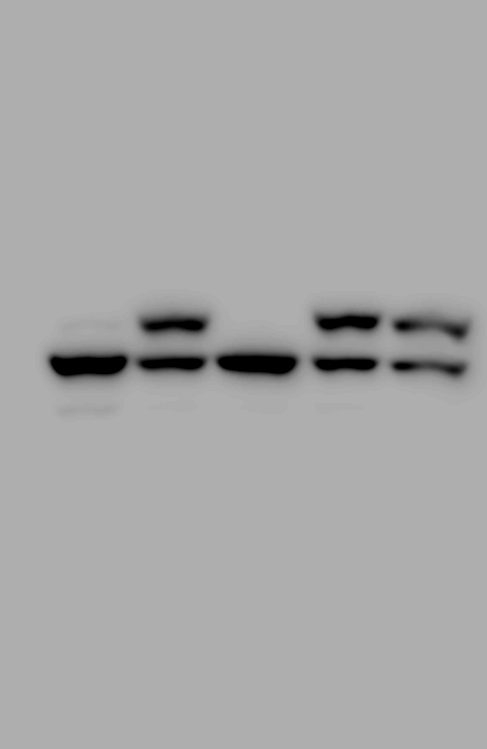

Supplement: Source data 1. [file elife-59999-data1.zip › Raw Cropped blots copy/Figure3_S1D_Phostag_GFP_VC.tif]

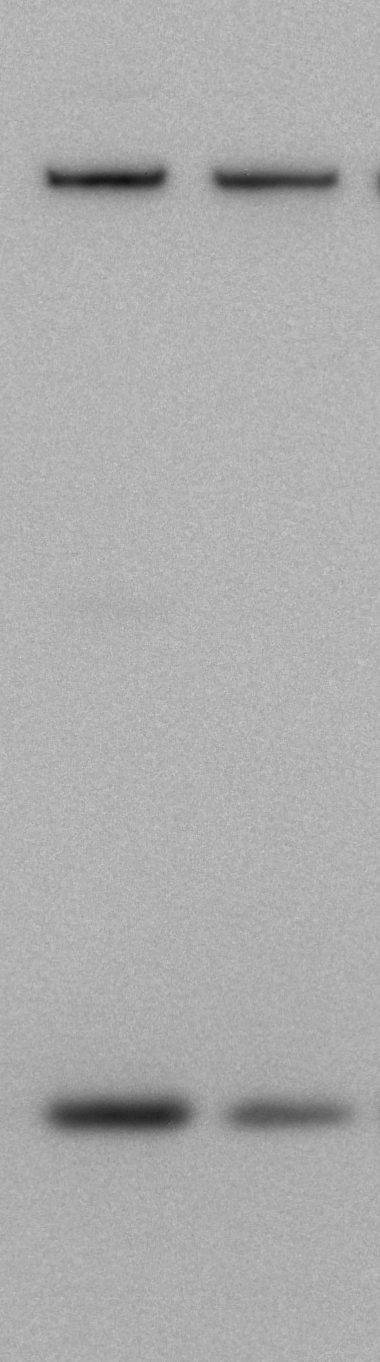

Supplement: Source data 1. [file elife-59999-data1.zip › Raw Cropped blots copy/Figure4_S1A_LEM2withC7_HA_VC]

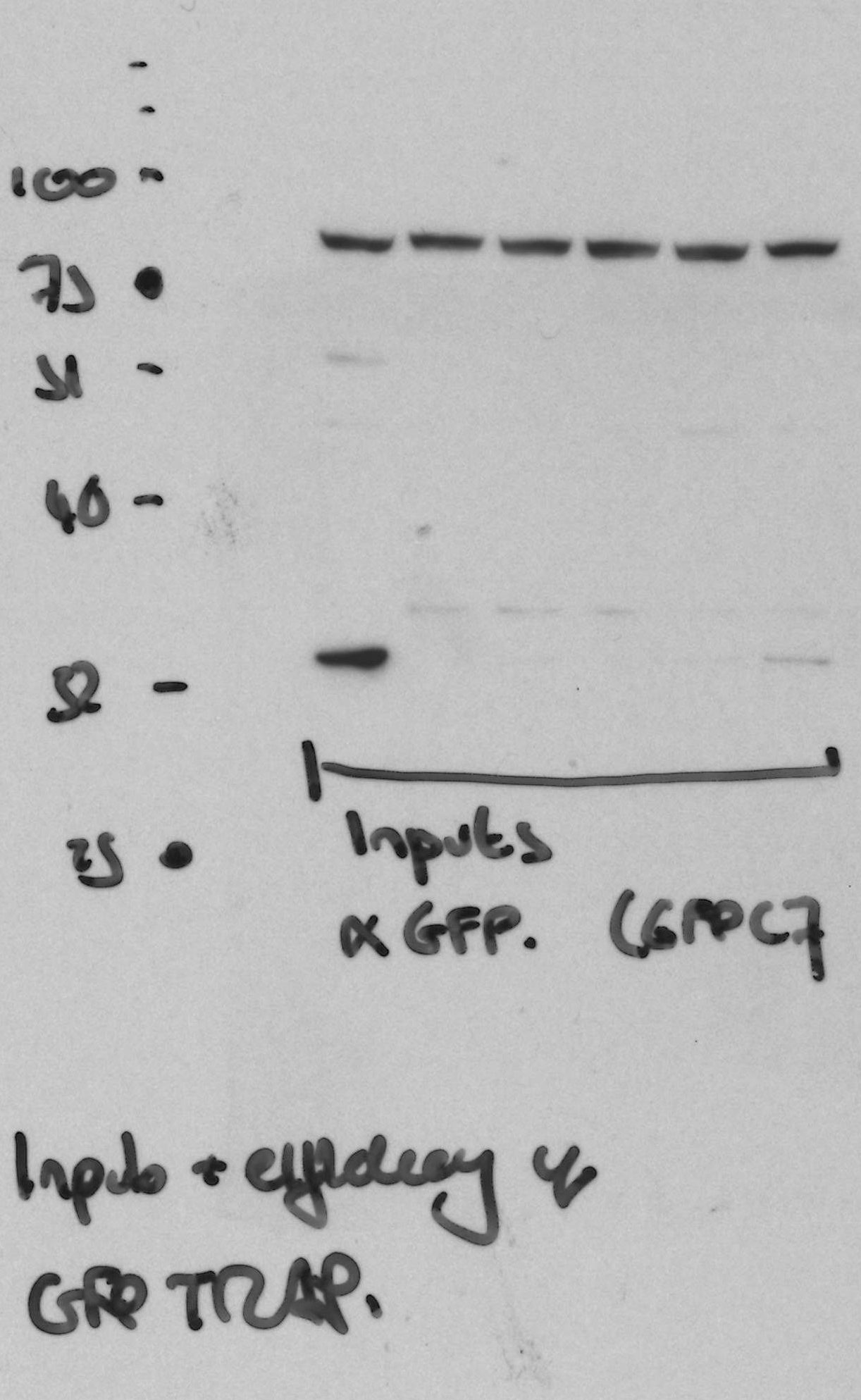

Supplement: Source data 1. [file elife-59999-data1.zip › Raw Cropped blots copy/Figure5E_GFP_innputs_VC.tif]

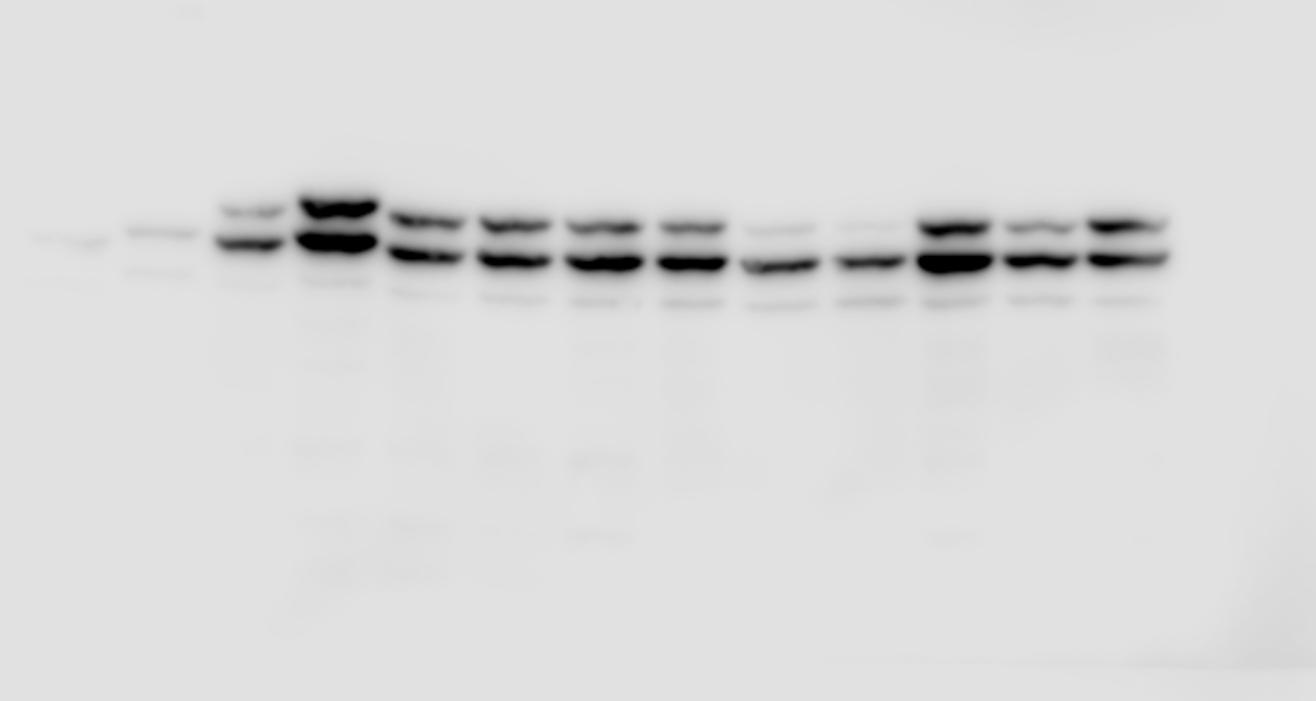

Supplement: Source data 1. [file elife-59999-data1.zip › Raw Cropped blots copy/Figure3_S1C_PhostagM27M59_GFP_VC.tif]

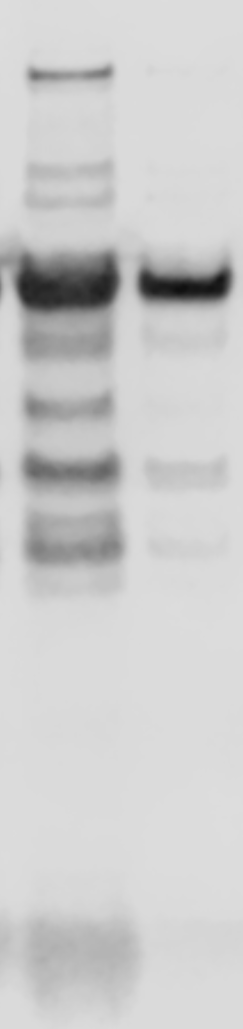

Supplement: Source data 1. [file elife-59999-data1.zip › Raw Cropped blots copy/Figure4C_CHMP7_DD_VC]

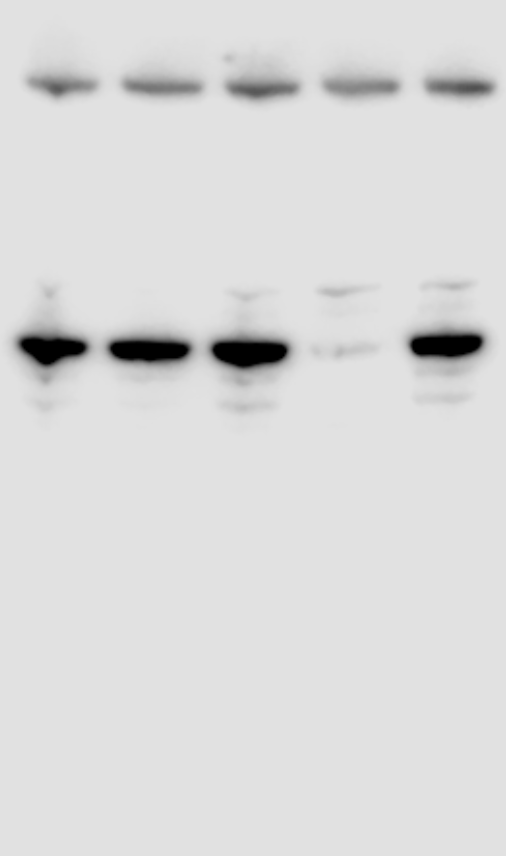

Supplement: Source data 1. [file elife-59999-data1.zip › Raw Cropped blots copy/Figure1B_IST1_VC.tif]

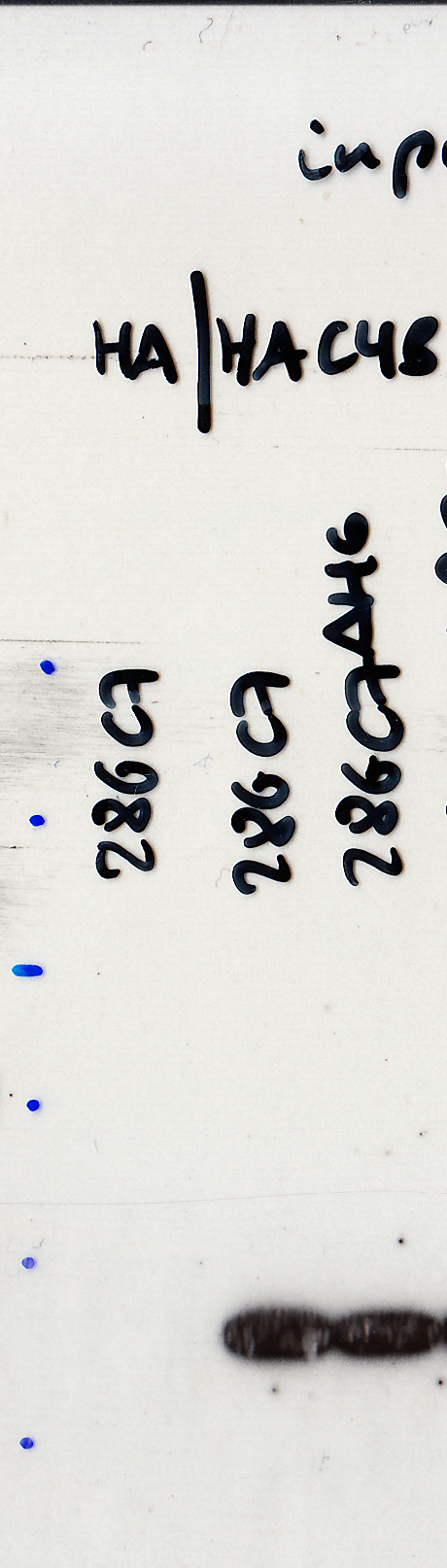

Supplement: Source data 1. [file elife-59999-data1.zip › Raw Cropped blots copy/Figure2_S3C_inputs_HA_VC.tif]

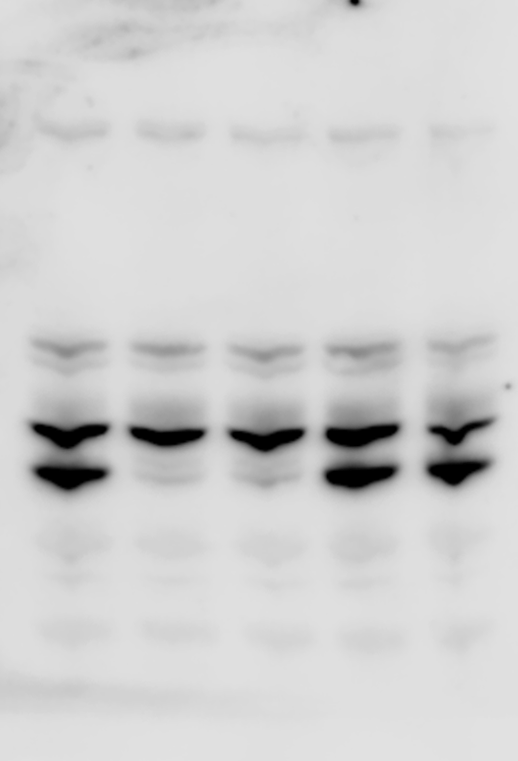

Supplement: Source data 1. [file elife-59999-data1.zip › Raw Cropped blots copy/Figure1B_CHMP7_VC.tif]

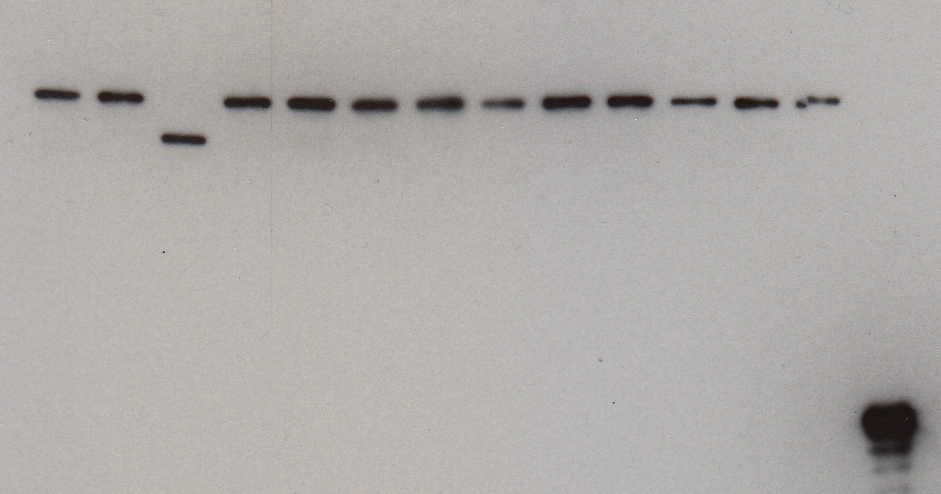

Supplement: Source data 1. [file elife-59999-data1.zip › Raw Cropped blots copy/Figure3_S1A_GFP_PD_VC.tif]

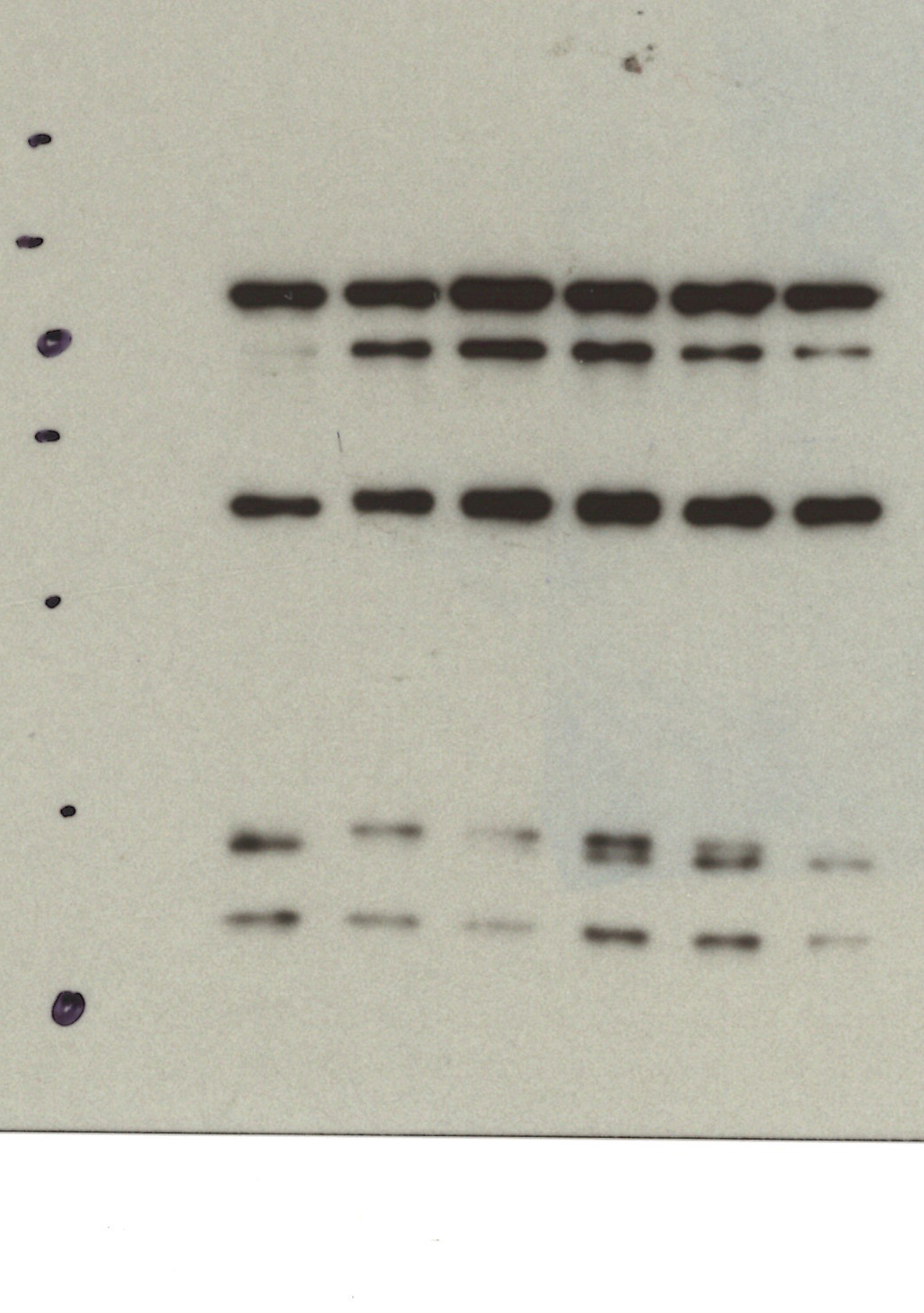

Supplement: Source data 1. [file elife-59999-data1.zip › Raw Cropped blots copy/Figure5E_GFP_Pulldown_VC.tif]

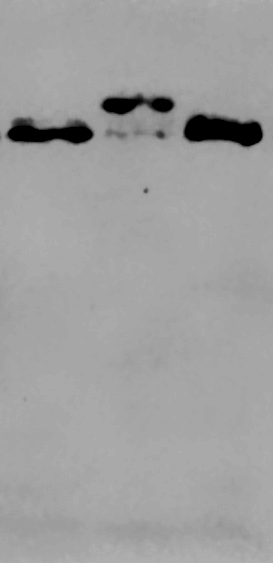

Supplement: Source data 1. [file elife-59999-data1.zip › Raw Cropped blots copy/Figure3C_PhostagGFPmarkers_VC.tif]

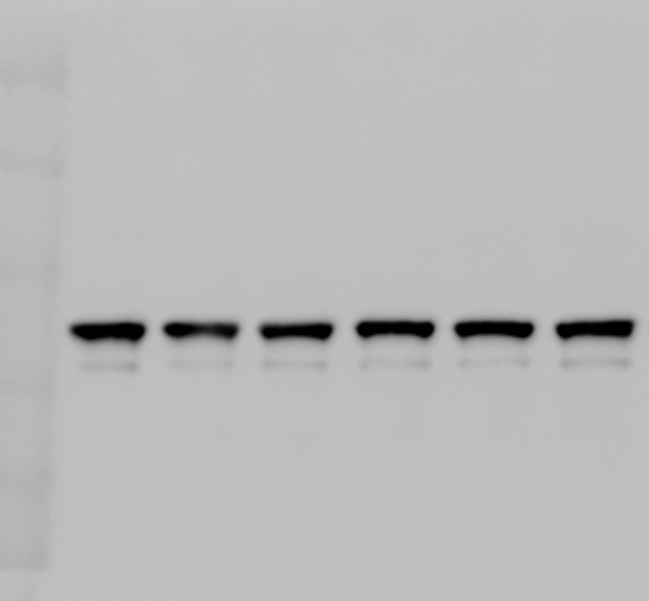

Supplement: Source data 1. [file elife-59999-data1.zip › Raw Cropped blots copy/Figure5D_GAPDH_VC.tif]

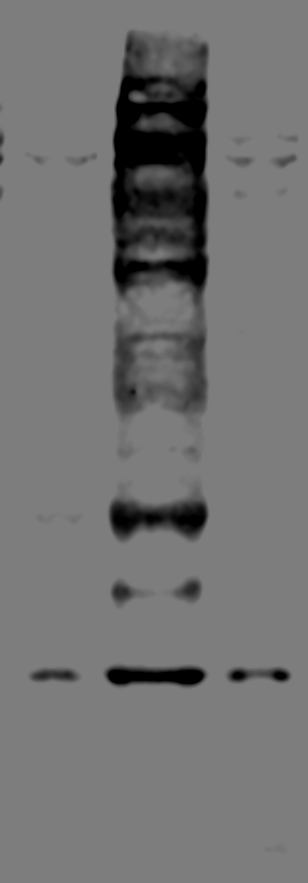

Supplement: Source data 1. [file elife-59999-data1.zip › Raw Cropped blots copy/Figure3C_KH-pS-P_inputs_VC.tif]

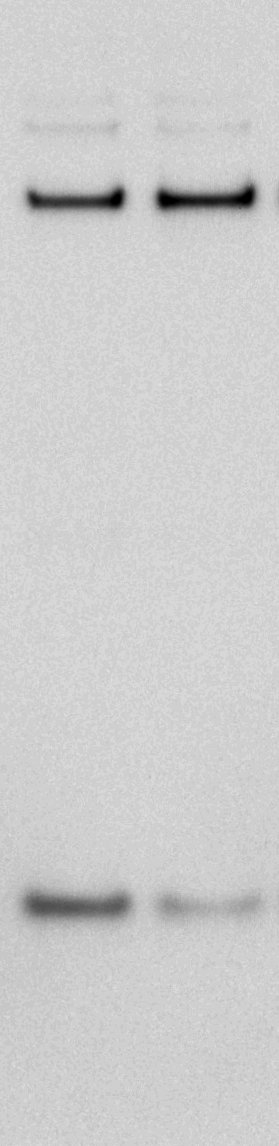

Supplement: Source data 1. [file elife-59999-data1.zip › Raw Cropped blots copy/Figure4_S1C_HALEM2WTC7_HA_VC.tif]

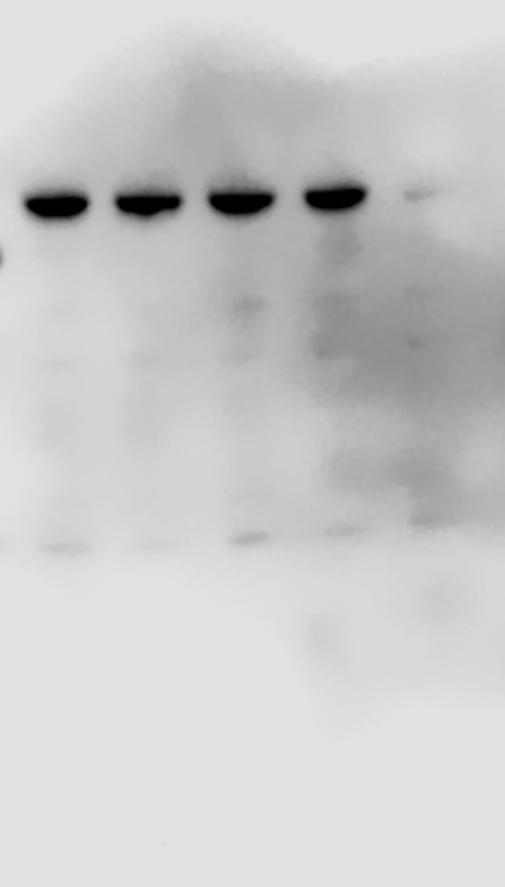

Supplement: Source data 1. [file elife-59999-data1.zip › Raw Cropped blots copy/Figure1B_LEM2_VC.tif]

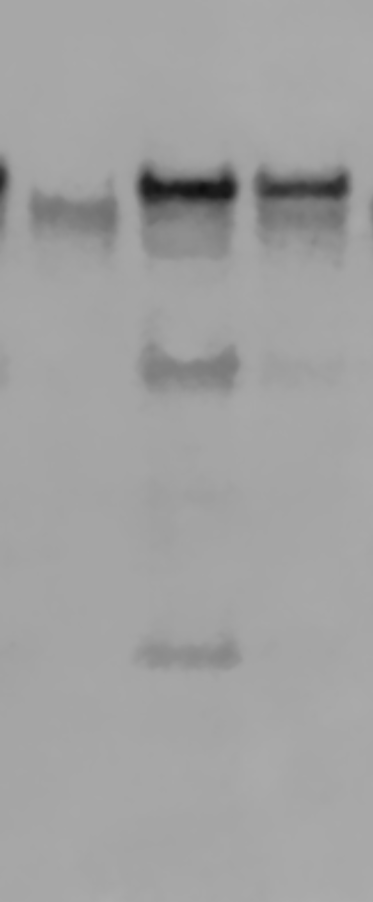

Supplement: Source data 1. [file elife-59999-data1.zip › Raw Cropped blots copy/Figure4G_pulldown_CHMP7_VC]

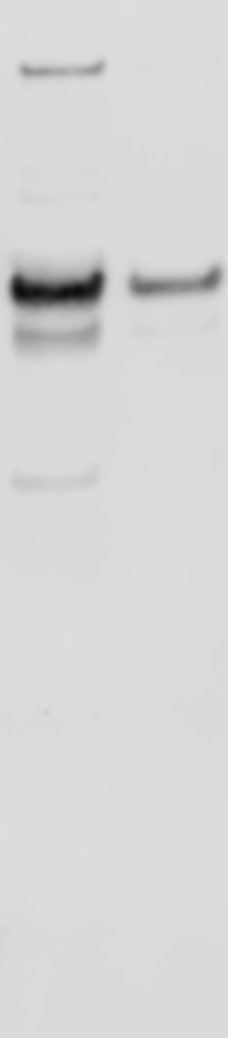

Supplement: Source data 1. [file elife-59999-data1.zip › Raw Cropped blots copy/Figure4C_CHMP7_EE_VC]

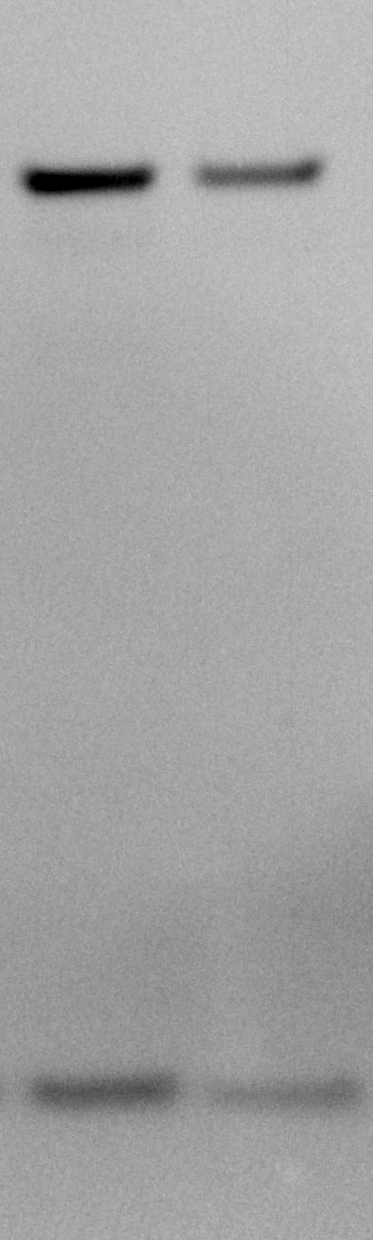

Supplement: Source data 1. [file elife-59999-data1.zip › Raw Cropped blots copy/Figure4E_CHMP7andLEM2_captured_HA_VC.tif]

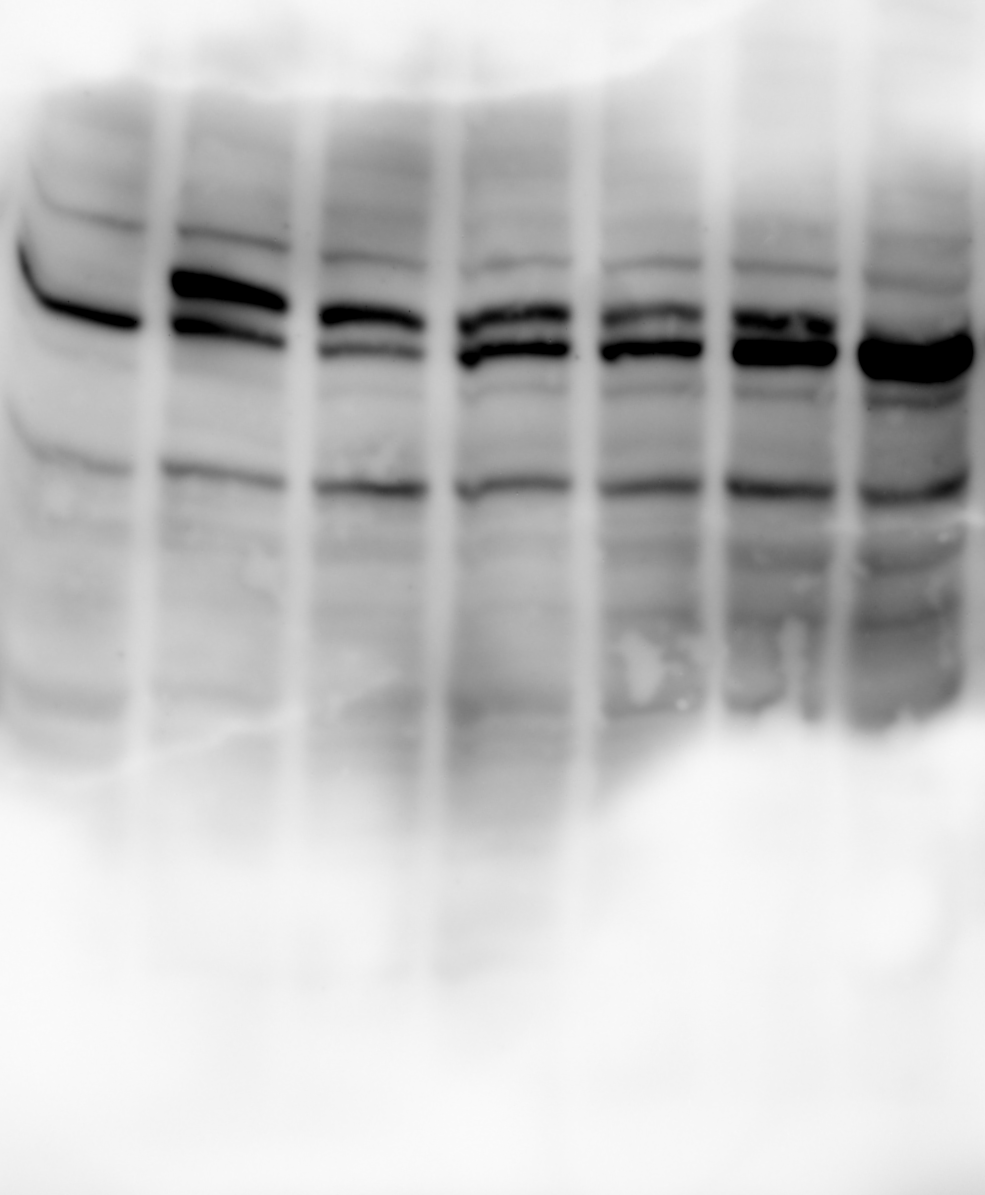

Supplement: Source data 1. [file elife-59999-data1.zip › Raw Cropped blots copy/Figure5_S2B_Phostag_chromatin_GFP_VC.tif]

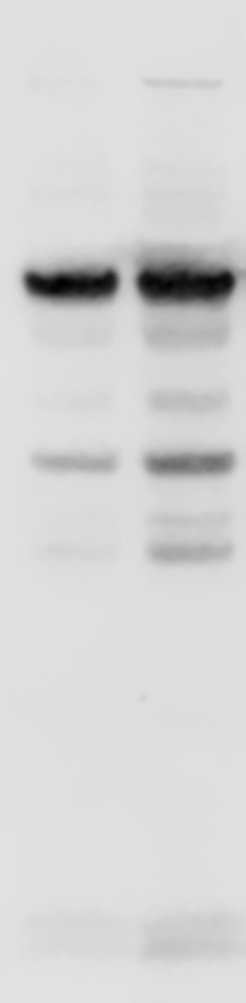

Supplement: Source data 1. [file elife-59999-data1.zip › Raw Cropped blots copy/Figure4_S1A_CHMP7withHAL2CT_CHMP7_VC]

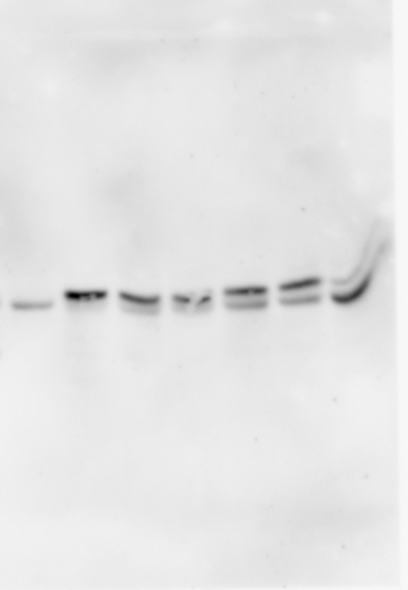

Supplement: Source data 1. [file elife-59999-data1.zip › Raw Cropped blots copy/Figure5_S2B_ERcytosol_VC.tif]

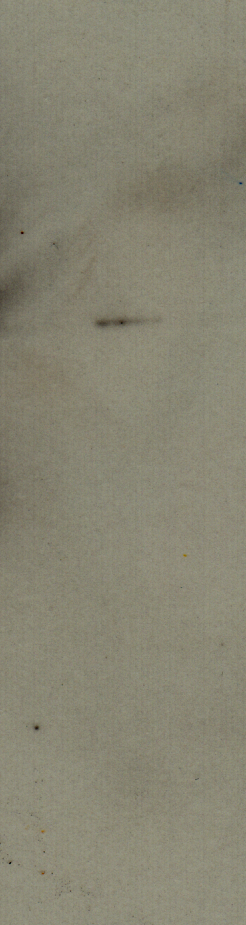

Supplement: Source data 1. [file elife-59999-data1.zip › Raw Cropped blots copy/Figure3C_pS-P-X-KR_PD_VC.tif]

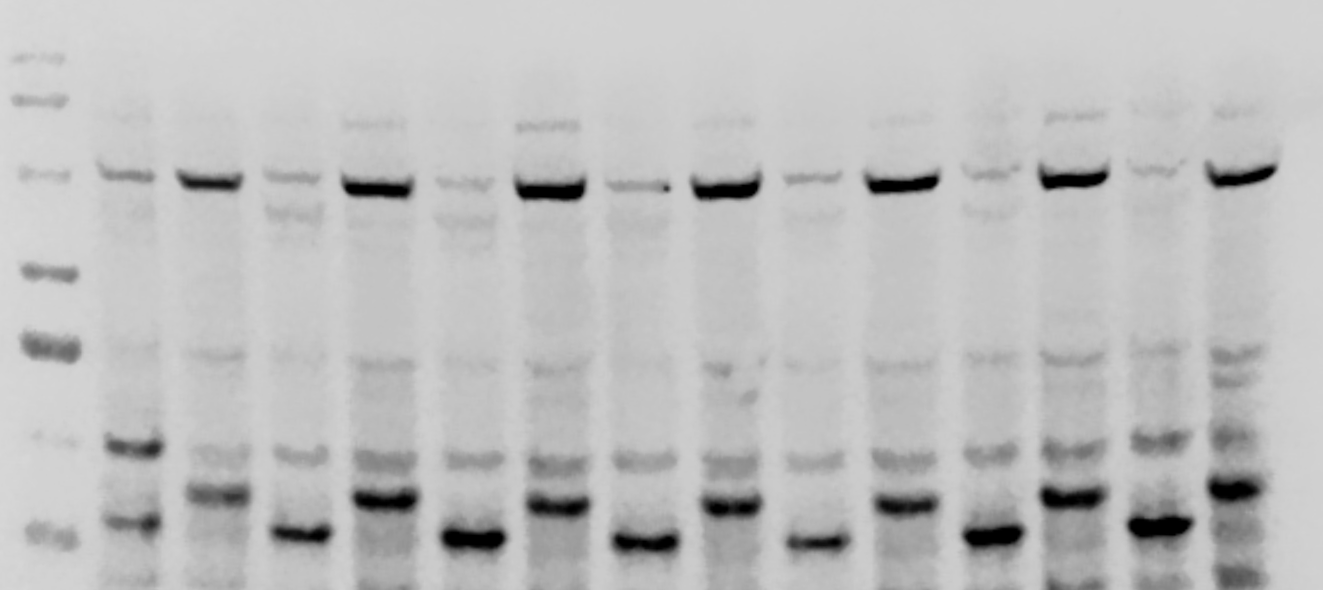

Supplement: Source data 1. [file elife-59999-data1.zip › Raw Cropped blots copy/Figure5_S2A_GFP_VC.tif]

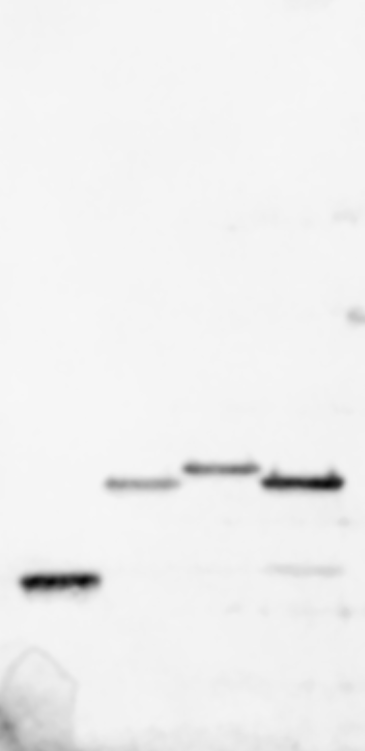

Supplement: Source data 1. [file elife-59999-data1.zip › Raw Cropped blots copy/Figure2_S2B_Pulldown_GST_VC]

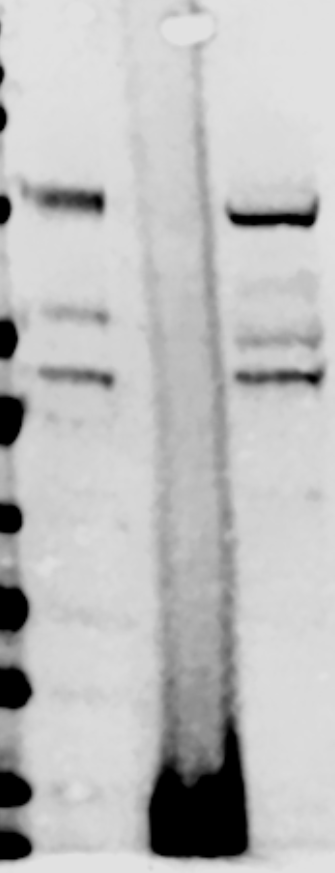

Supplement: Source data 1. [file elife-59999-data1.zip › Raw Cropped blots copy/Figure1 S2A_mCherry_markers_VC.tif]

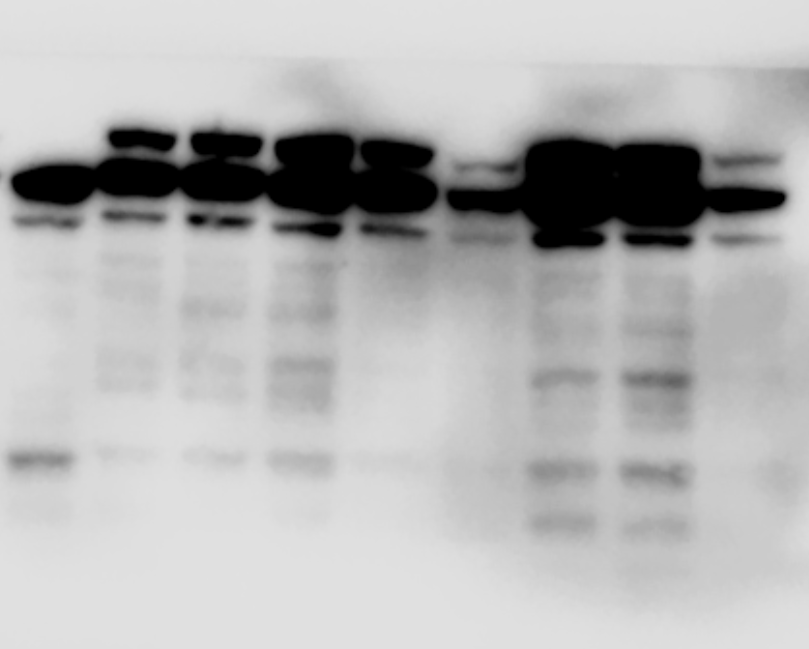

Supplement: Source data 1. [file elife-59999-data1.zip › Raw Cropped blots copy/Figure3_S1C_PhostagM1M23_GFP_darker_VC.tif]

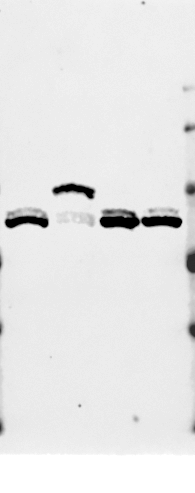

Supplement: Source data 1. [file elife-59999-data1.zip › Raw Cropped blots copy/Figure3D_GFP_Phostag_VC.tif]

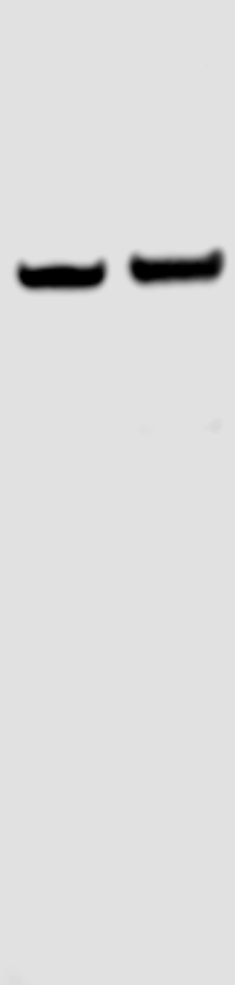

Supplement: Source data 1. [file elife-59999-data1.zip › Raw Cropped blots copy/Figure4E_CHMP7andLEM2_input_CHMP7_VC]

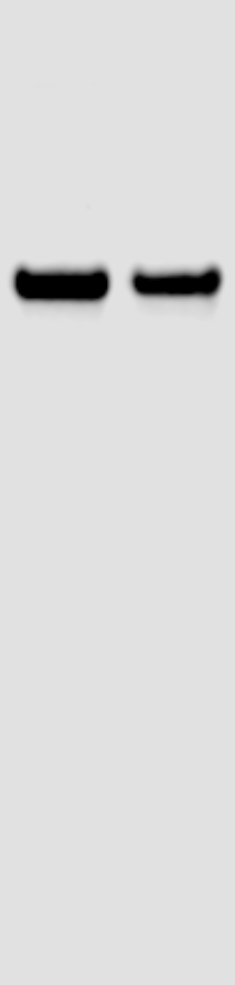

Supplement: Source data 1. [file elife-59999-data1.zip › Raw Cropped blots copy/Figure4E_CHMP7andLEM2_captured_CHMP7_VC.tif]

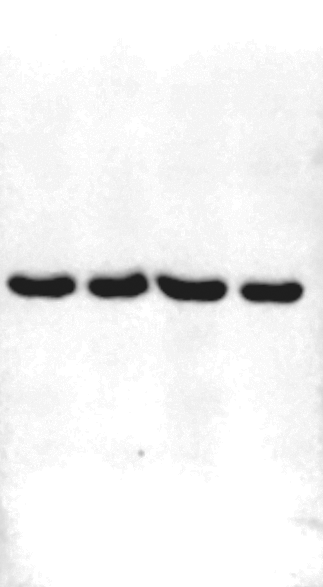

Supplement: Source data 1. [file elife-59999-data1.zip › Raw Cropped blots copy/Figure3D_GAPDH_VC.tif]

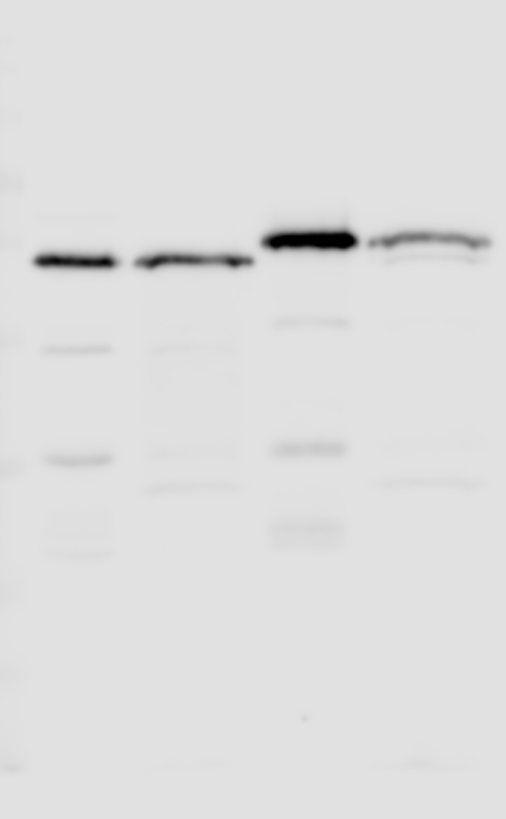

Supplement: Source data 1. [file elife-59999-data1.zip › Raw Cropped blots copy/Figure4A_CHMP7_VC]

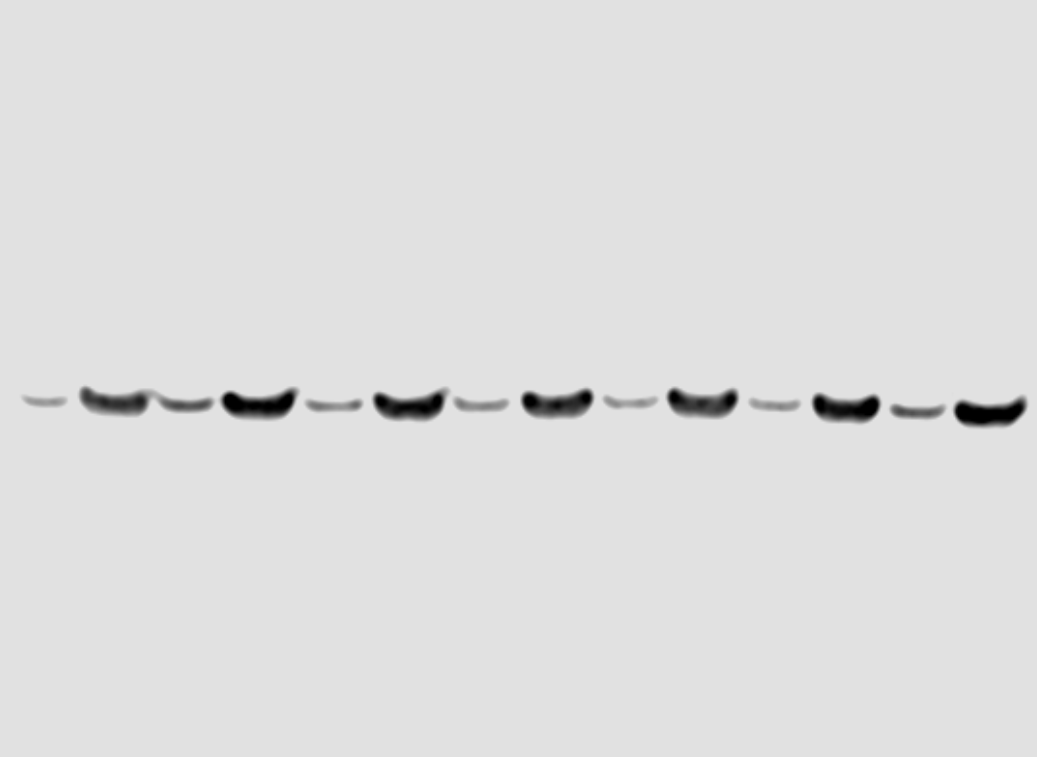

Supplement: Source data 1. [file elife-59999-data1.zip › Raw Cropped blots copy/Figure5_S2A_G3PDH_VC.tif]

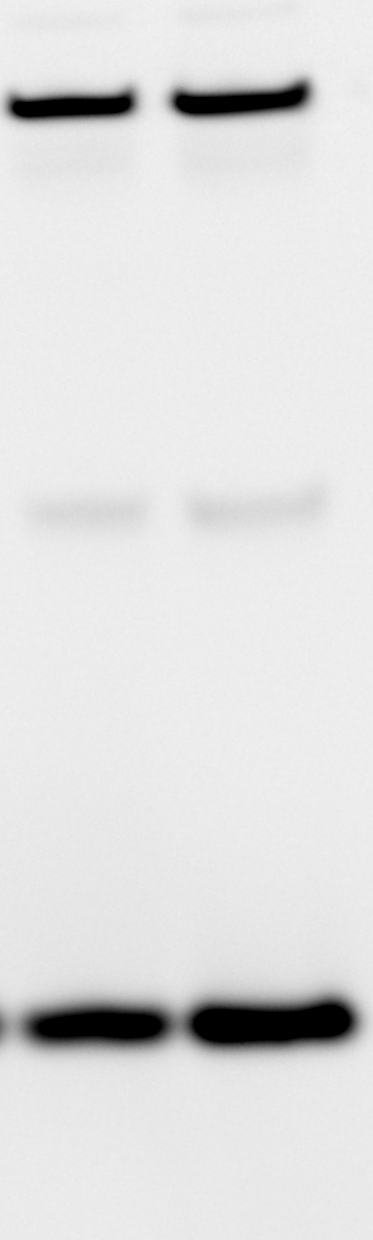

Supplement: Source data 1. [file elife-59999-data1.zip › Raw Cropped blots copy/Figure4E_CHMP7andLEM2_input_HA_VC]

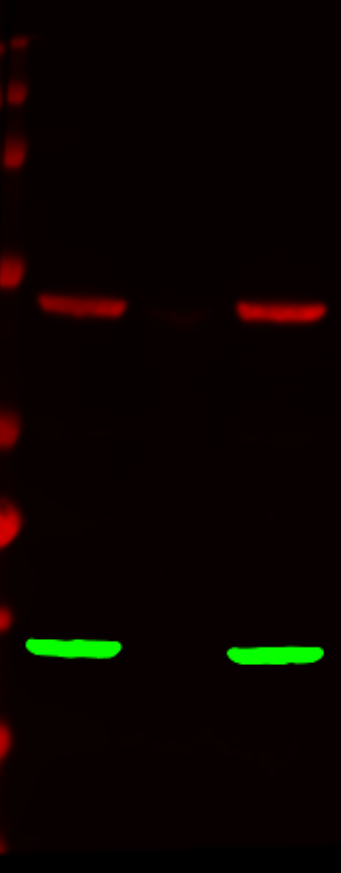

Supplement: Source data 1. [file elife-59999-data1.zip › Raw Cropped blots copy/Figure1_S2A_GAPDH_markers_CNXunnused_VC.tif]

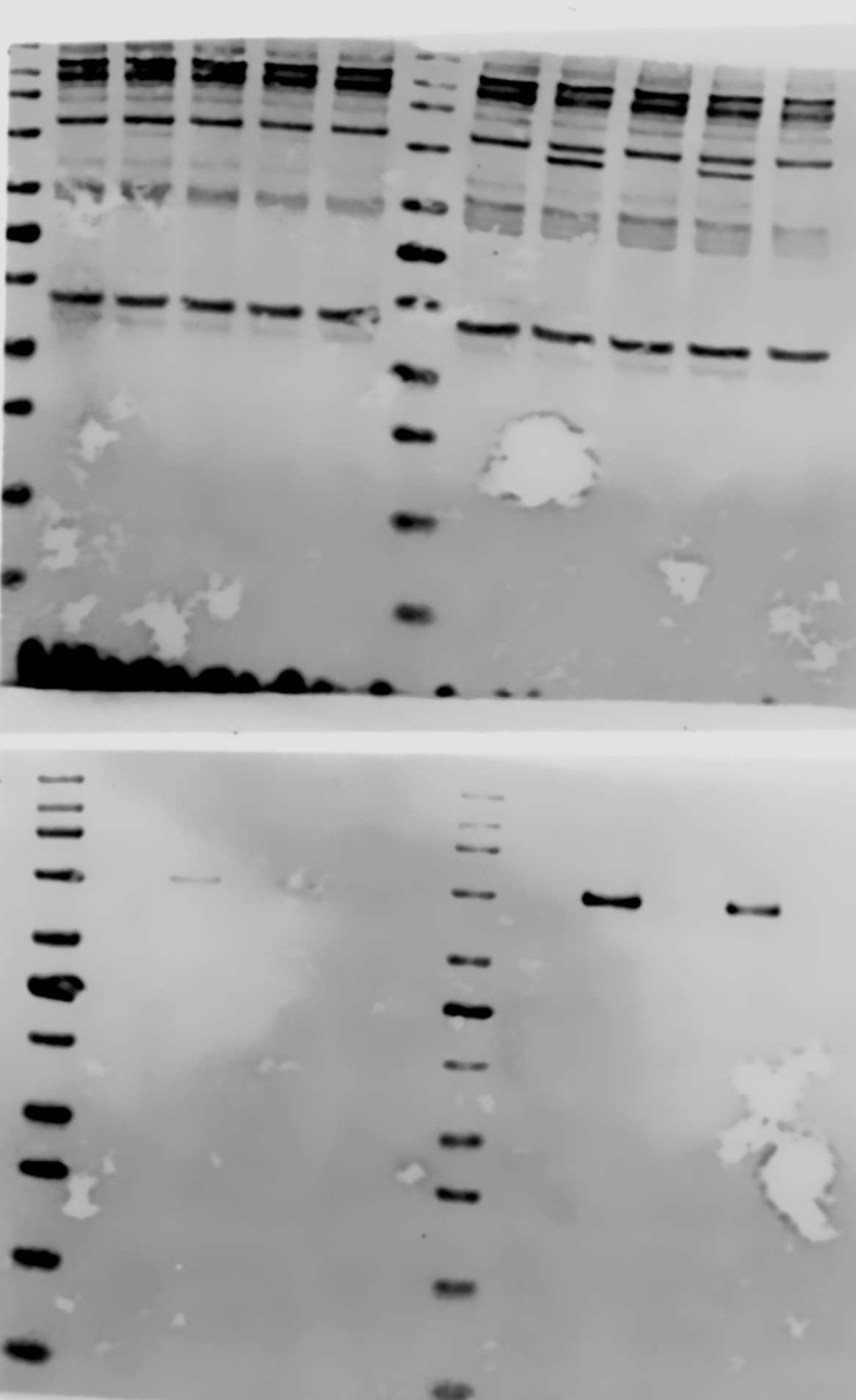

Supplement: Source data 1. [file elife-59999-data1.zip › Raw Cropped blots copy/Figure5_S2D_3891_IPtop_GTrapbottom_VC.tif]

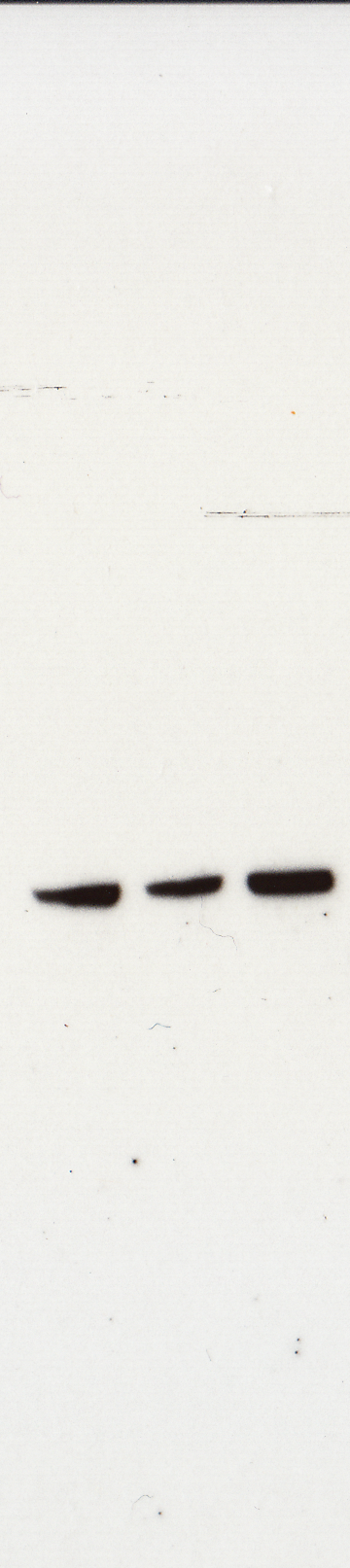

Supplement: Source data 1. [file elife-59999-data1.zip › Raw Cropped blots copy/Figure2_S3C_input_GFP_VC.tif]

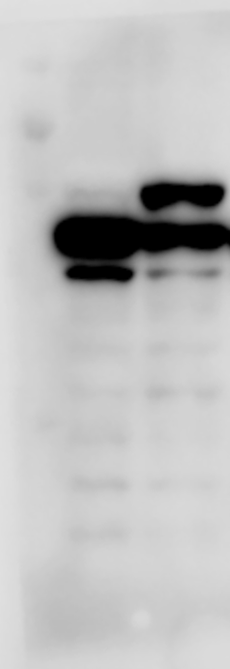

Supplement: Source data 1. [file elife-59999-data1.zip › Raw Cropped blots copy/Figure3_S1C_PhostagInterphaseMitosis_VC.tif]

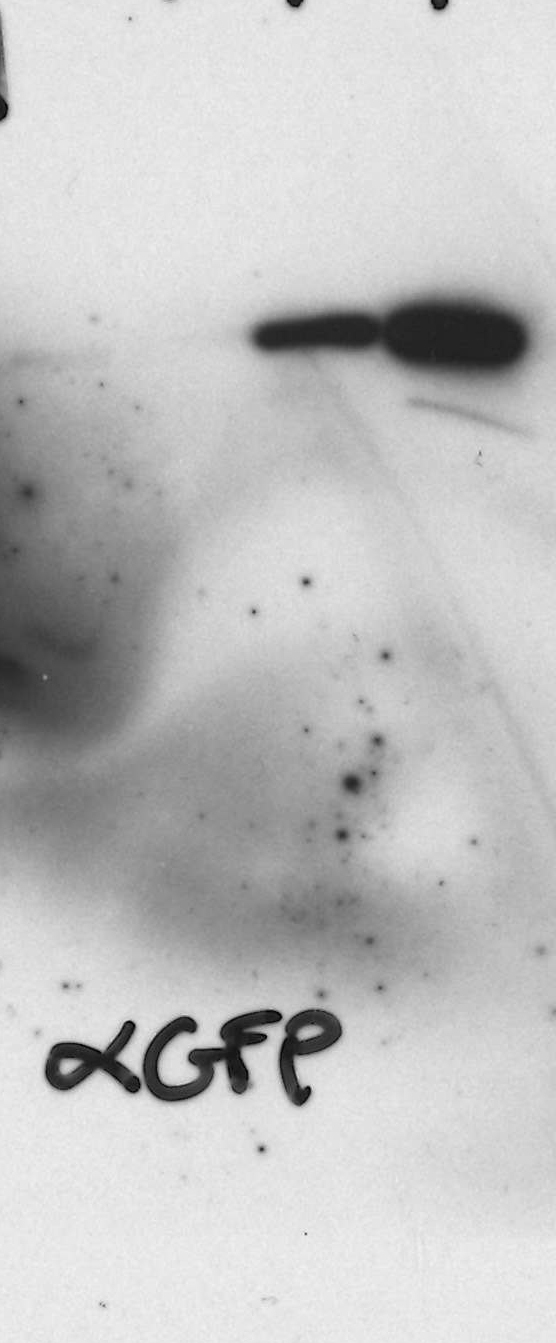

Supplement: Source data 1. [file elife-59999-data1.zip › Raw Cropped blots copy/Figure2_S2B_Pulldown_GFP_VC.jpg]

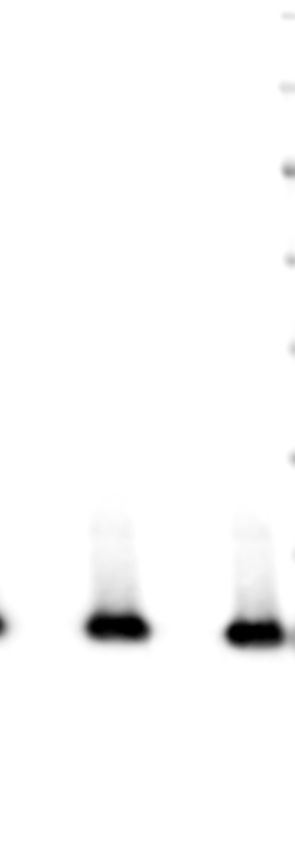

Supplement: Source data 1. [file elife-59999-data1.zip › Raw Cropped blots copy/Figure3_S1B_pH3_VC.tif]

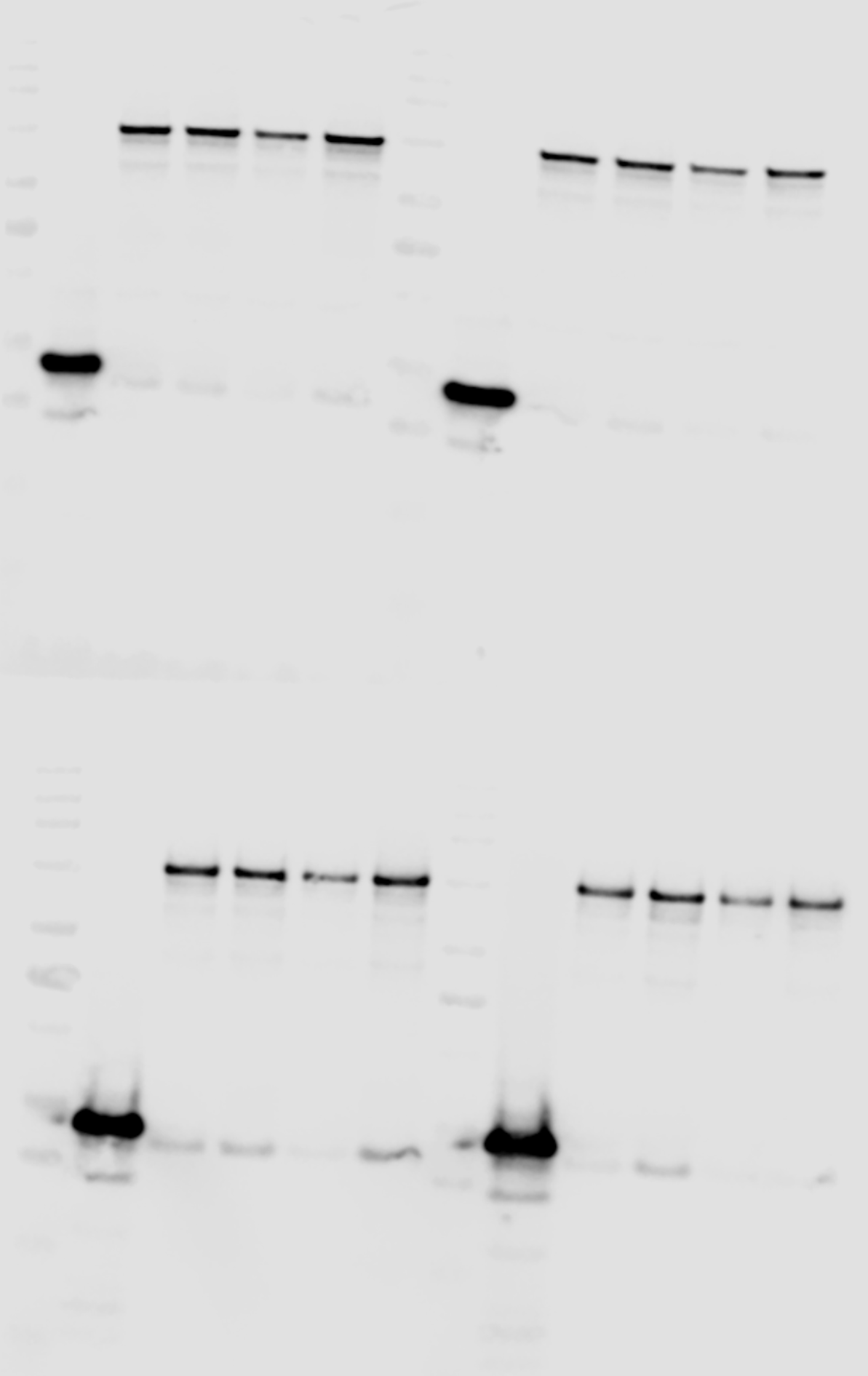

Supplement: Source data 1. [file elife-59999-data1.zip › Raw Cropped blots copy/Figure5_S2D_3891_IPtop_GTrapbottom_GFP_VC.tif]

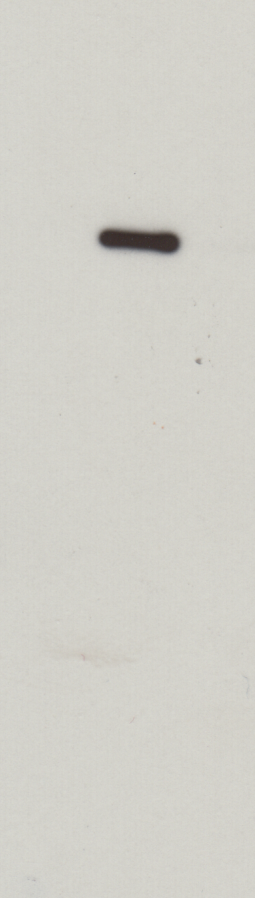

Supplement: Source data 1. [file elife-59999-data1.zip › Raw Cropped blots copy/Figure3C_KH-pS-P_PD_VC.tif]

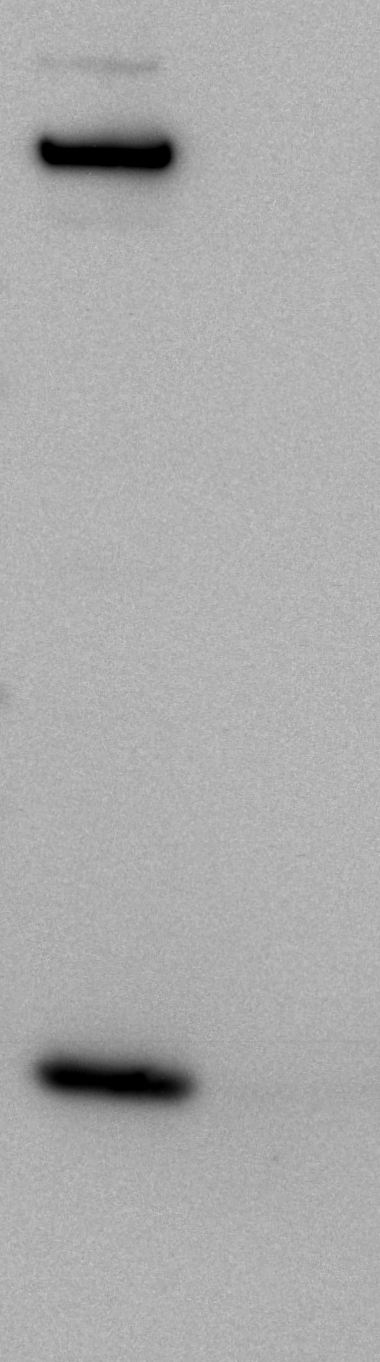

Supplement: Source data 1. [file elife-59999-data1.zip › Raw Cropped blots copy/Figure4_S1A_LEM2alone_HA_VC]

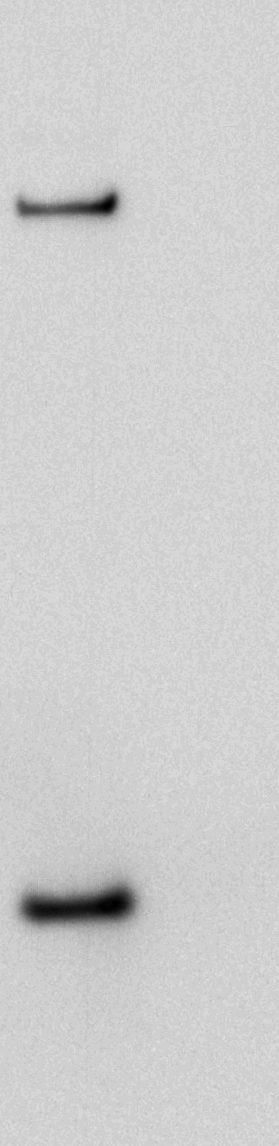

Supplement: Source data 1. [file elife-59999-data1.zip › Raw Cropped blots copy/Figure4_S1C_HALEM2_C7EE_HA_VC.tif]

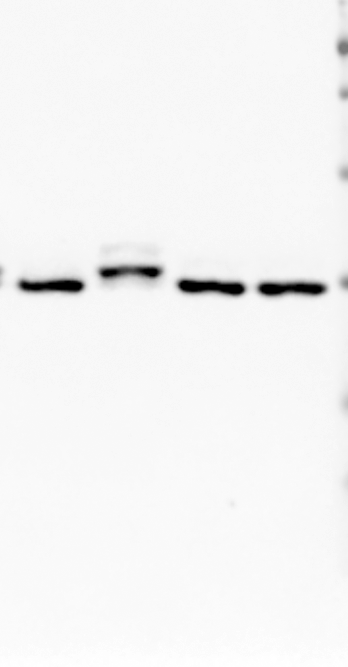

Supplement: Source data 1. [file elife-59999-data1.zip › Raw Cropped blots copy/Figure3_S1B_GFPphostag_VC.tif]

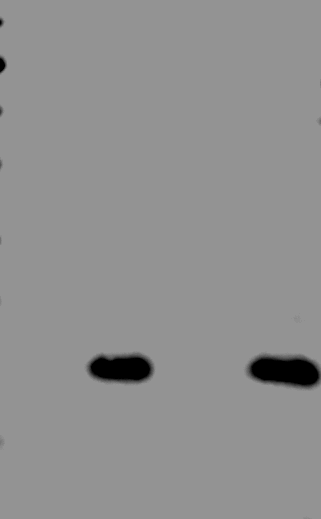

Supplement: Source data 1. [file elife-59999-data1.zip › Raw Cropped blots copy/Figure3D_pH3_VC.tif]

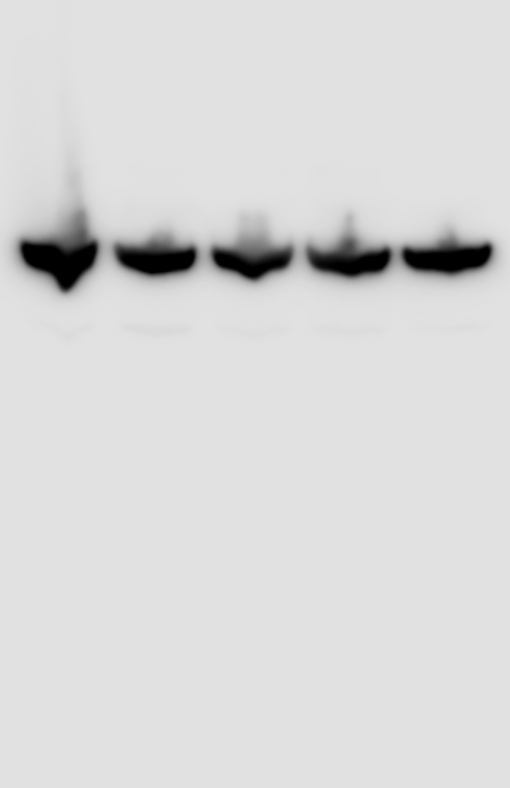

Supplement: Source data 1. [file elife-59999-data1.zip › Raw Cropped blots copy/Figure1B_HSP90_VC.tif]

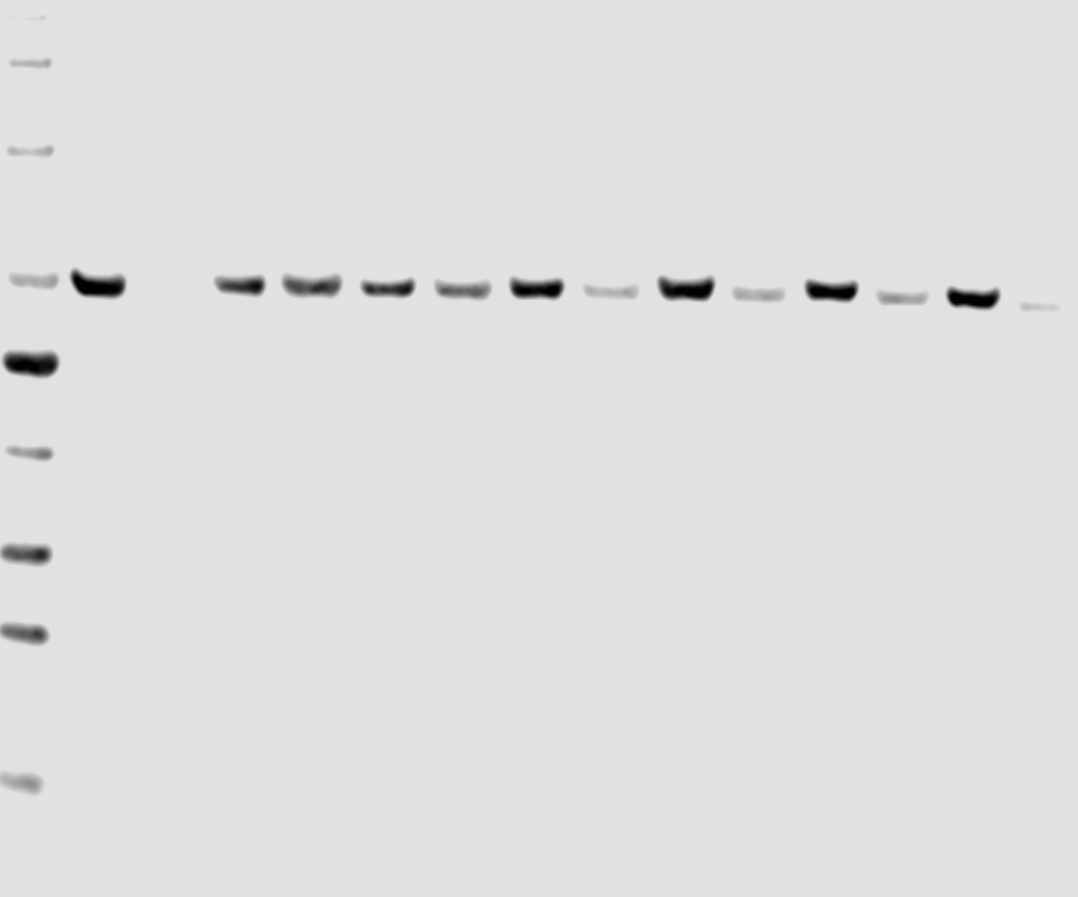

Supplement: Source data 1. [file elife-59999-data1.zip › Raw Cropped blots copy/FIgure5_S2A_LAP1_VC.tif]

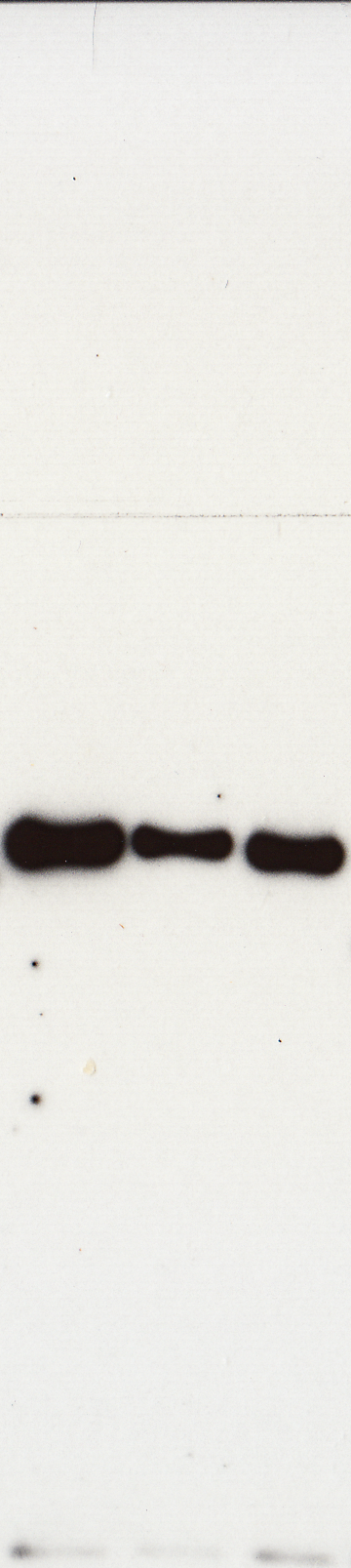

Supplement: Source data 1. [file elife-59999-data1.zip › Raw Cropped blots copy/Figure2_S3C_pulldown_GFP_VC.tif]

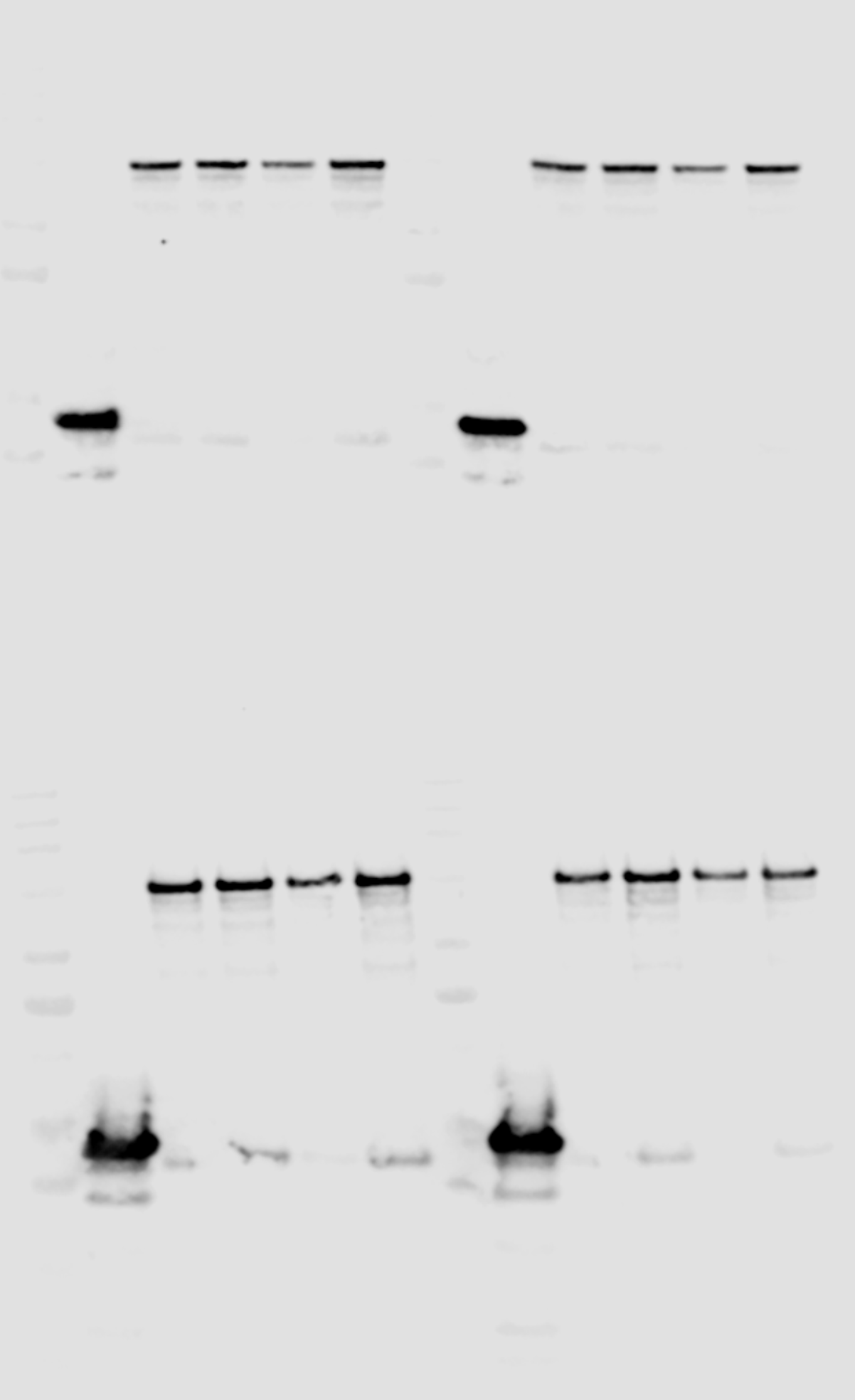

Supplement: Source data 1. [file elife-59999-data1.zip › Raw Cropped blots copy/Figure5_S2D_3892_IPtop_GTrapbottom_GFP_VC.tif]

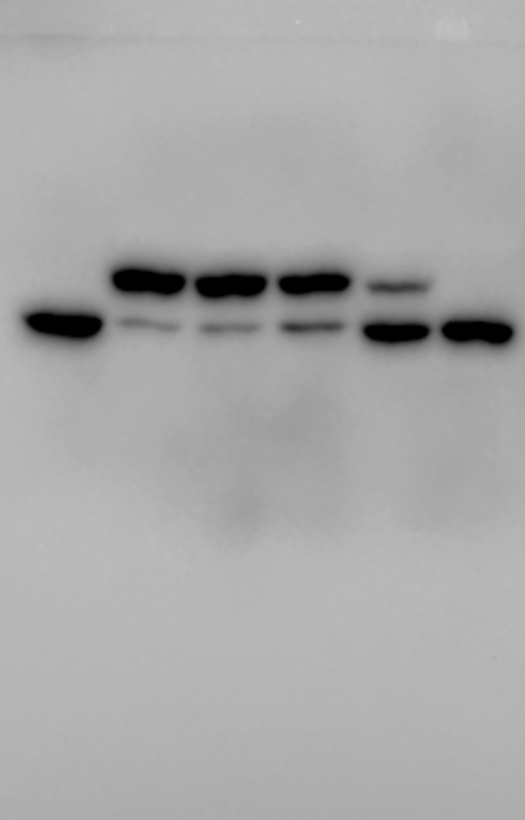

Supplement: Source data 1. [file elife-59999-data1.zip › Raw Cropped blots copy/Figure5D_Phostag_GFP_VC.tif]

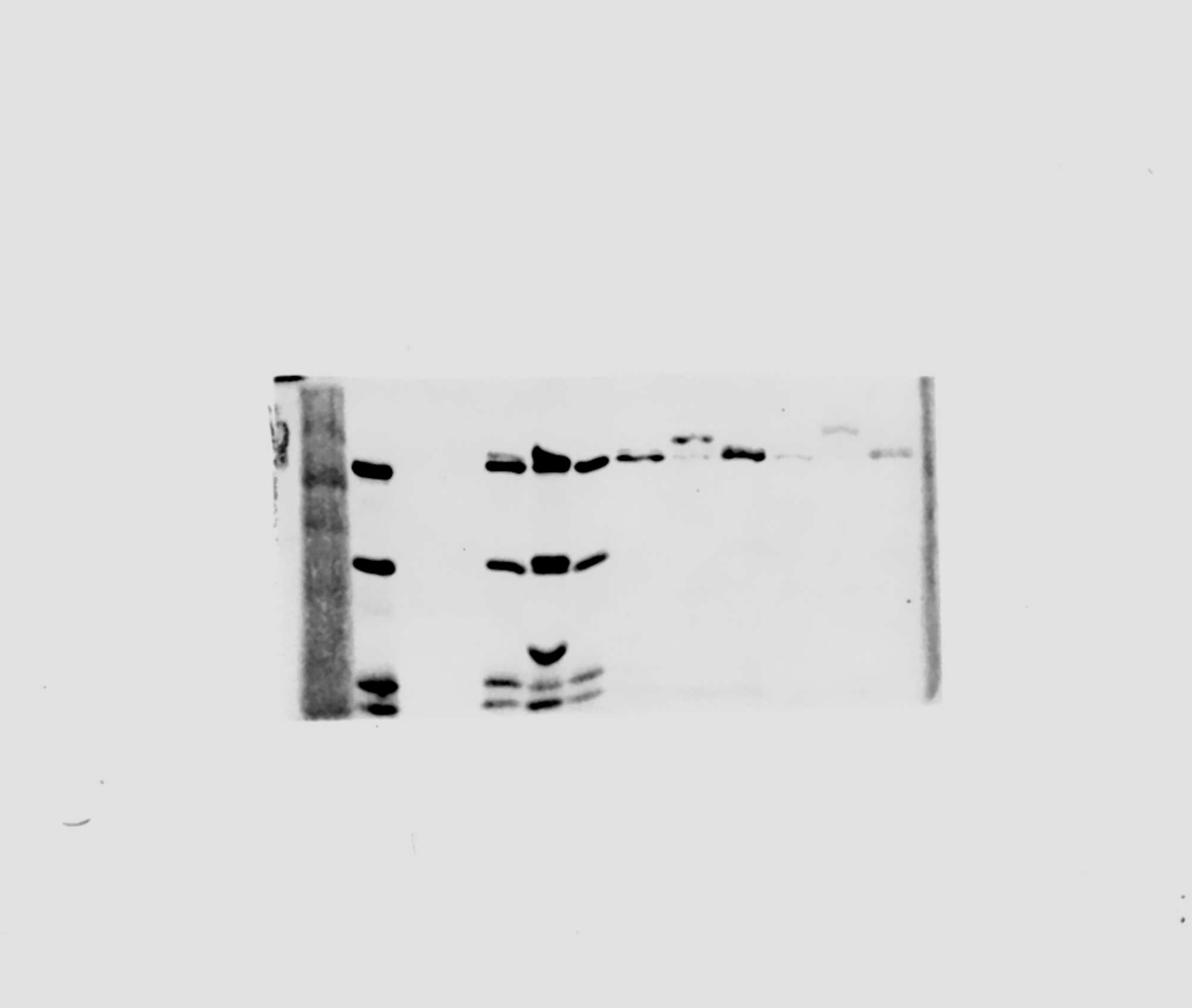

Supplement: Source data 2. [file elife-59999-data2.zip › Raw Unedited blots copy/Figure3C_PhostagGFPmarkers.tif]

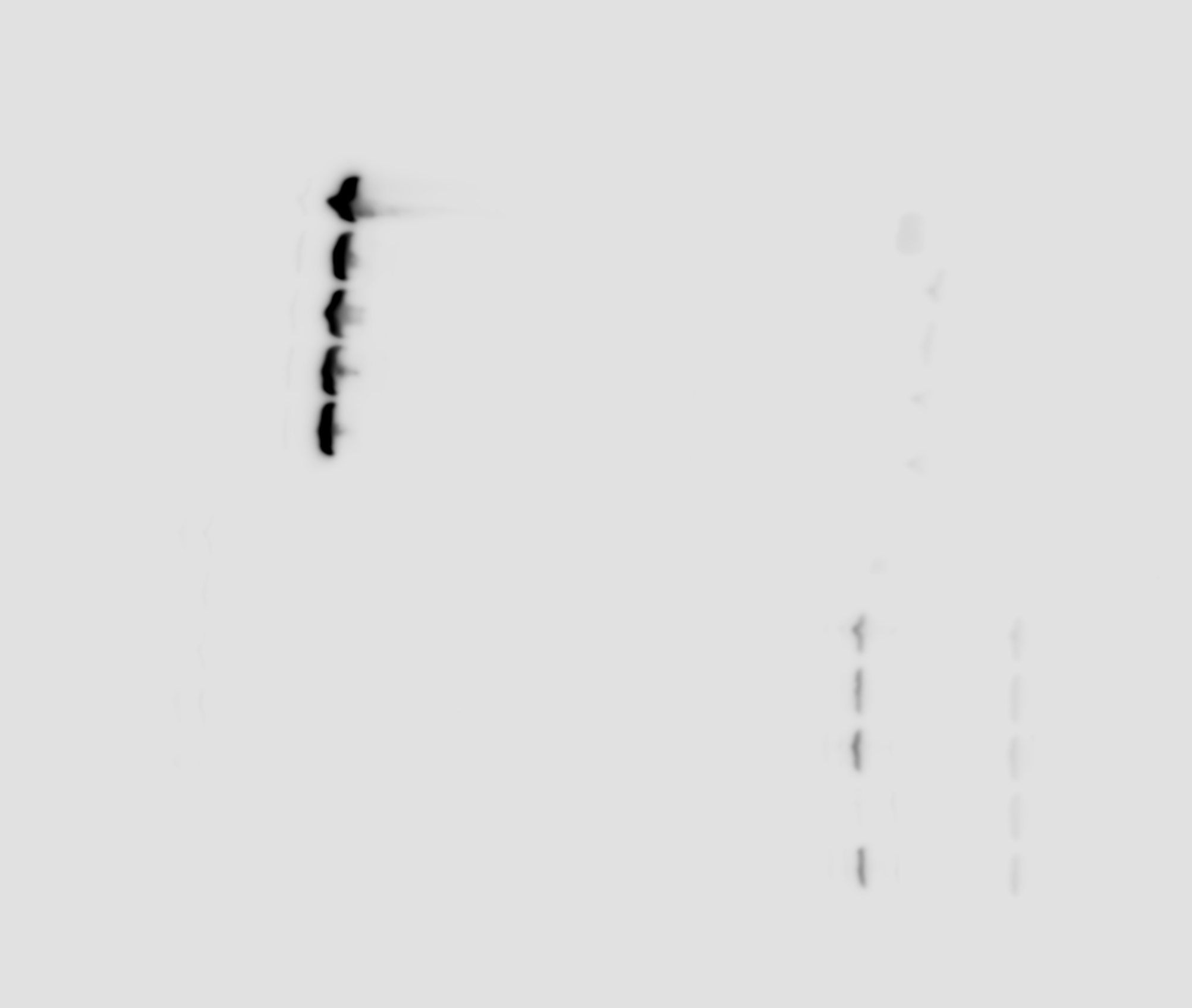

Supplement: Source data 2. [file elife-59999-data2.zip › Raw Unedited blots copy/Figure1B_HSP90.tif]

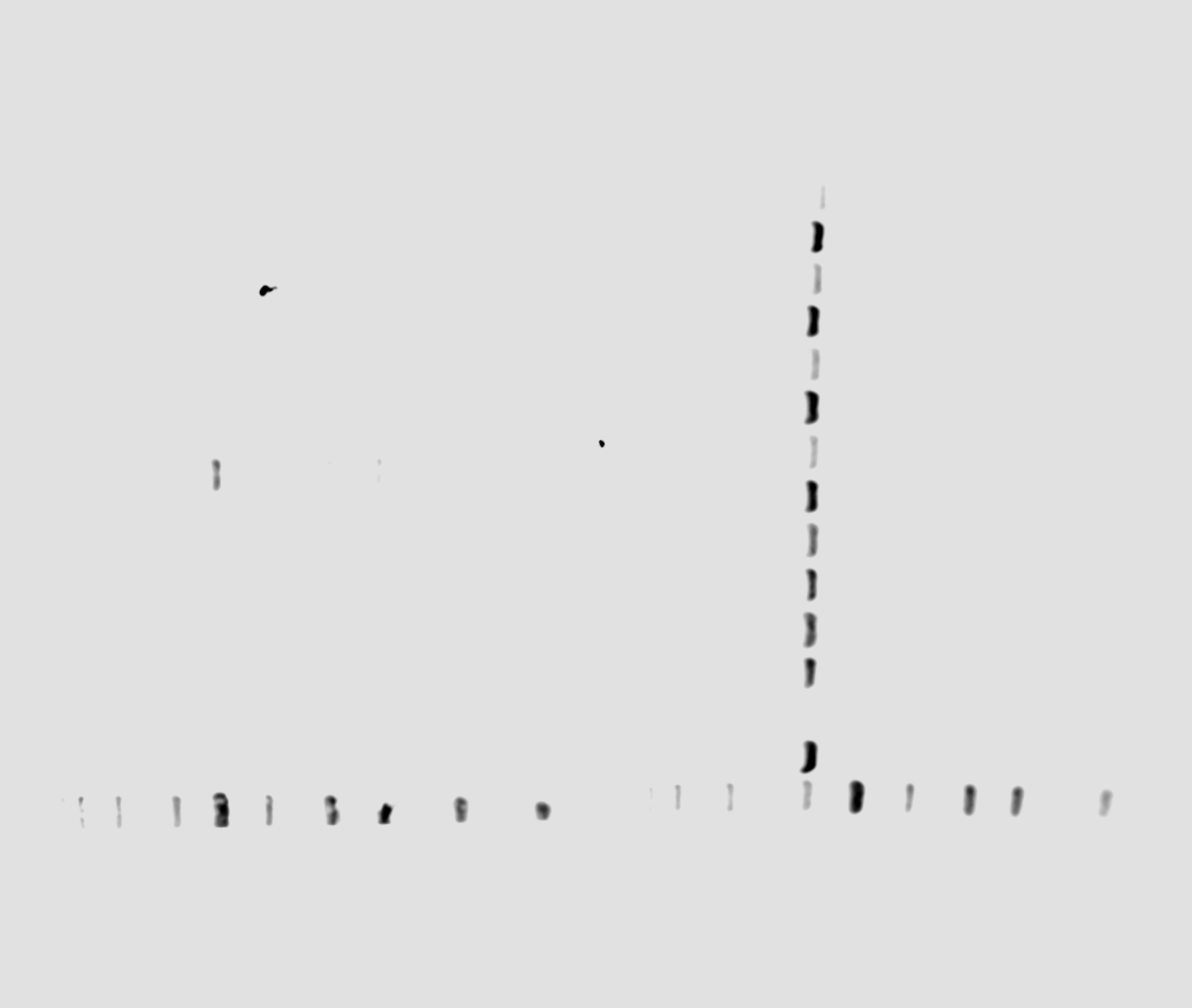

Supplement: Source data 2. [file elife-59999-data2.zip › Raw Unedited blots copy/FIgure5_S2A_LAP1.tif]

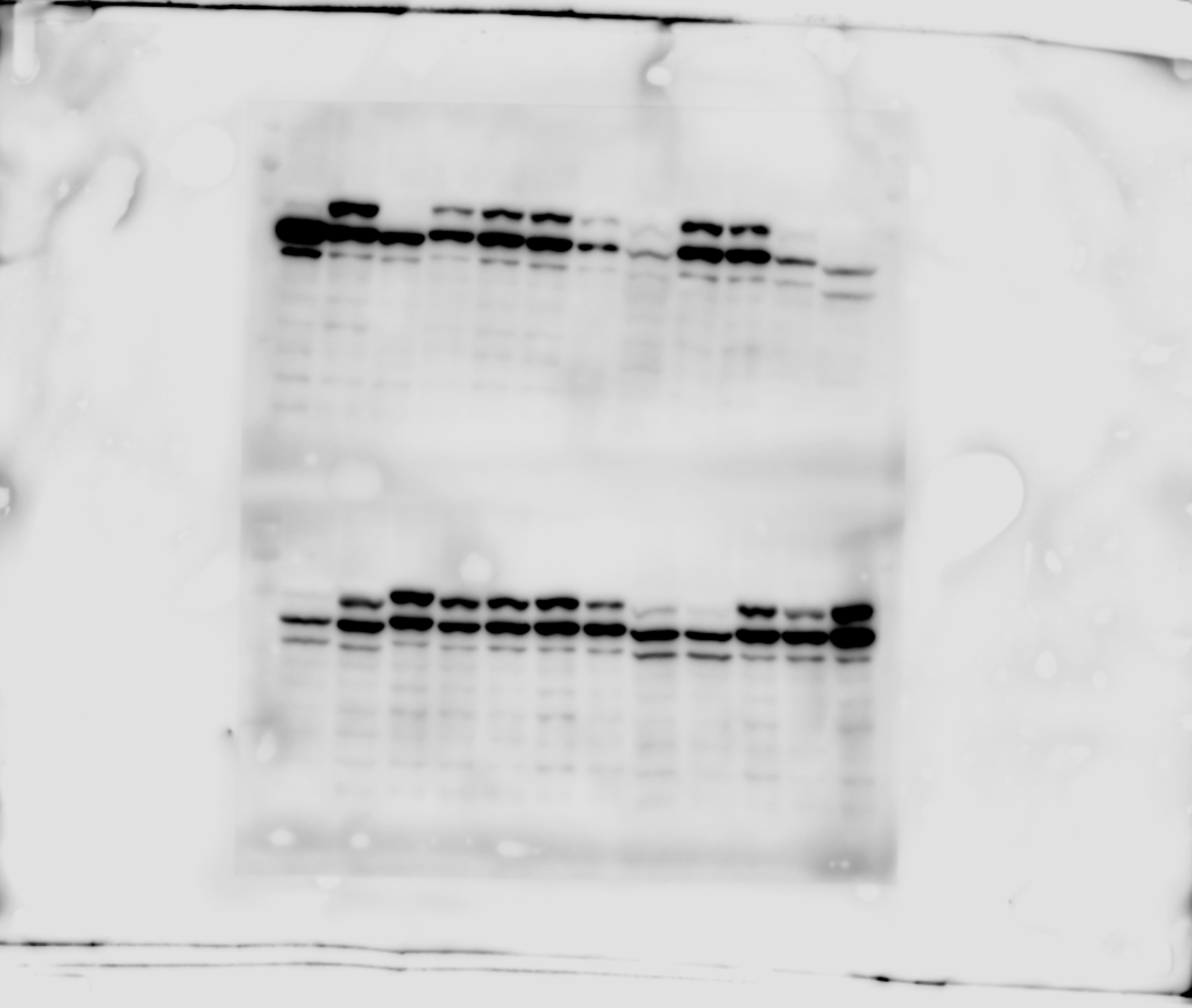

Supplement: Source data 2. [file elife-59999-data2.zip › Raw Unedited blots copy/Figure3_S1C_Interphase_Mitosis.tif]

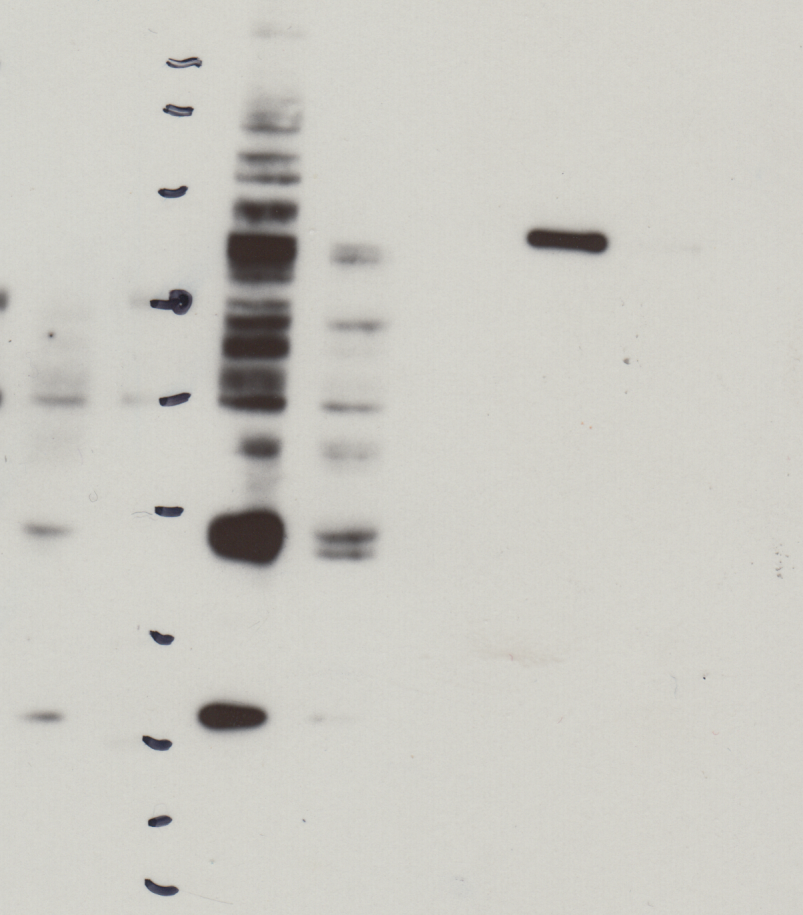

Supplement: Source data 2. [file elife-59999-data2.zip › Raw Unedited blots copy/Figure3C_KH-pS-P_PD.tiff]

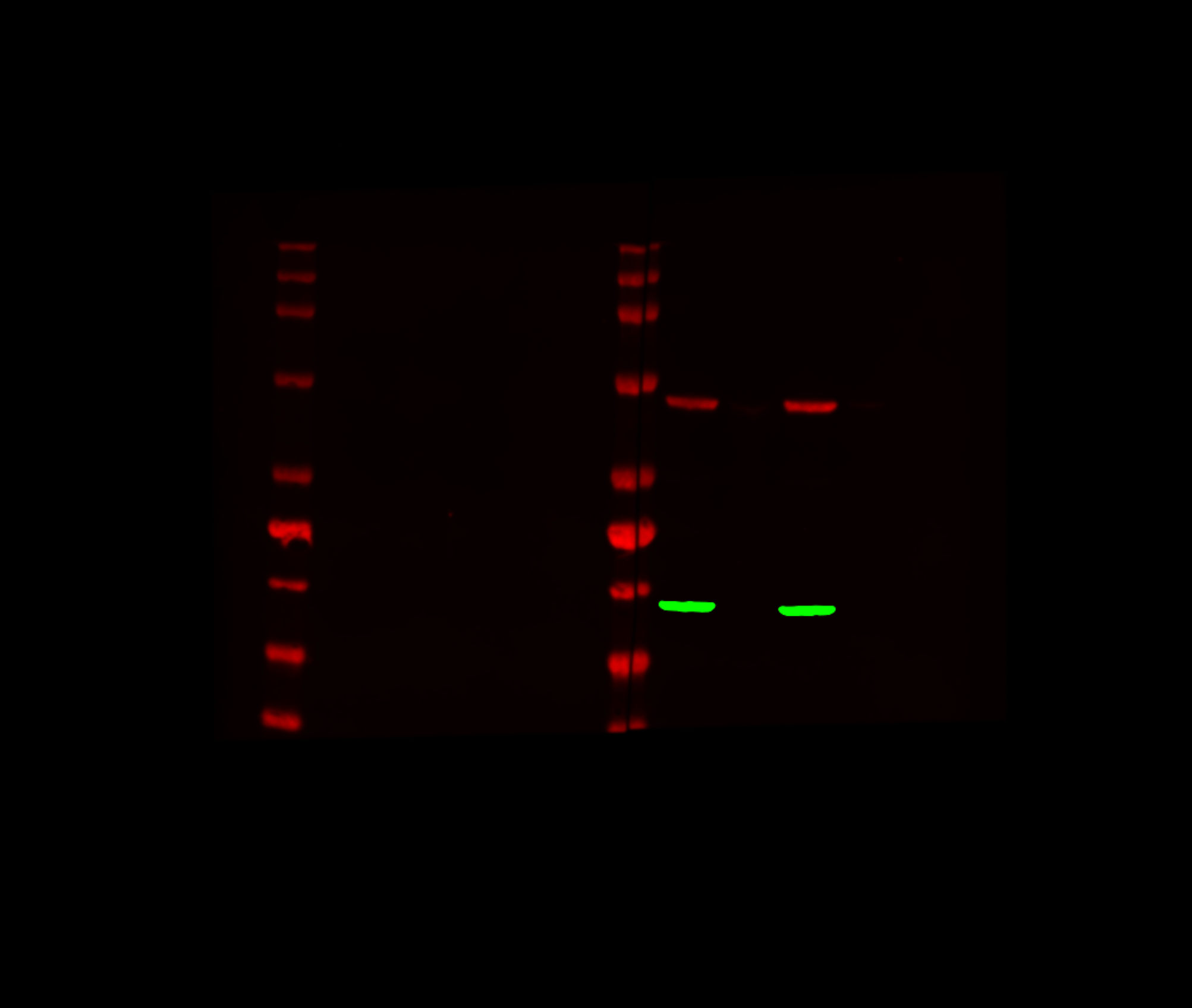

Supplement: Source data 2. [file elife-59999-data2.zip › Raw Unedited blots copy/Figure1_S2A_GAPDH_markers_CNXunnused.tif]

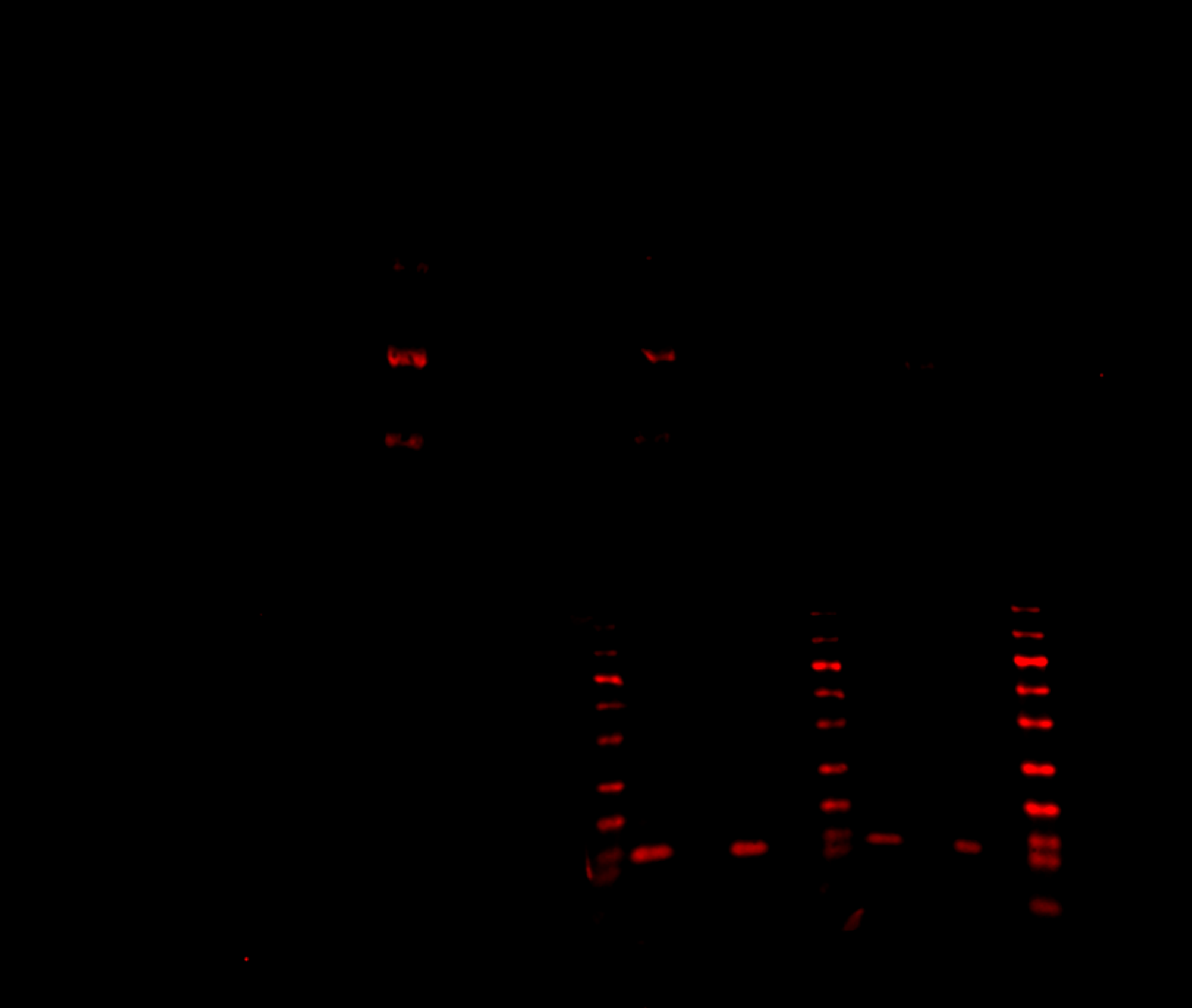

Supplement: Source data 2. [file elife-59999-data2.zip › Raw Unedited blots copy/Figure3D_pSer10HistoneH3_markers.tif]

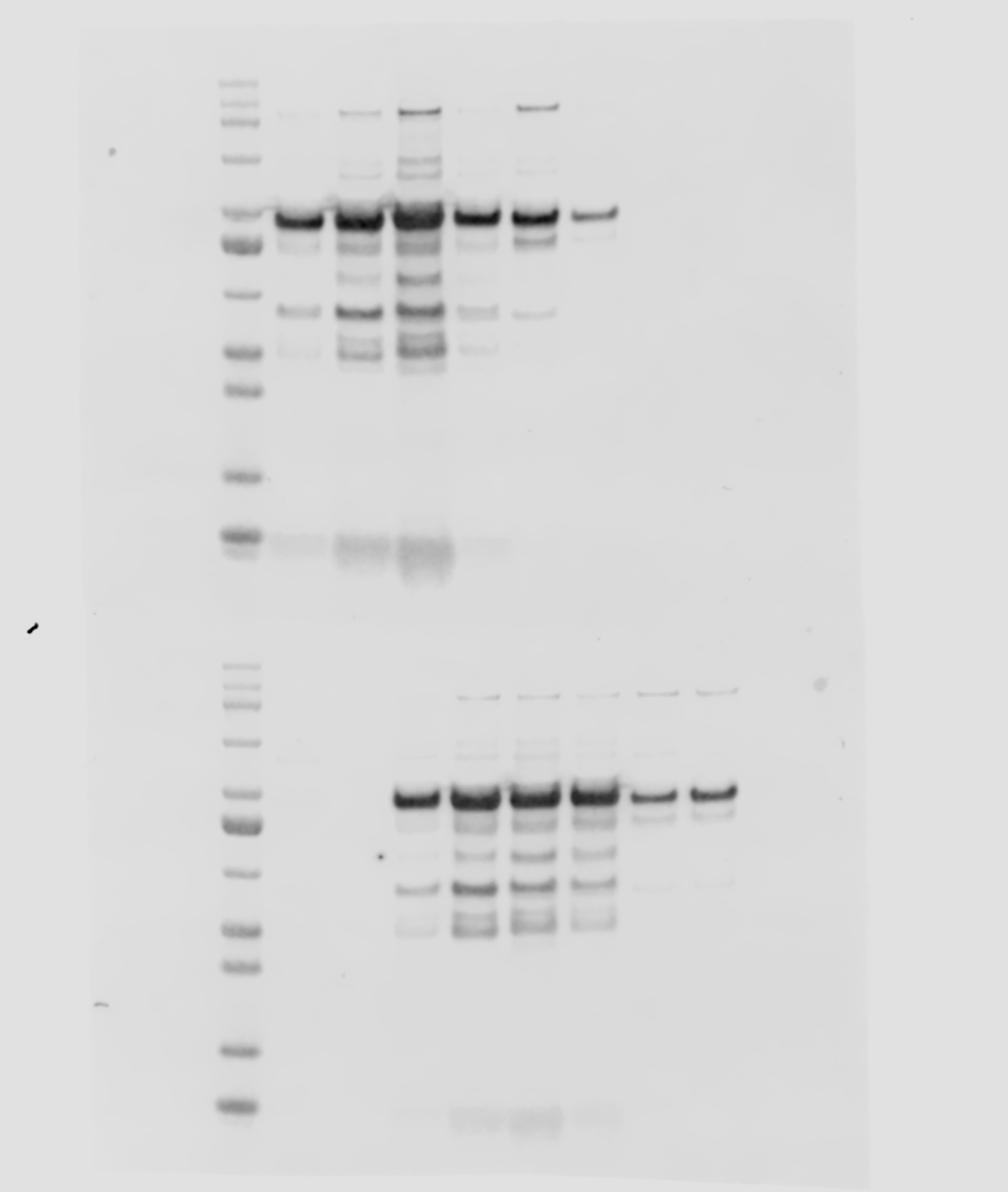

Supplement: Source data 2. [file elife-59999-data2.zip › Raw Unedited blots copy/Figure4C_CHMP7_topblot.tif]

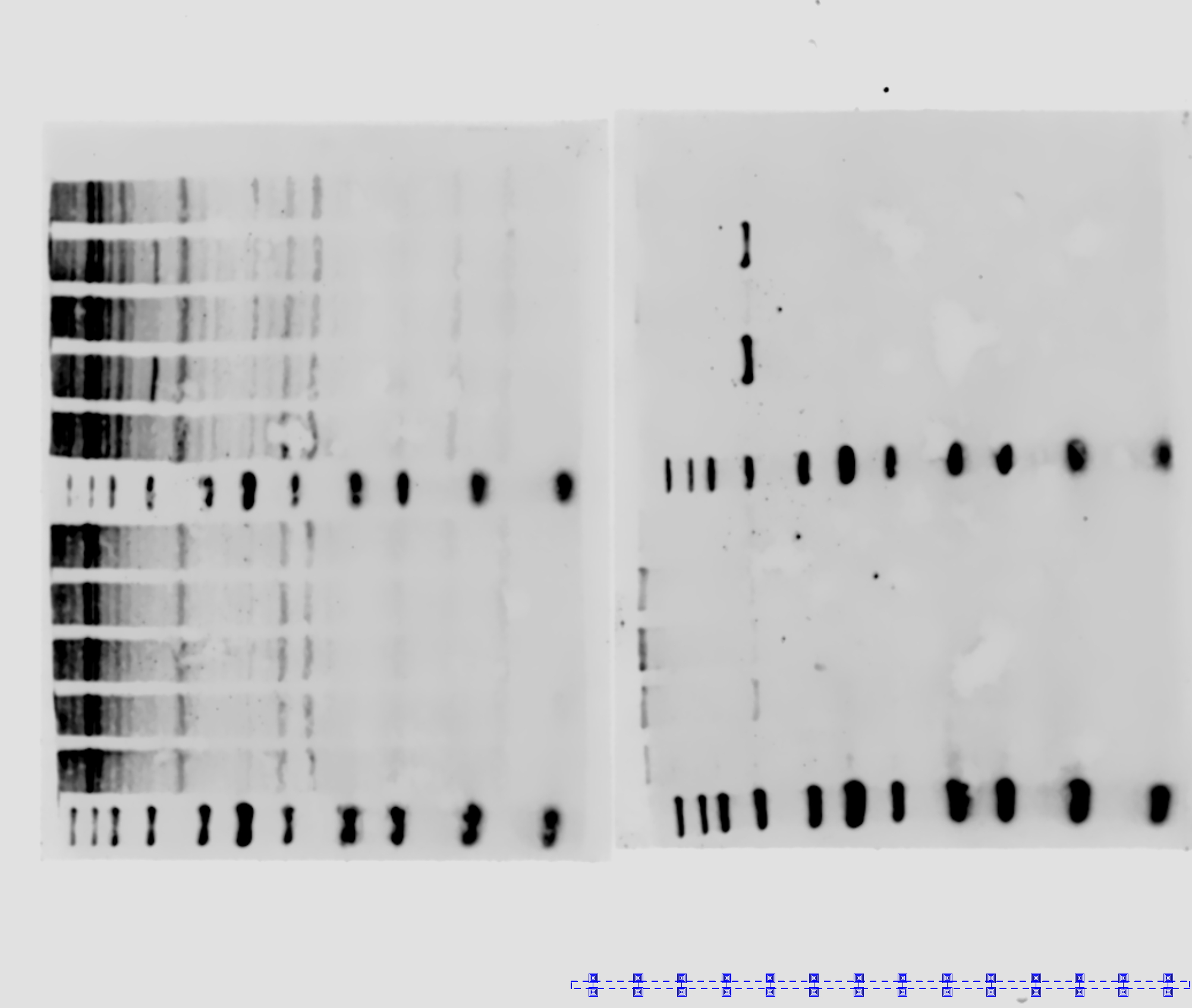

Supplement: Source data 2. [file elife-59999-data2.zip › Raw Unedited blots copy/Figure5_S2D_3892_IPtop_GTrapbottom.tif]

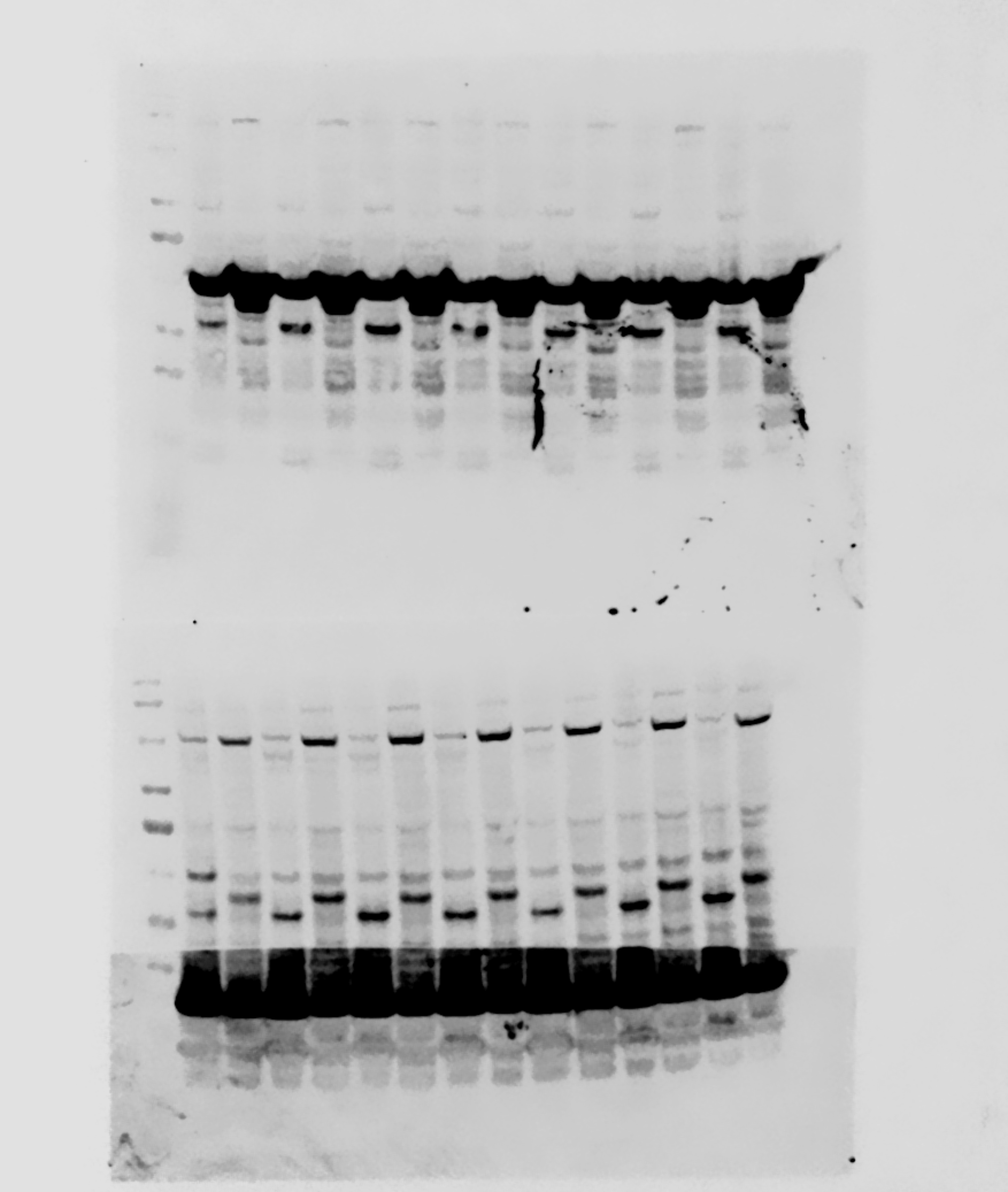

Supplement: Source data 2. [file elife-59999-data2.zip › Raw Unedited blots copy/Figure5_S2A_GFP.tif]

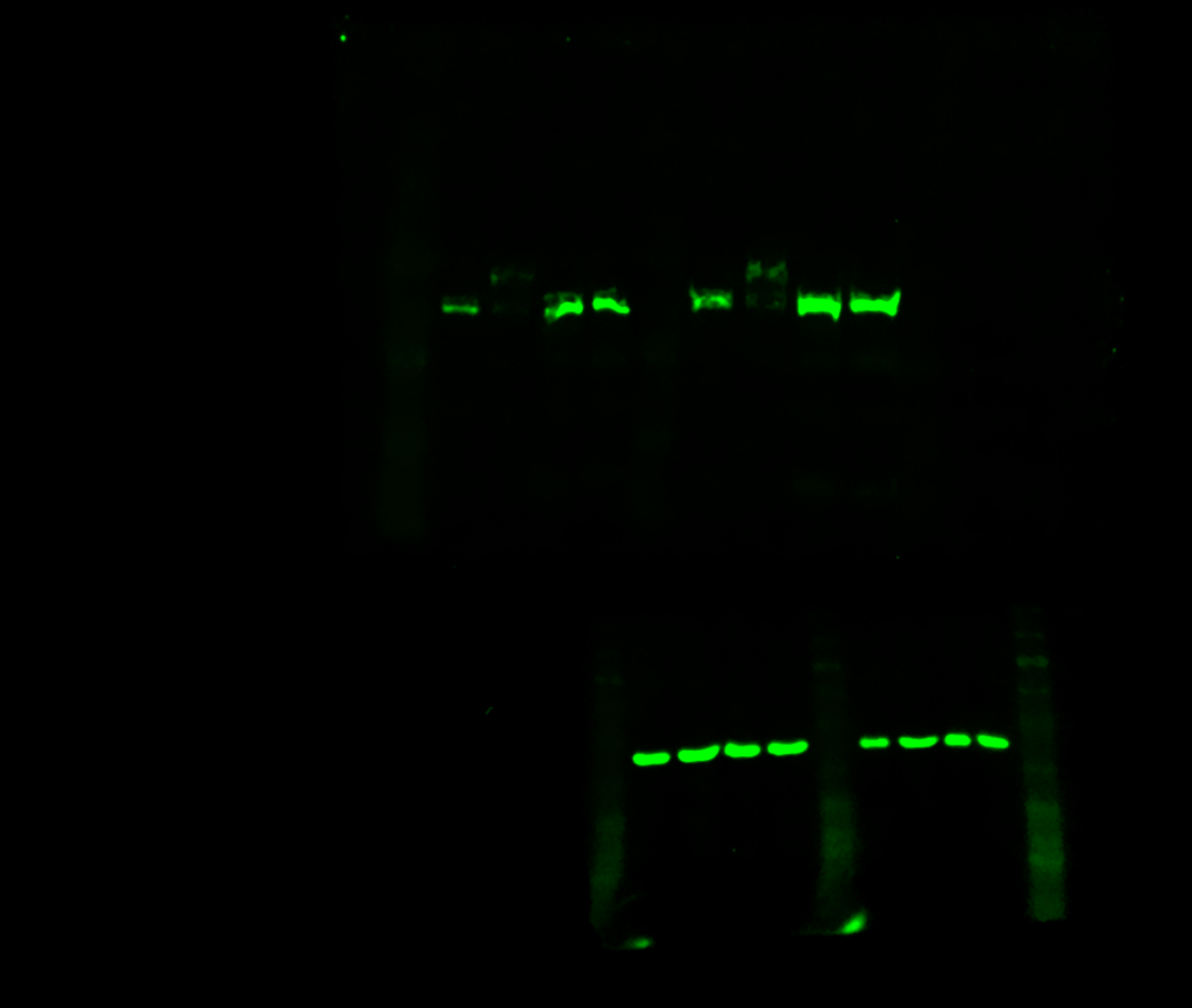

Supplement: Source data 2. [file elife-59999-data2.zip › Raw Unedited blots copy/Figure3D_GAPDH.tif]

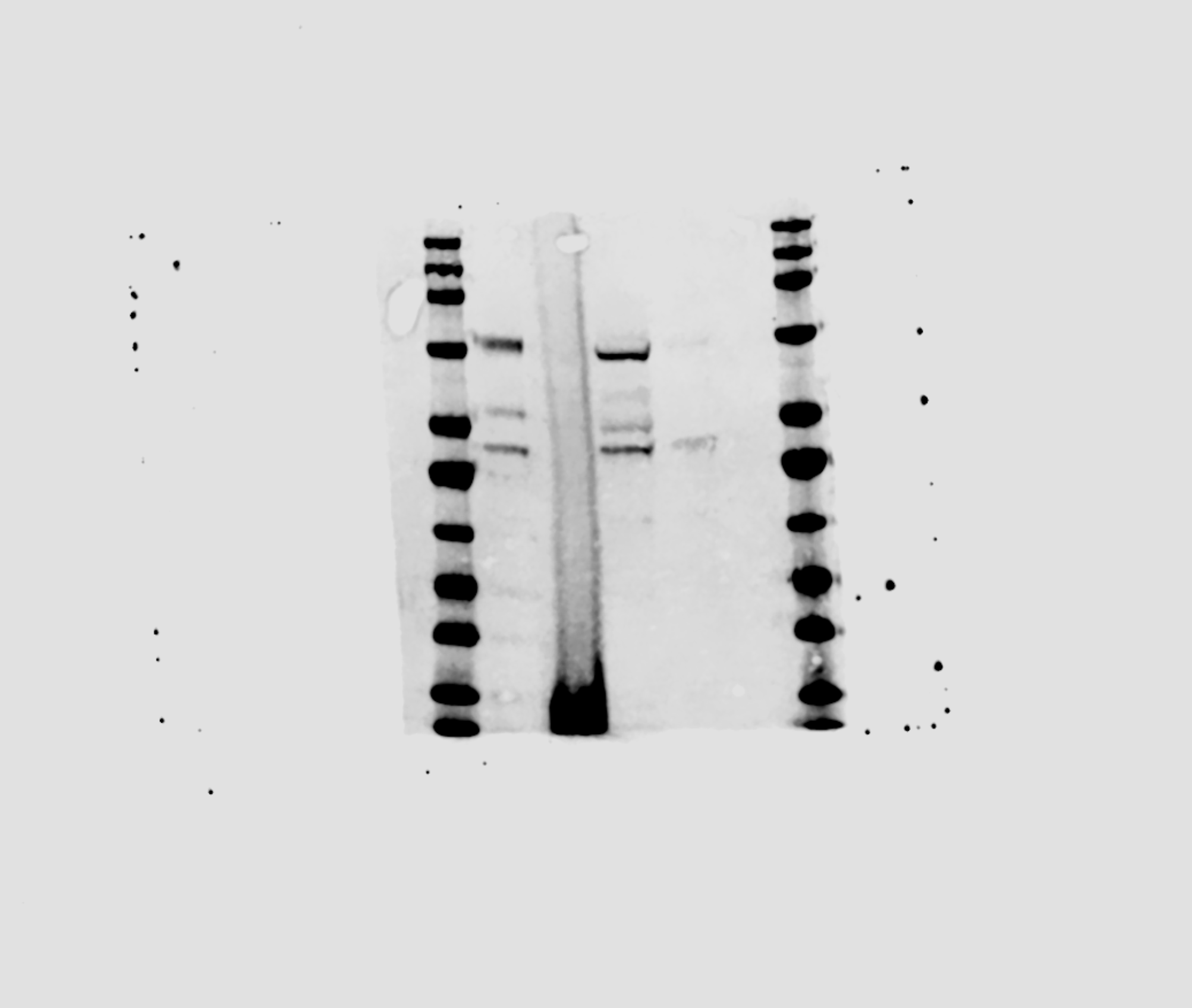

Supplement: Source data 2. [file elife-59999-data2.zip › Raw Unedited blots copy/Figure1 S2A_mCherry_markers.tif]

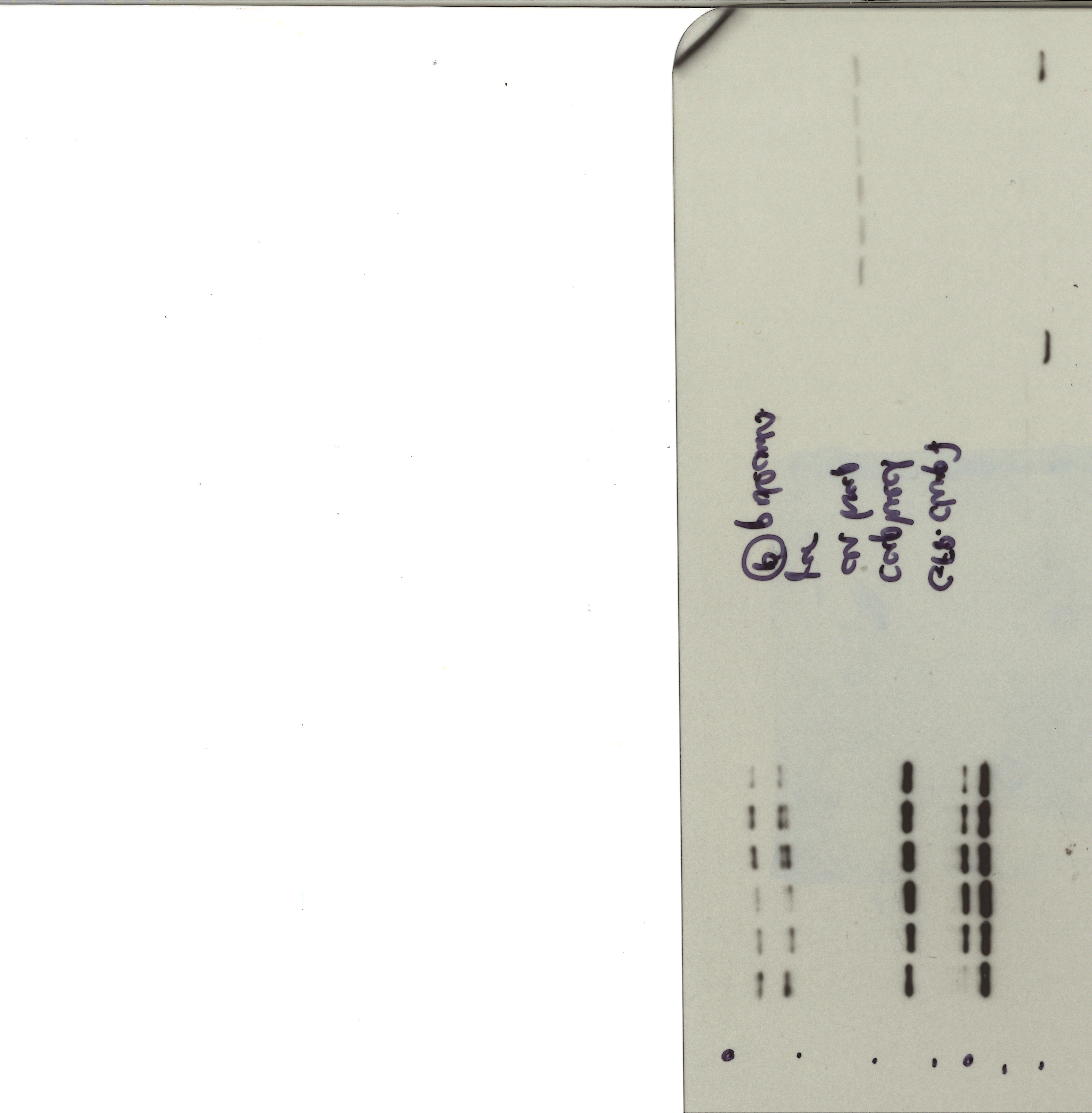

Supplement: Source data 2. [file elife-59999-data2.zip › Raw Unedited blots copy/Figure5E_GFP_Pulldown.jpg]

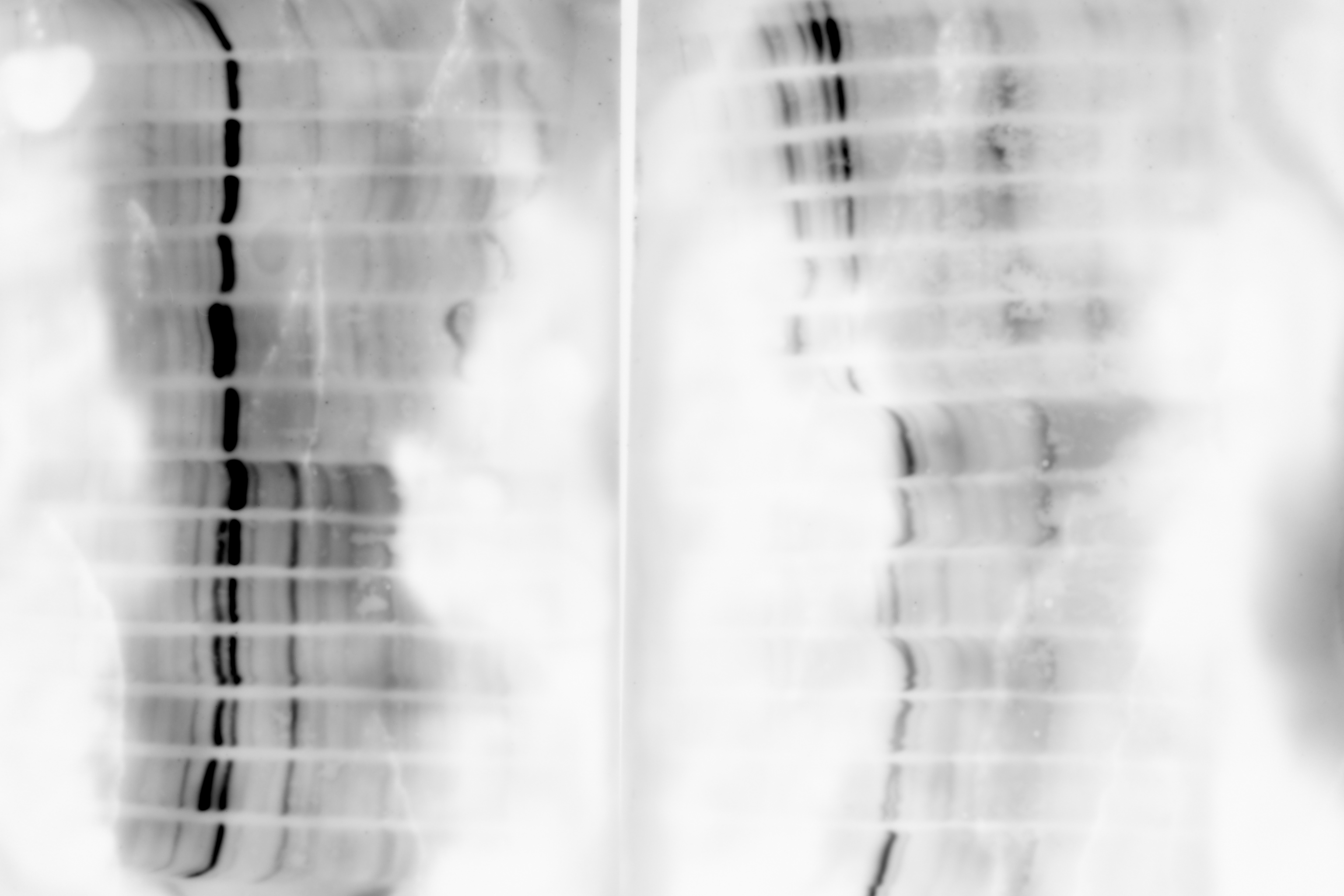

Supplement: Source data 2. [file elife-59999-data2.zip › Raw Unedited blots copy/Figure5_S2B_Phostag_chromatin_GFP.tif]

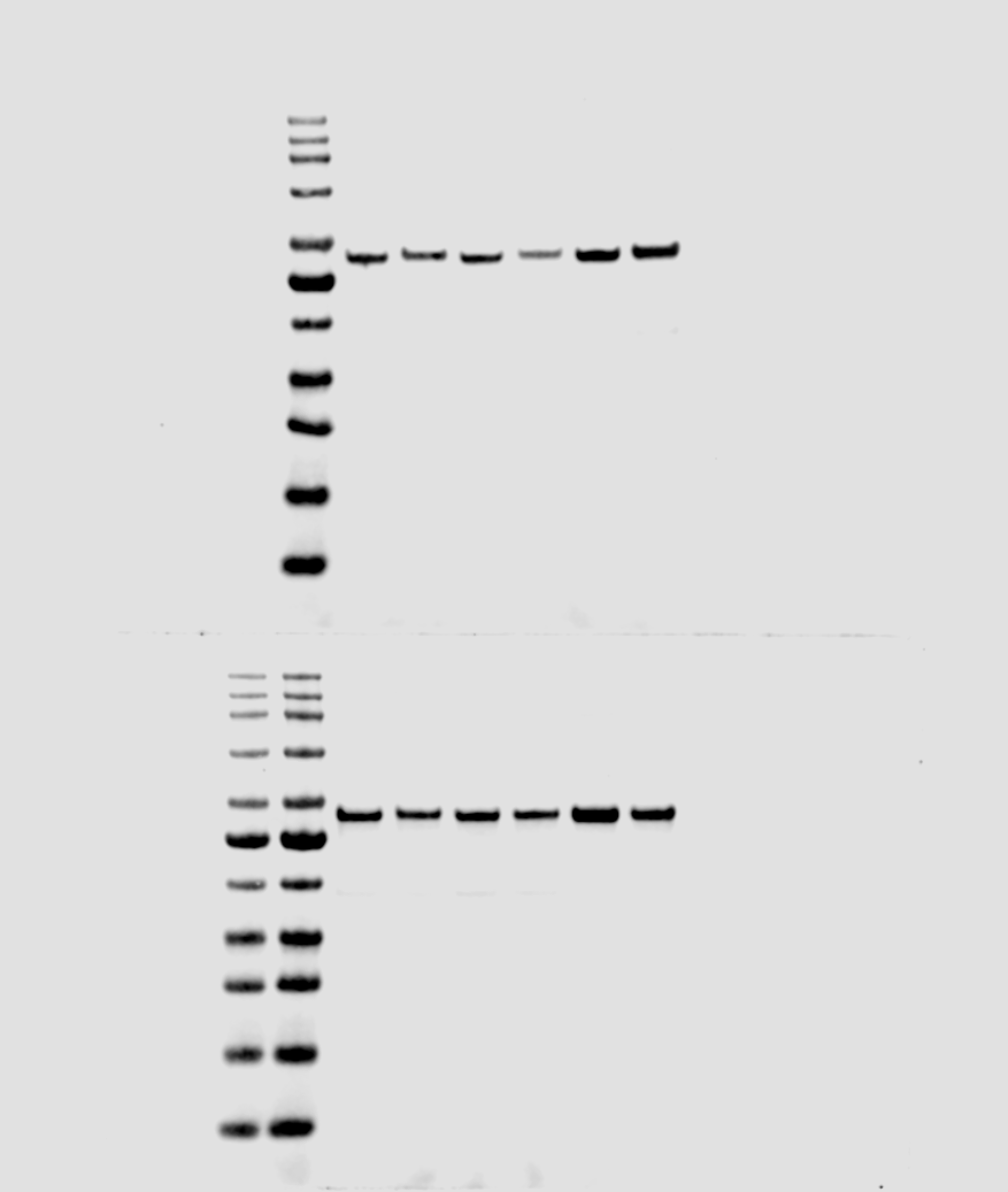

Supplement: Source data 2. [file elife-59999-data2.zip › Raw Unedited blots copy/Figure4E_Input_top_Captured_bottom_CHMP7.tif]

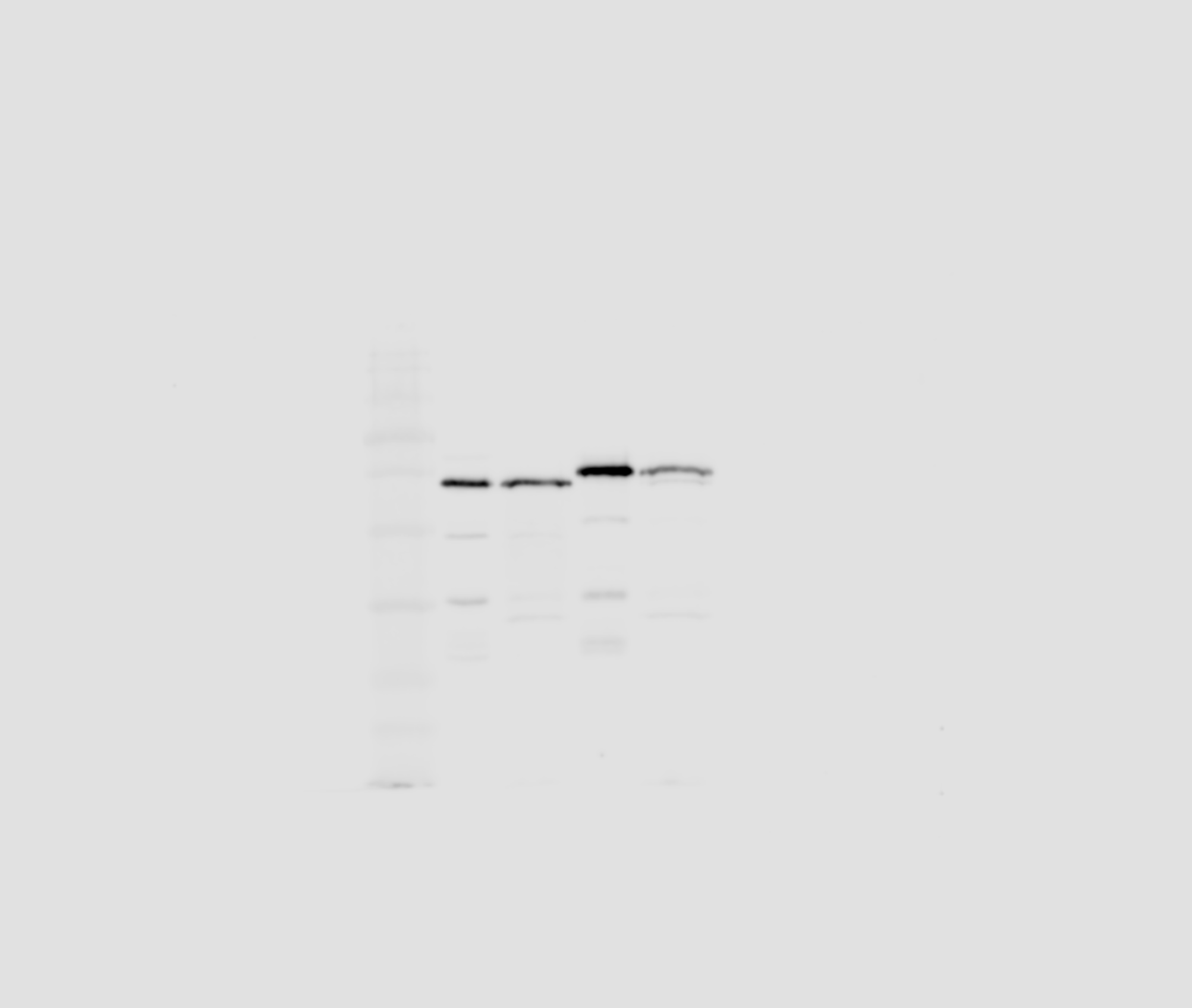

Supplement: Source data 2. [file elife-59999-data2.zip › Raw Unedited blots copy/Figure4A_CHMP7.tif]

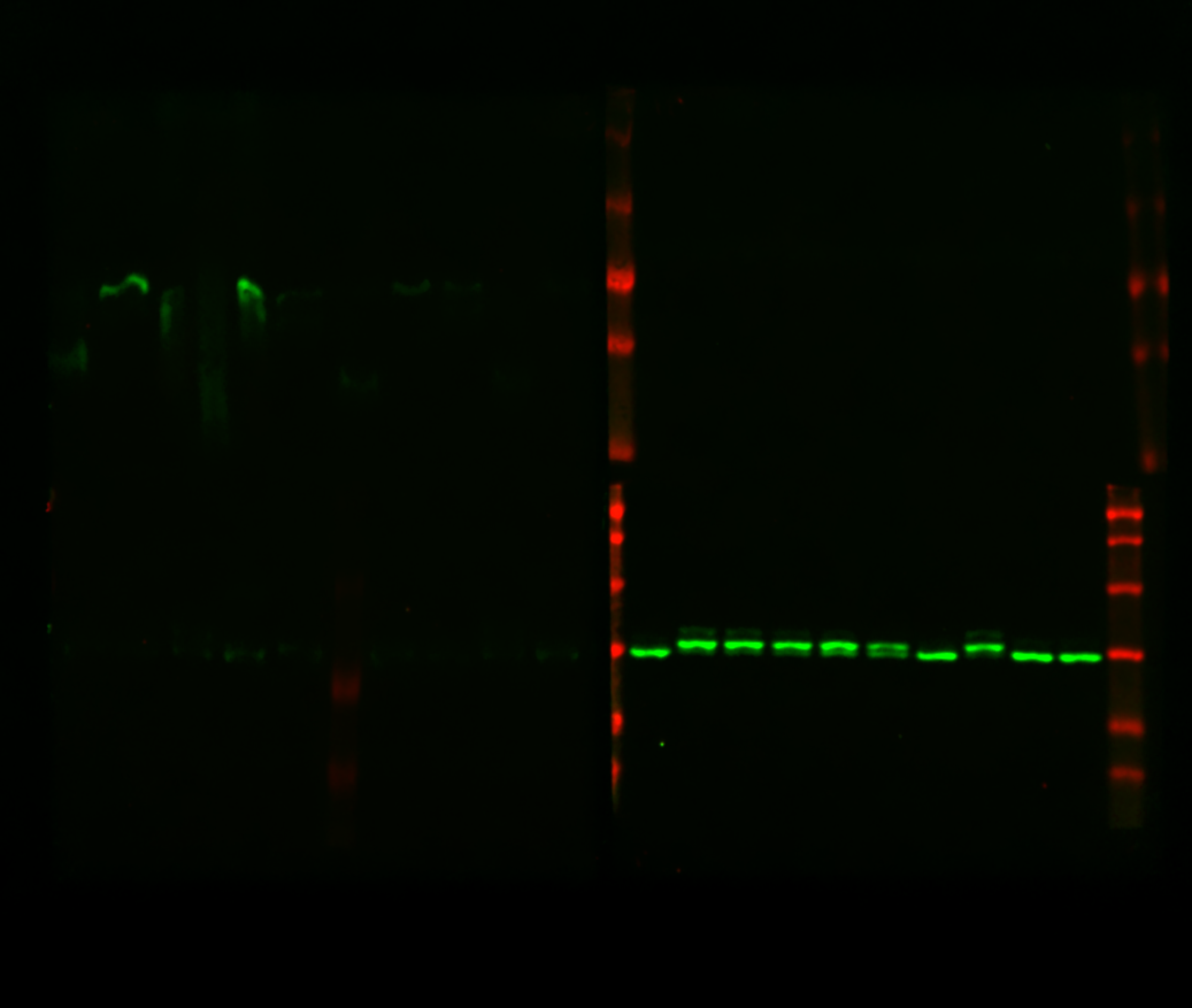

Supplement: Source data 2. [file elife-59999-data2.zip › Raw Unedited blots copy/Figure3_S1B_PhosTag_393CT_GFP.tif]

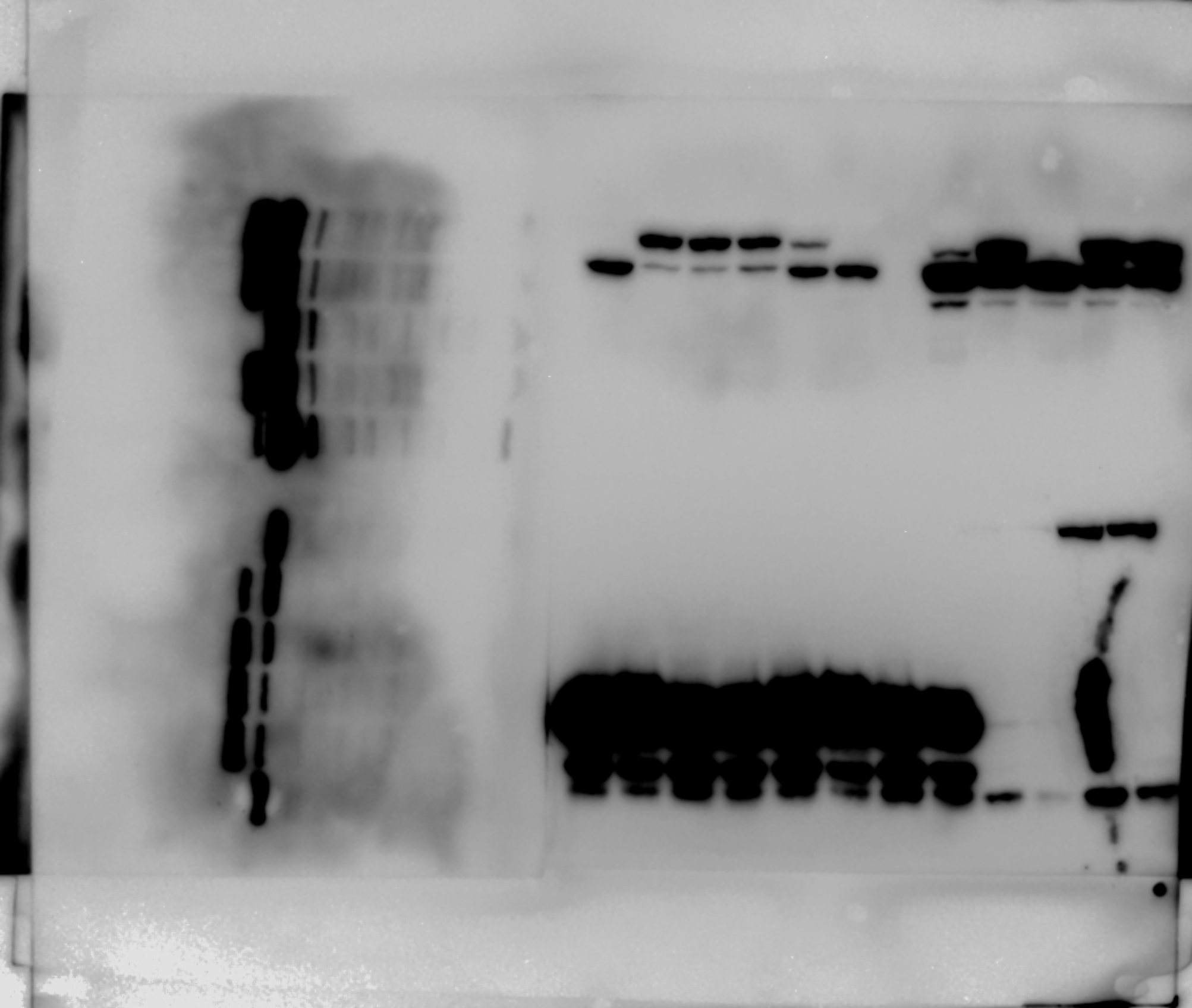

Supplement: Source data 2. [file elife-59999-data2.zip › Raw Unedited blots copy/Figure5D_Phostag_GFP.tif]

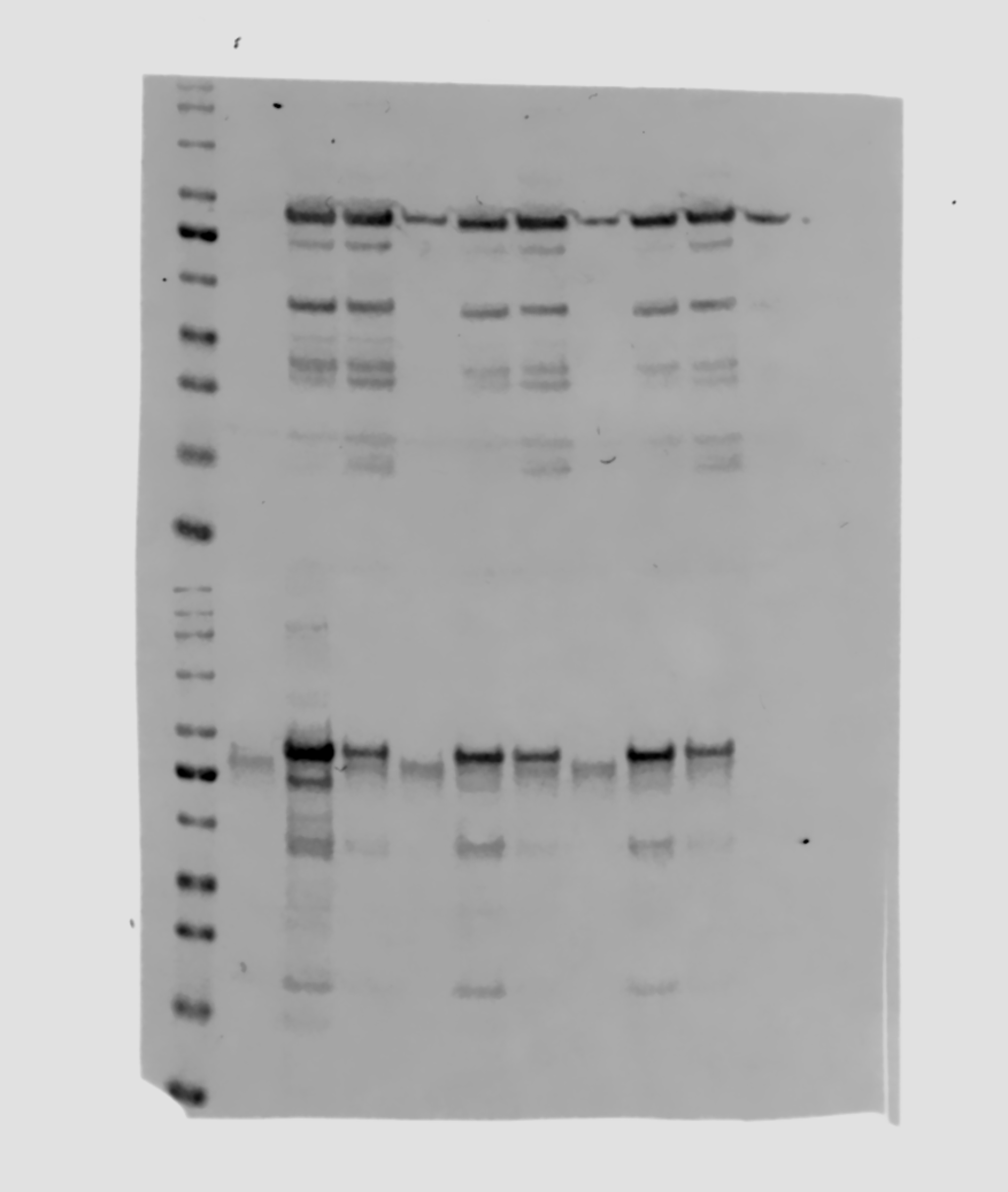

Supplement: Source data 2. [file elife-59999-data2.zip › Raw Unedited blots copy/Figure4G_inputpulldown_CHMP7]

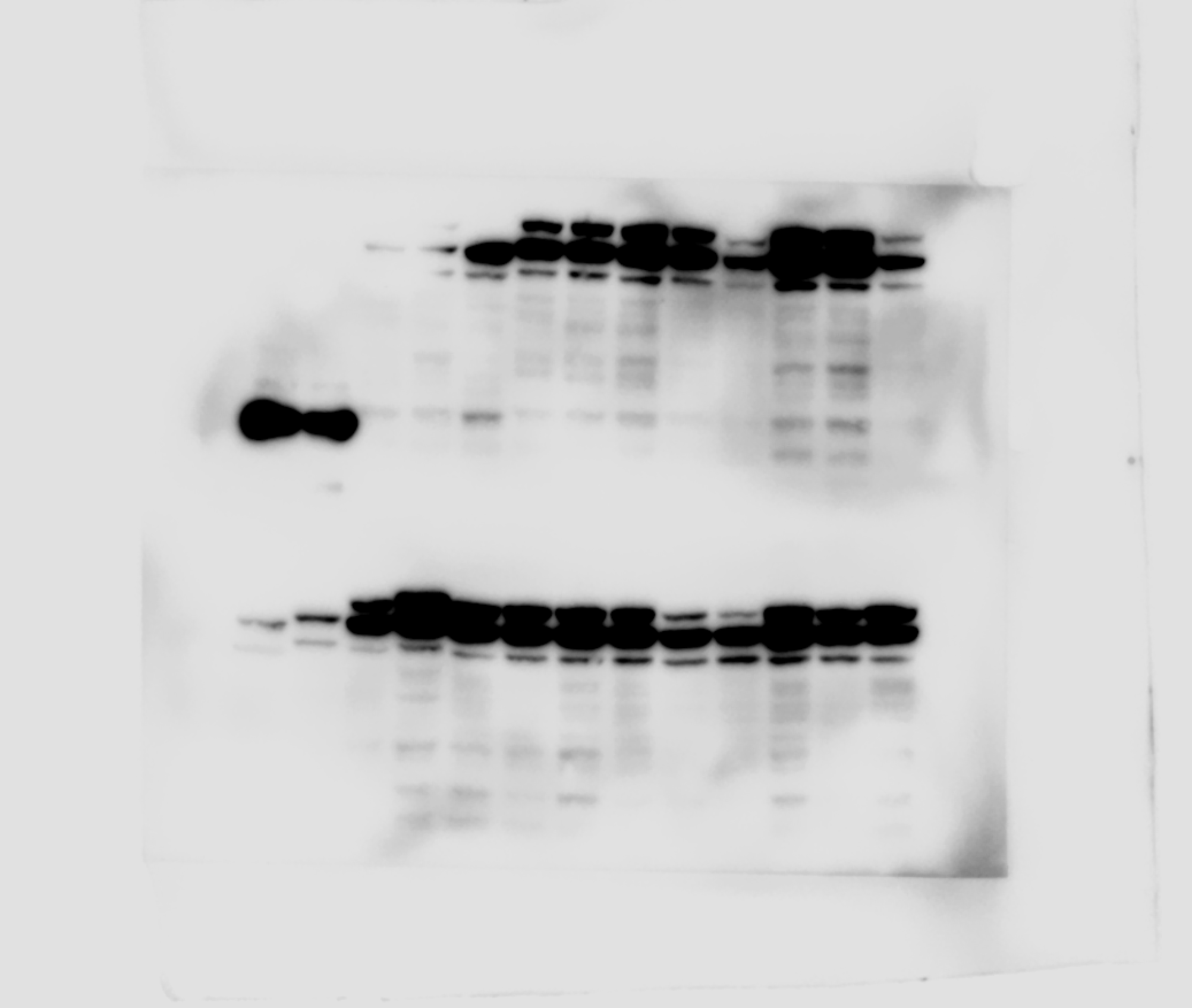

Supplement: Source data 2. [file elife-59999-data2.zip › Raw Unedited blots copy/Figure3_S1C_PhostagGFP_darkerexposure.tif]

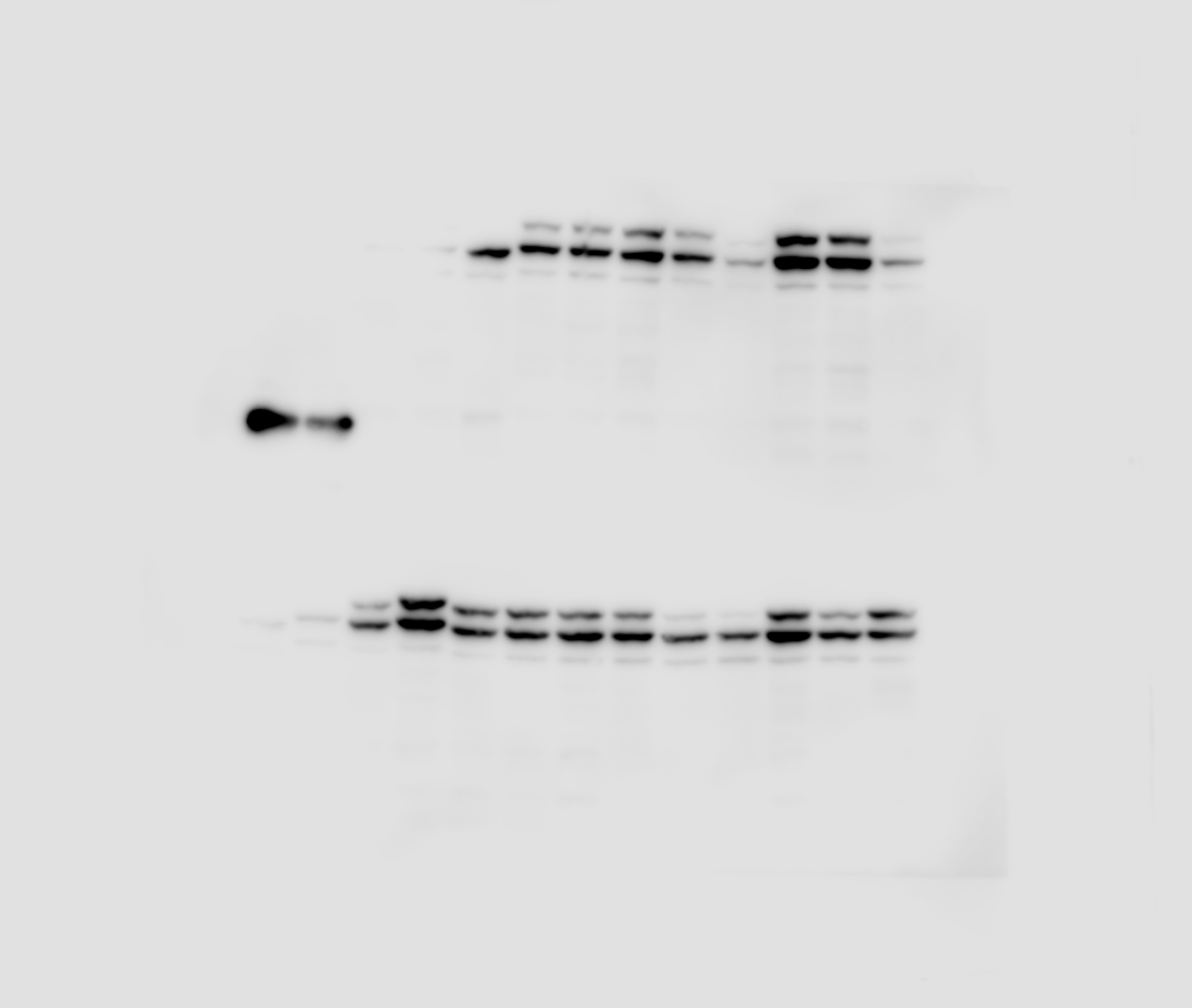

Supplement: Source data 2. [file elife-59999-data2.zip › Raw Unedited blots copy/Figure3_S1C_PhostagGFP.tif]

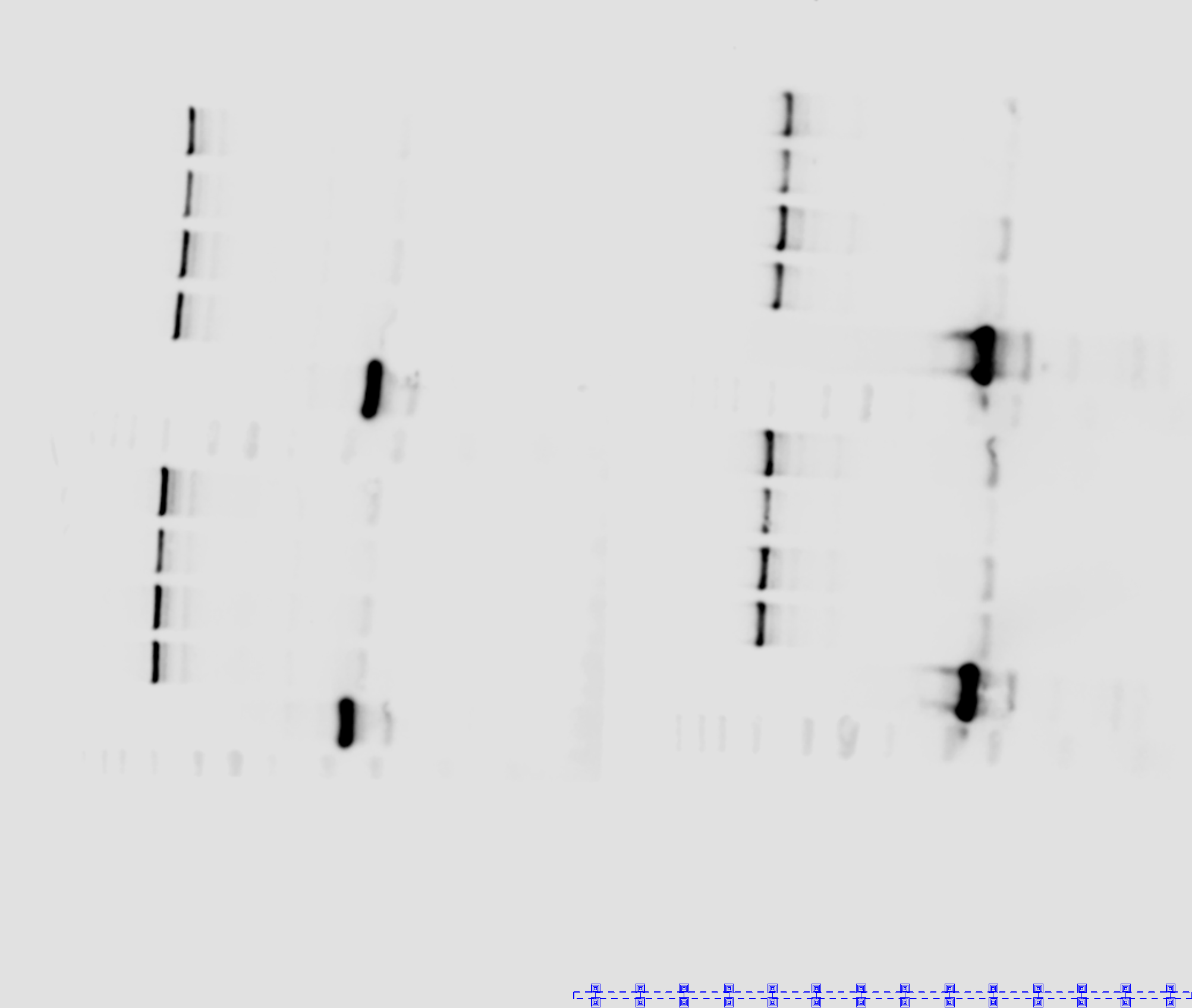

Supplement: Source data 2. [file elife-59999-data2.zip › Raw Unedited blots copy/Figure5_S2D_3891_IPtop_GTrapbottom_GFP.tif]

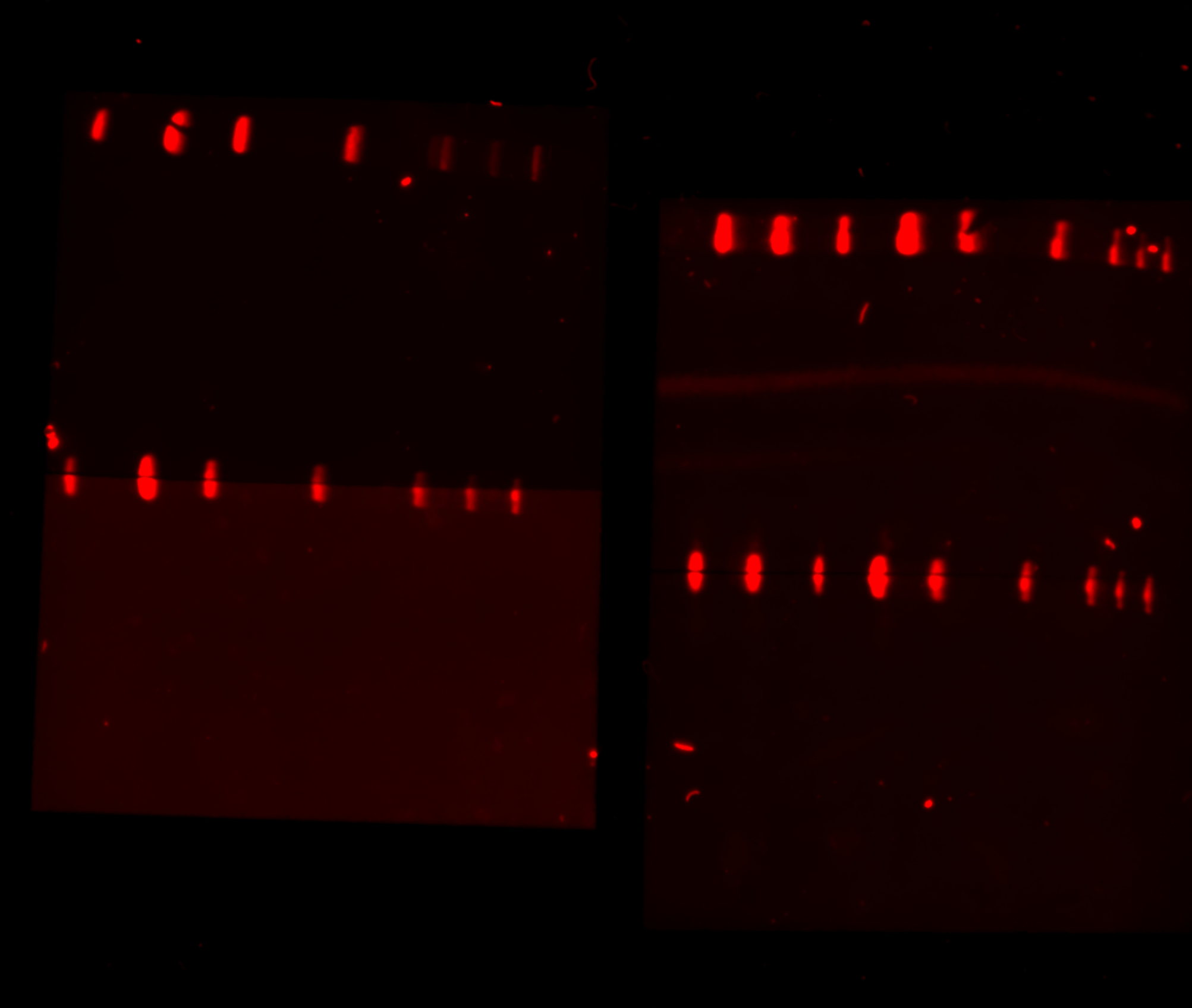

Supplement: Source data 2. [file elife-59999-data2.zip › Raw Unedited blots copy/Figure1B_IST1_HSP90_markers.tif]

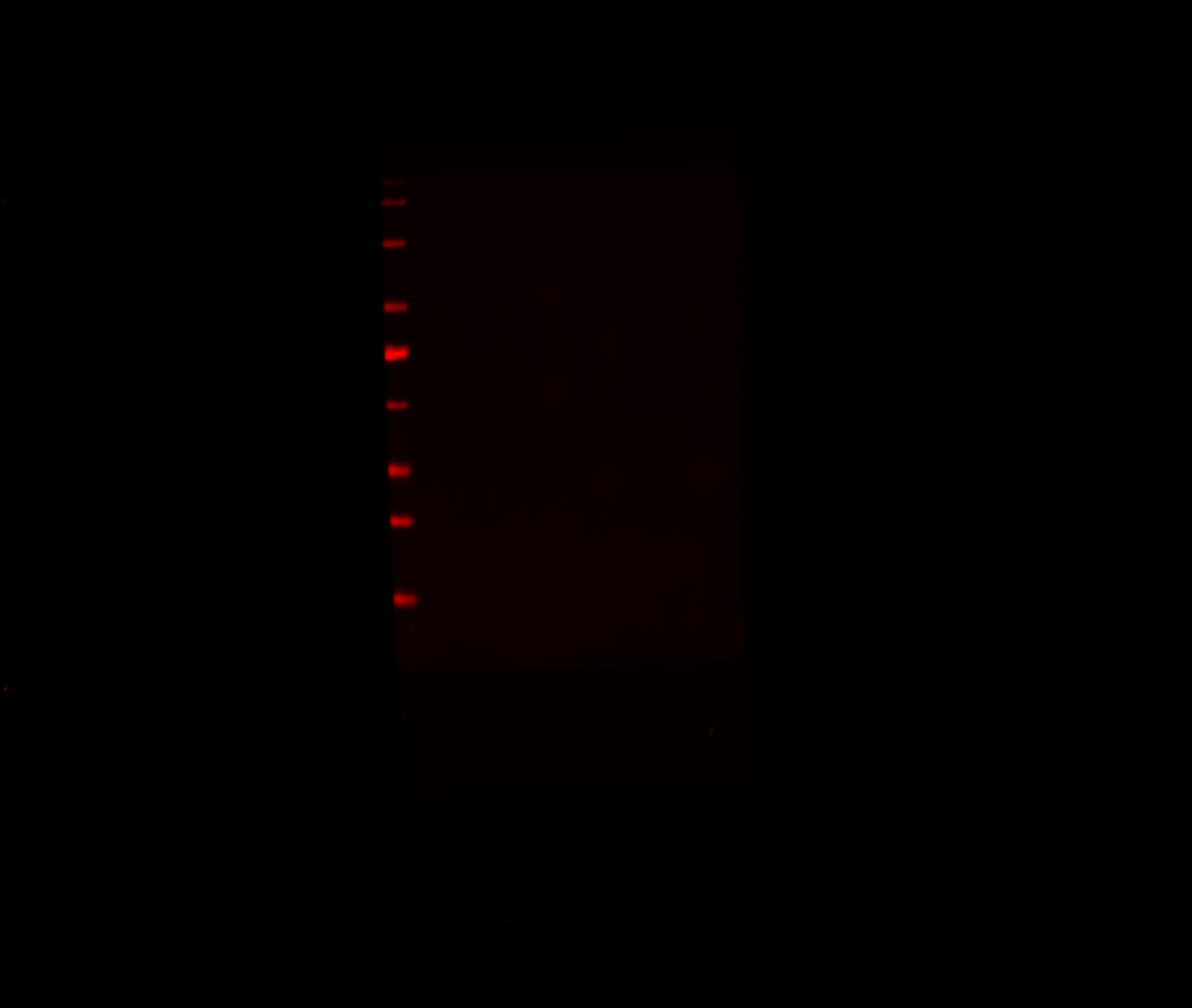

Supplement: Source data 2. [file elife-59999-data2.zip › Raw Unedited blots copy/Figure1B_LEM2markers.tif]

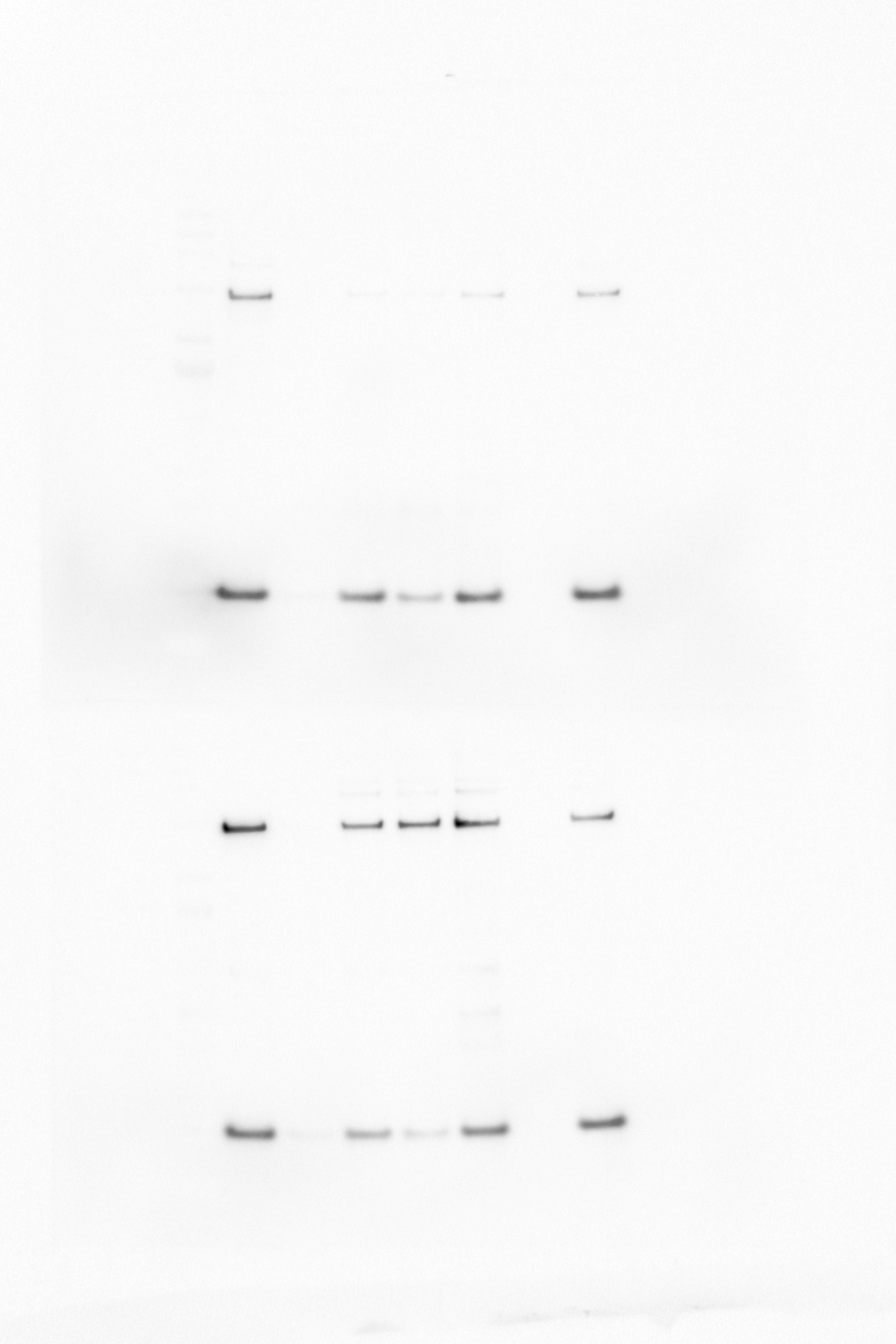

Supplement: Source data 2. [file elife-59999-data2.zip › Raw Unedited blots copy/Figure4_S1C_HAblots_bottom.tif]

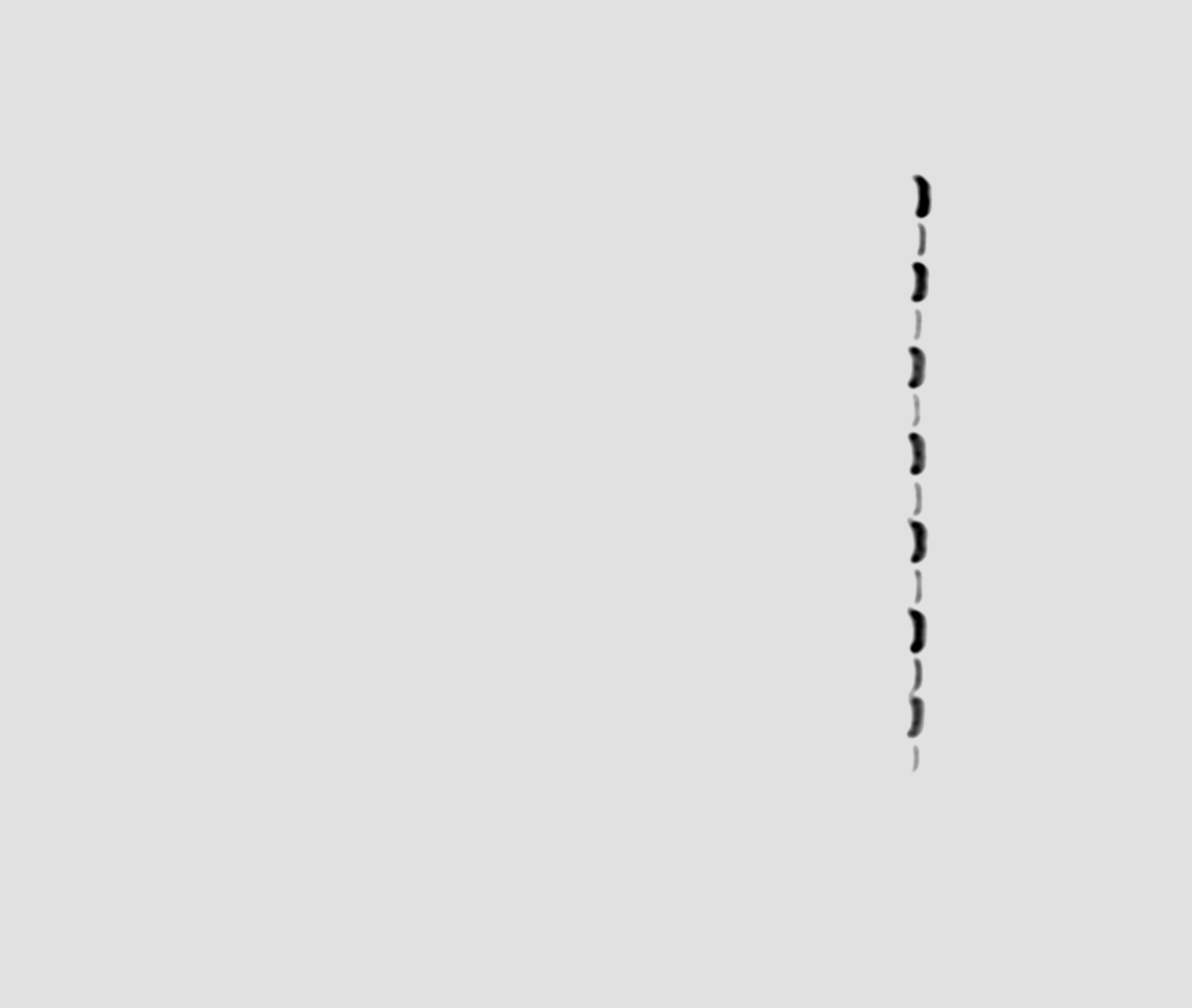

Supplement: Source data 2. [file elife-59999-data2.zip › Raw Unedited blots copy/Figure5_S2A_G3PDH.tif]

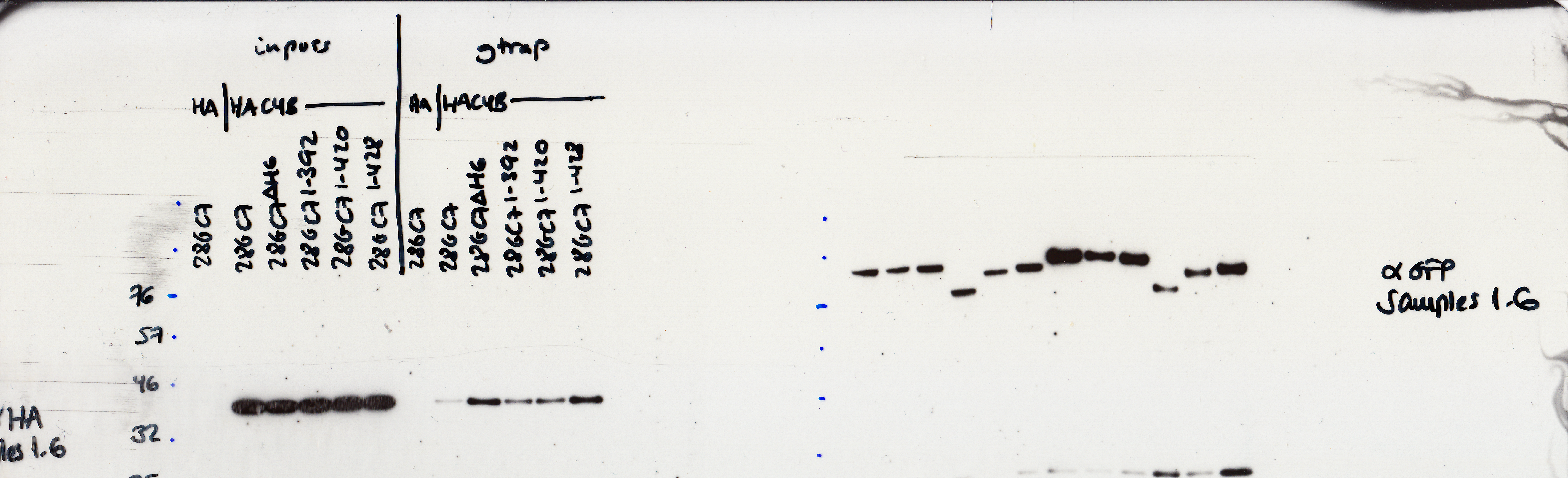

Supplement: Source data 2. [file elife-59999-data2.zip › Raw Unedited blots copy/Figure2_S3C_inputs_pulldown_HA_GFP.tif]

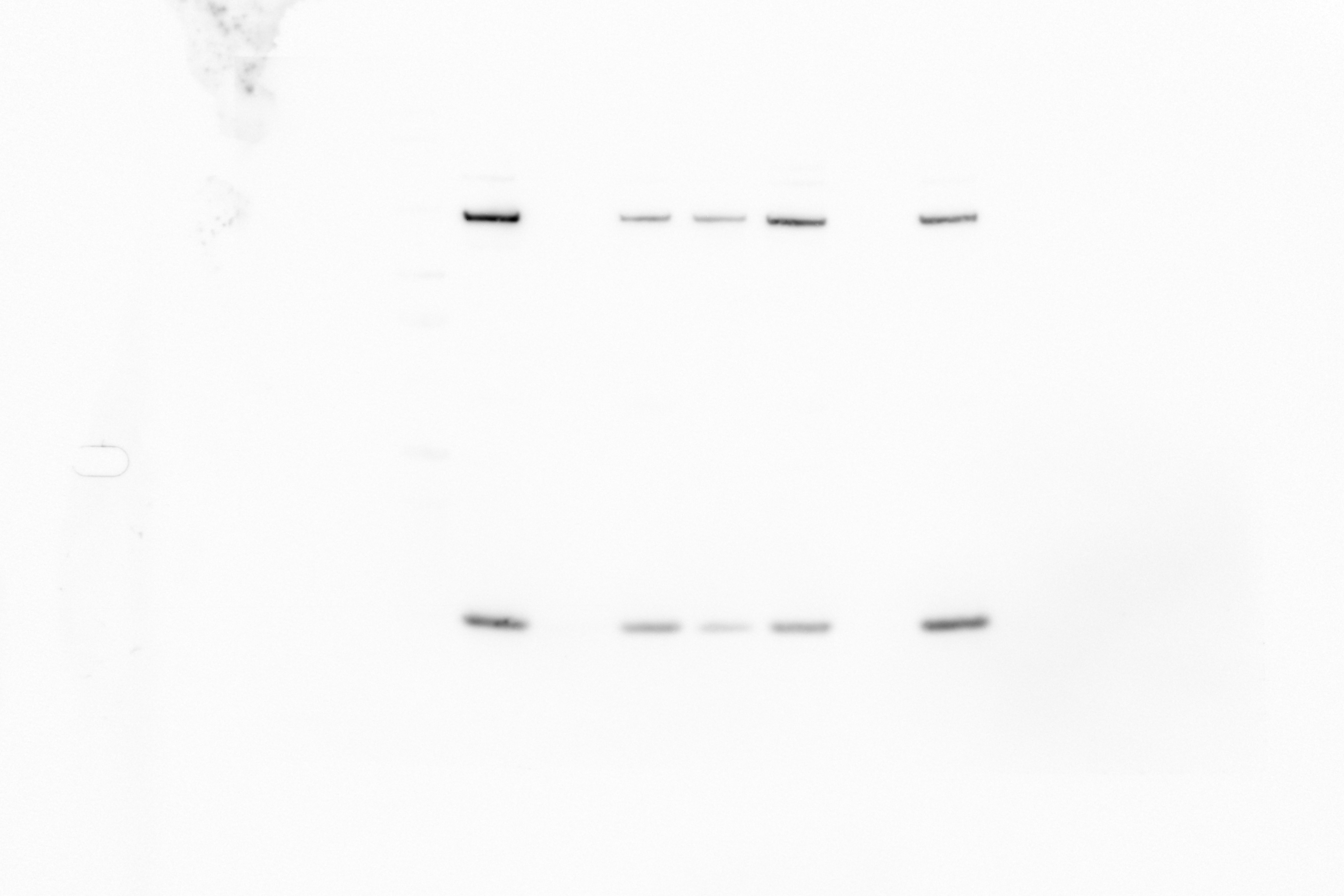

Supplement: Source data 2. [file elife-59999-data2.zip › Raw Unedited blots copy/Figure4_S1A_LEM2_HA.tif]

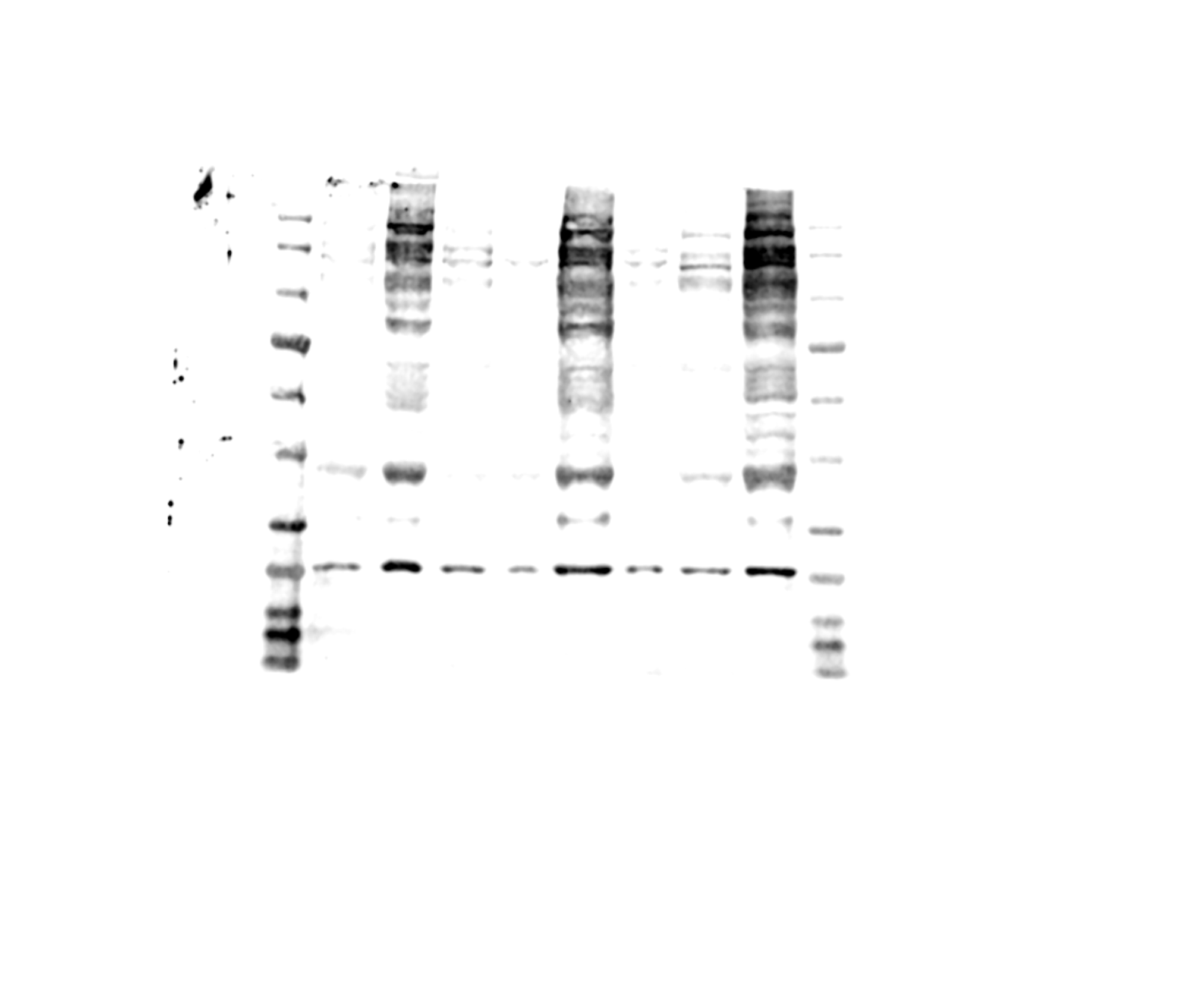

Supplement: Source data 2. [file elife-59999-data2.zip › Raw Unedited blots copy/Figure3C_KH-pS-P_inputs.tiff]

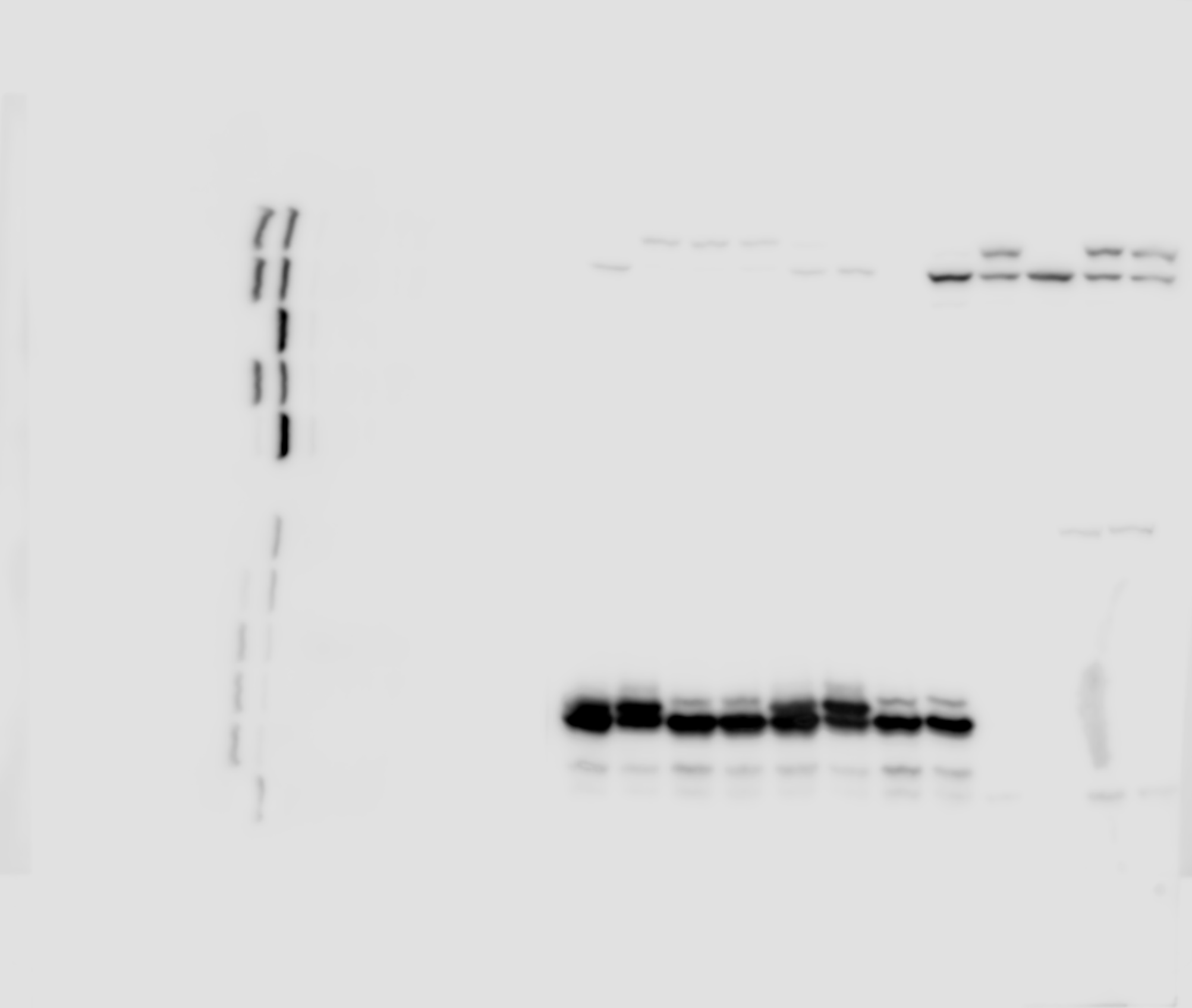

Supplement: Source data 2. [file elife-59999-data2.zip › Raw Unedited blots copy/Figure3_S1D_GFPC7NTmuts_PhostagGFP.tif]

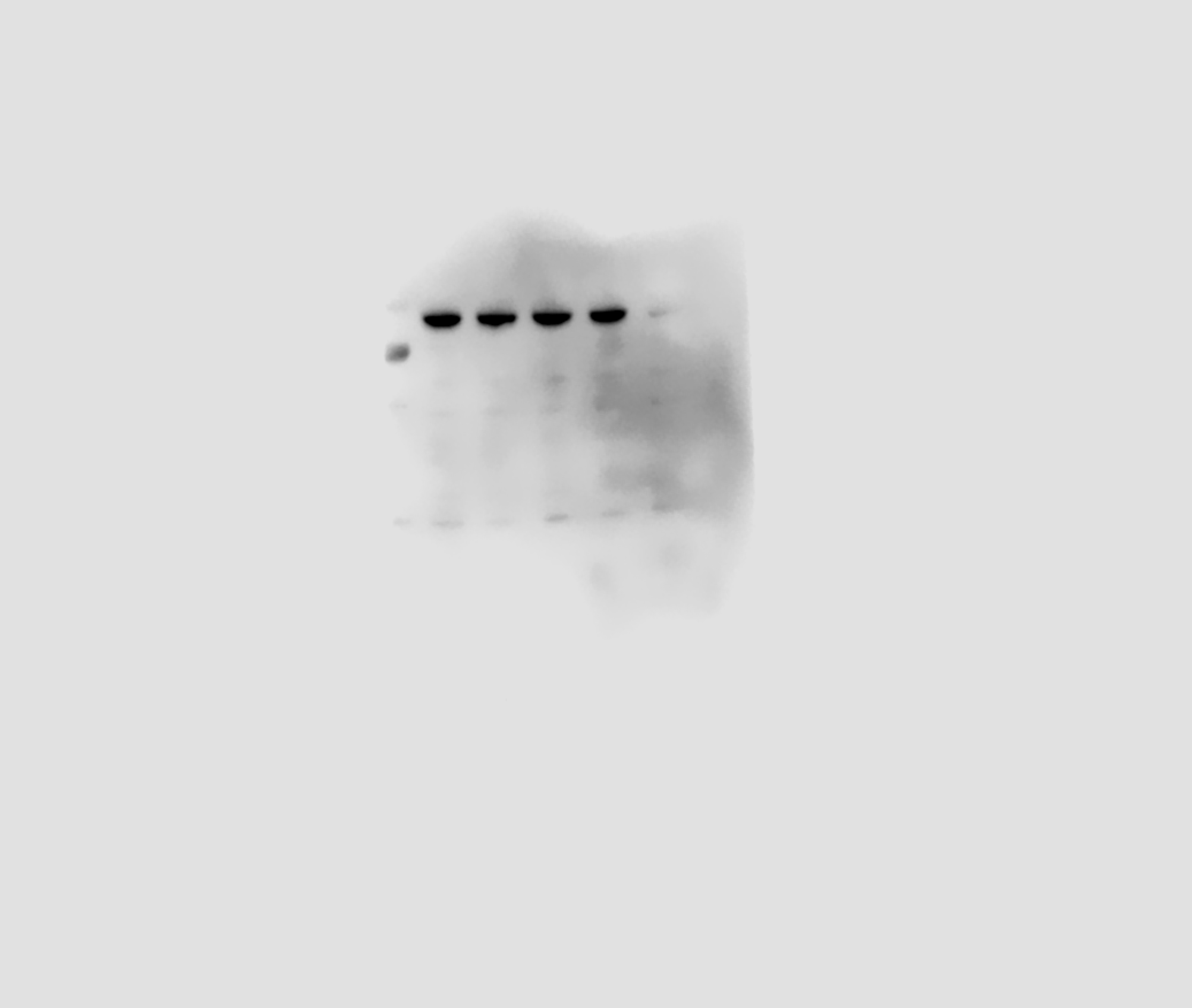

Supplement: Source data 2. [file elife-59999-data2.zip › Raw Unedited blots copy/Figure1B_LEM2.tif]

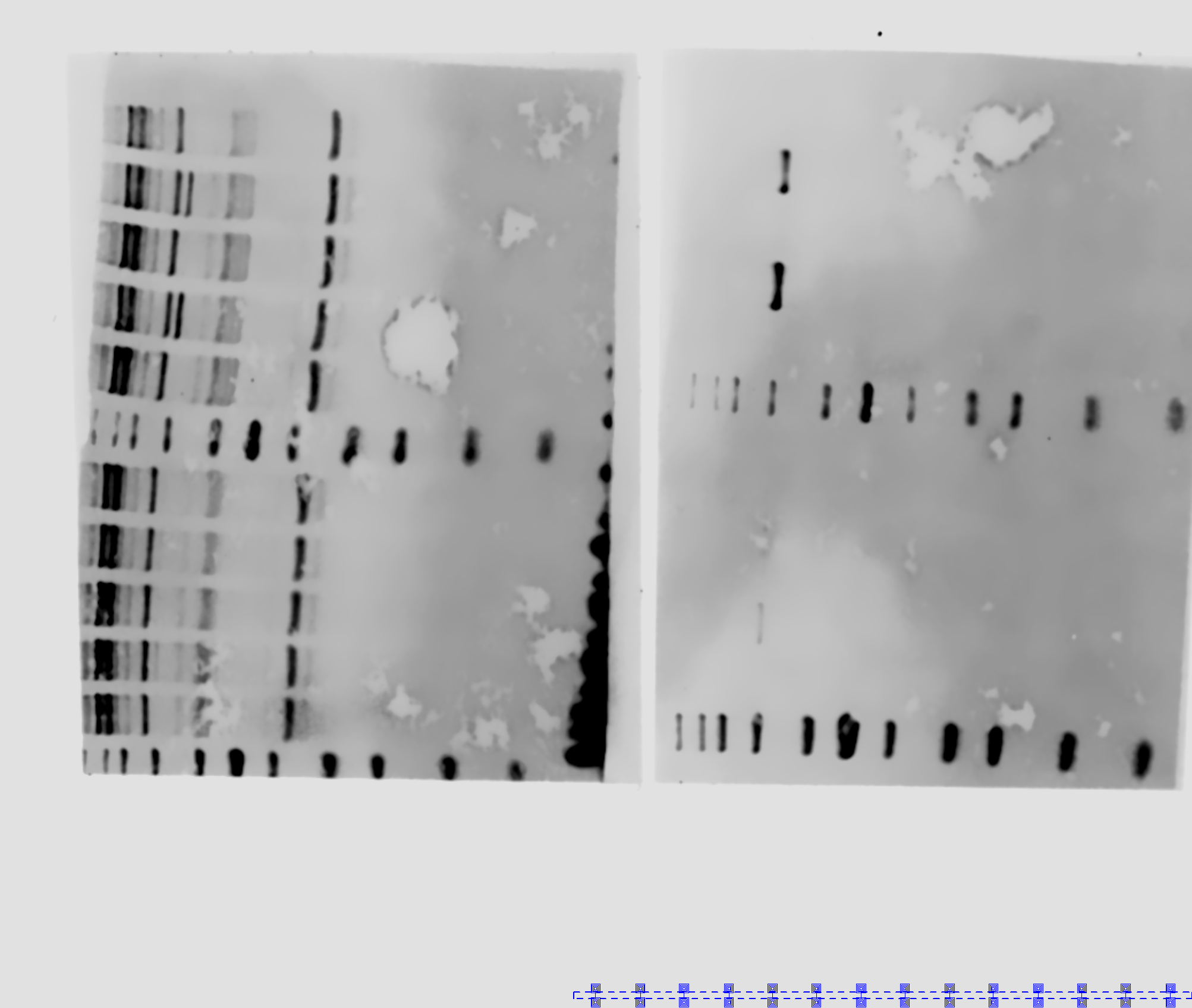

Supplement: Source data 2. [file elife-59999-data2.zip › Raw Unedited blots copy/Figure5_S2D_3891_IPtop_GTrapbottom.tif]

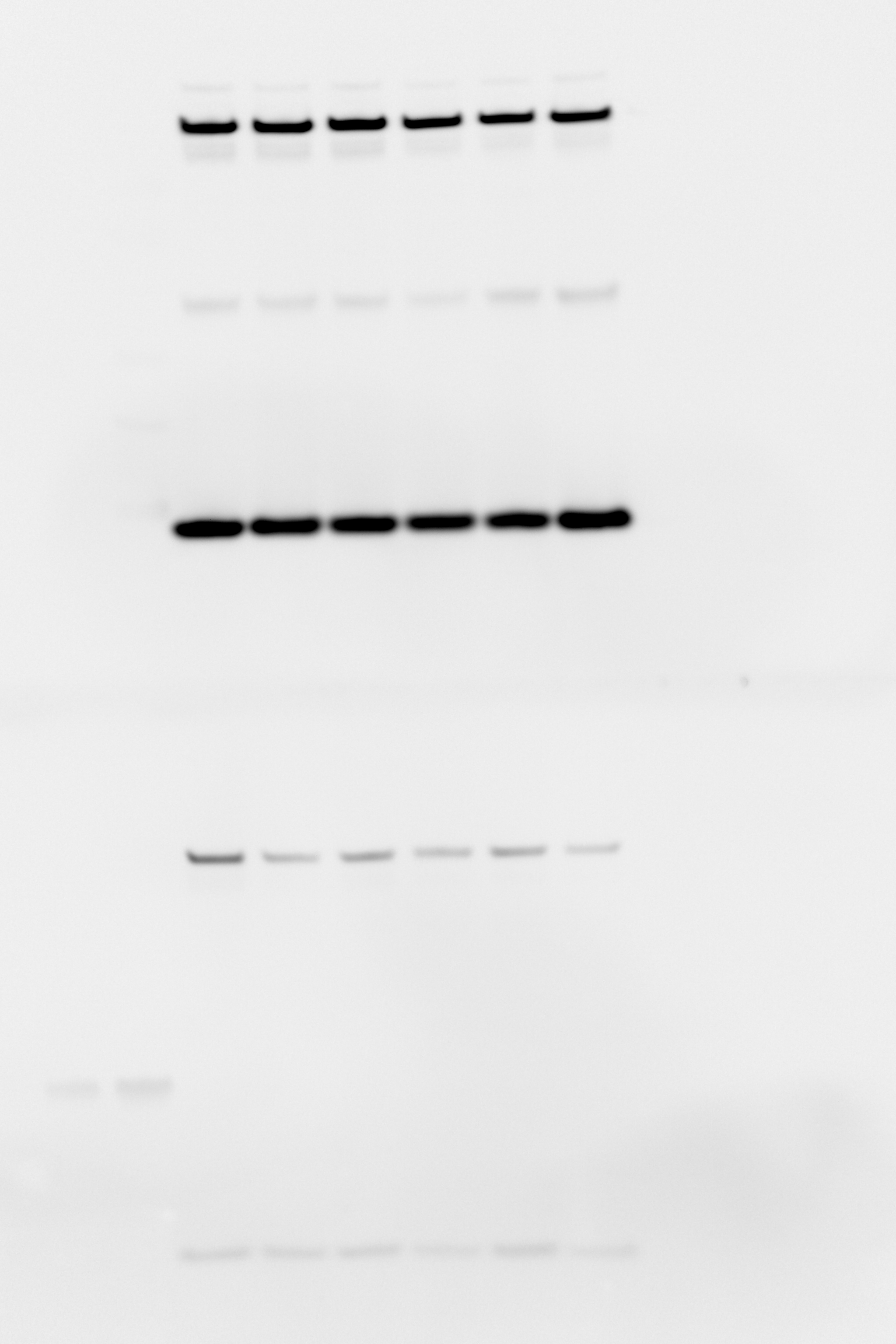

Supplement: Source data 2. [file elife-59999-data2.zip › Raw Unedited blots copy/Figure4E_Input_top_Captured_bottom_HA.tif]

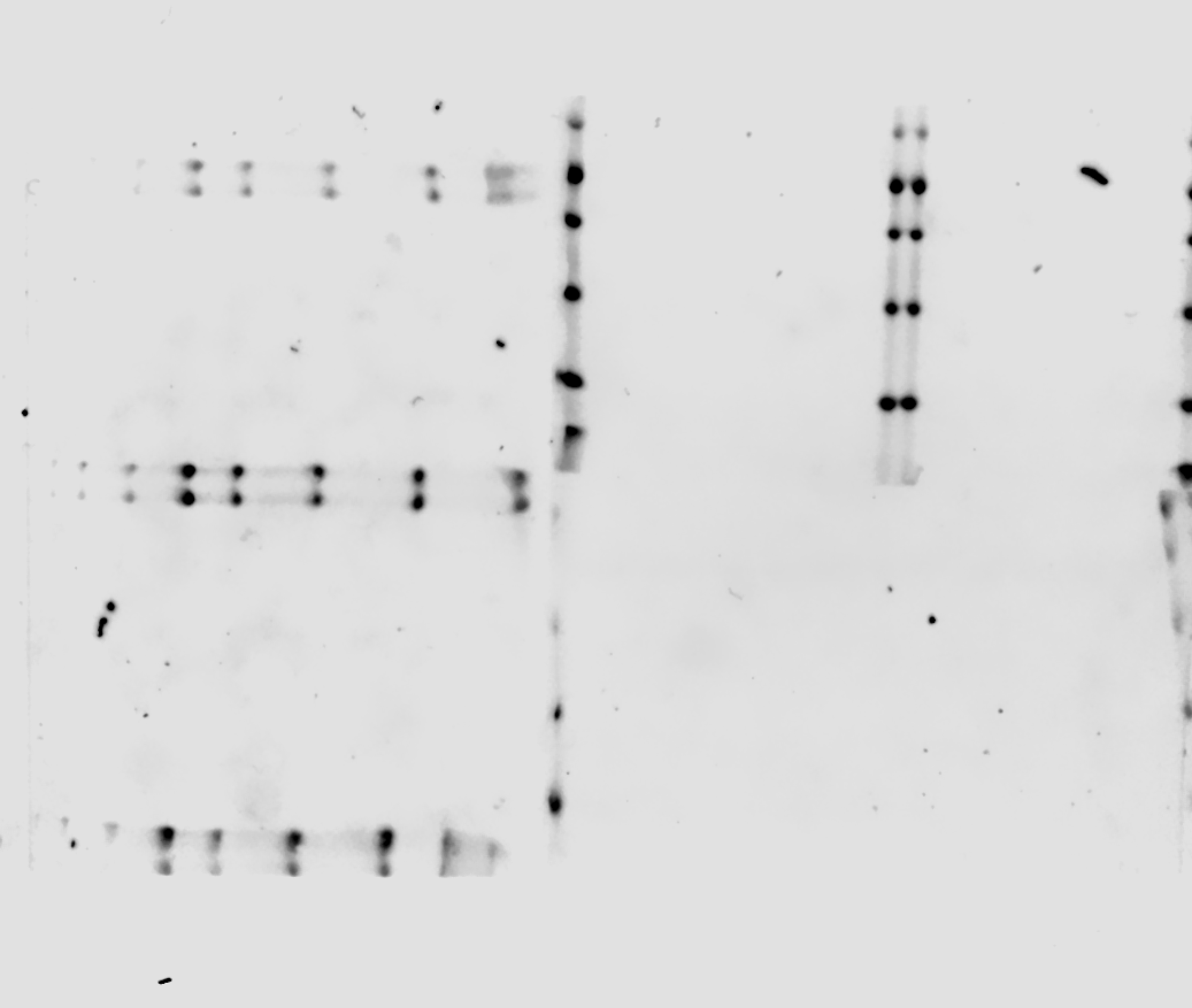

Supplement: Source data 2. [file elife-59999-data2.zip › Raw Unedited blots copy/FIgure5D_Phostag_GFP_markers.tif]
