# Supplementary figures and images for: CDK1 controls CHMP7-dependent nuclear envelope reformation (part 2 of 2)
Source: eLife. 2021 Jul 21;10:e59999. doi: 10.7554/eLife.59999 (PMC8324300; doi:10.7554/eLife.59999)

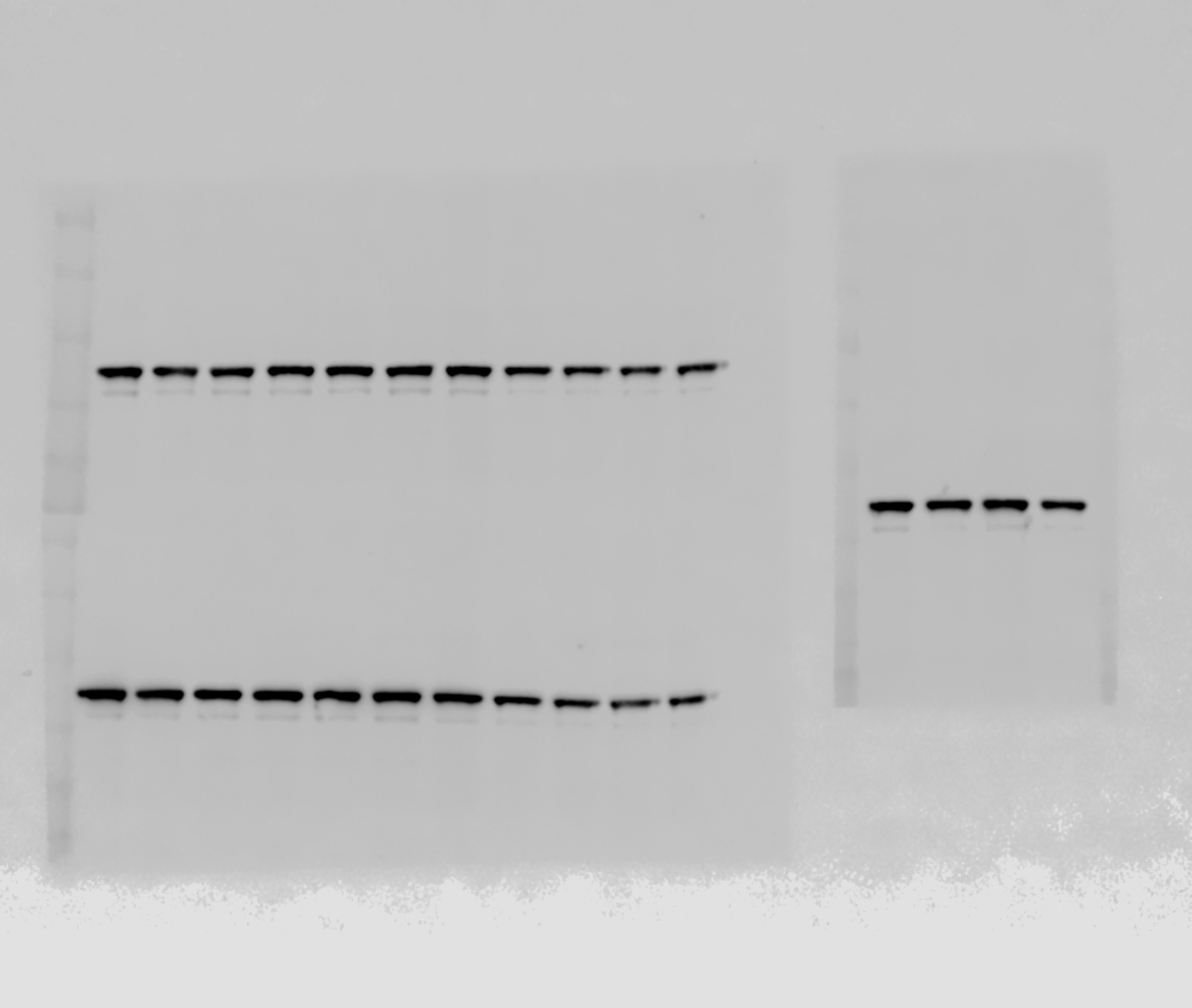

Supplement: Source data 2. [file elife-59999-data2.zip › Raw Unedited blots copy/Figure3_S1D_and_5D_GAPDH.tif]

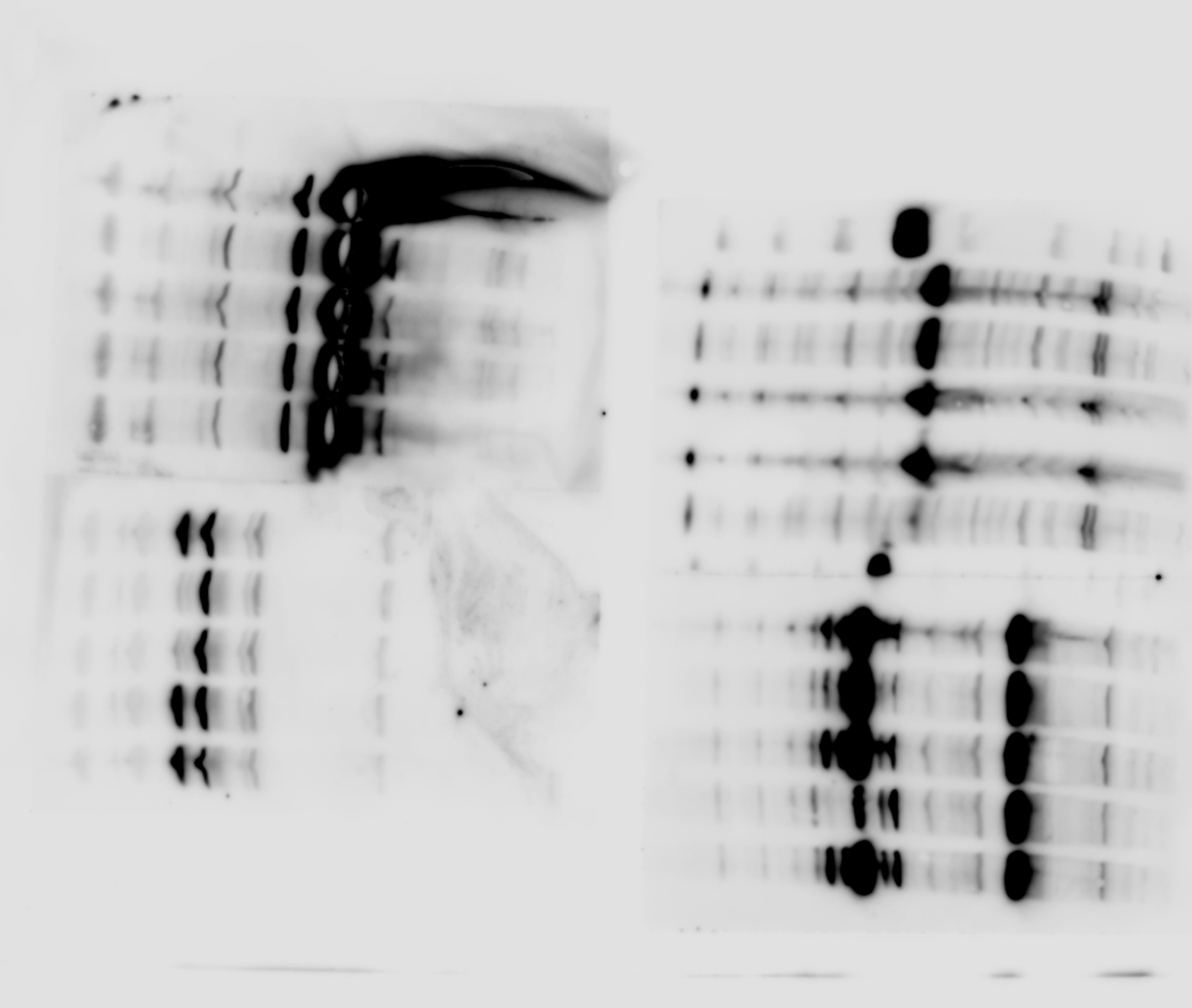

Supplement: Source data 2. [file elife-59999-data2.zip › Raw Unedited blots copy/Figure1B_CHMP7.tif]

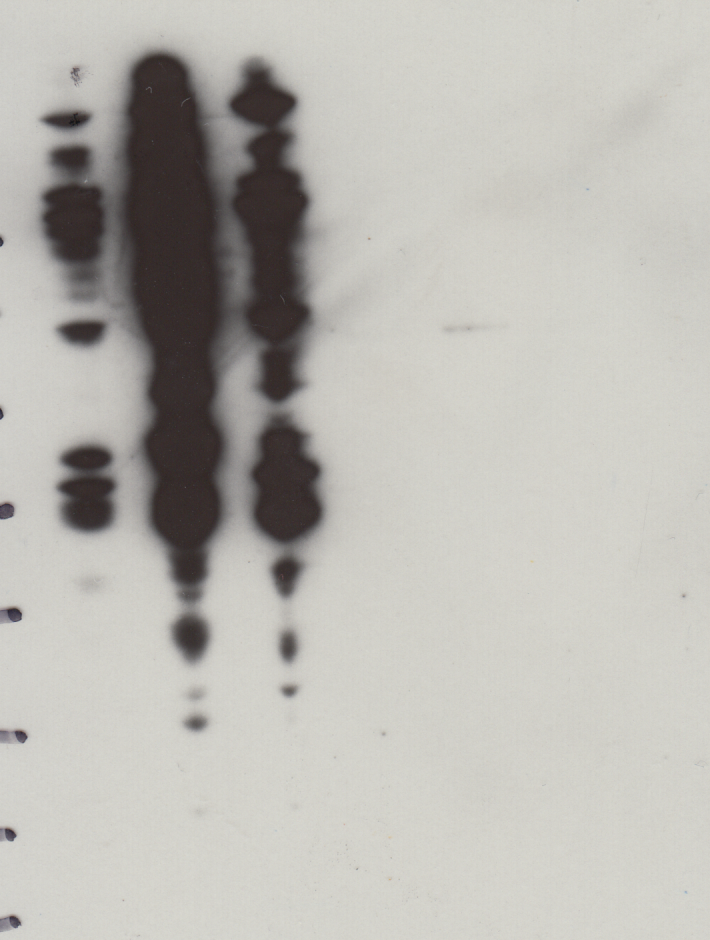

Supplement: Source data 2. [file elife-59999-data2.zip › Raw Unedited blots copy/Figure3C_pS-P-X-KR_PD.tiff]

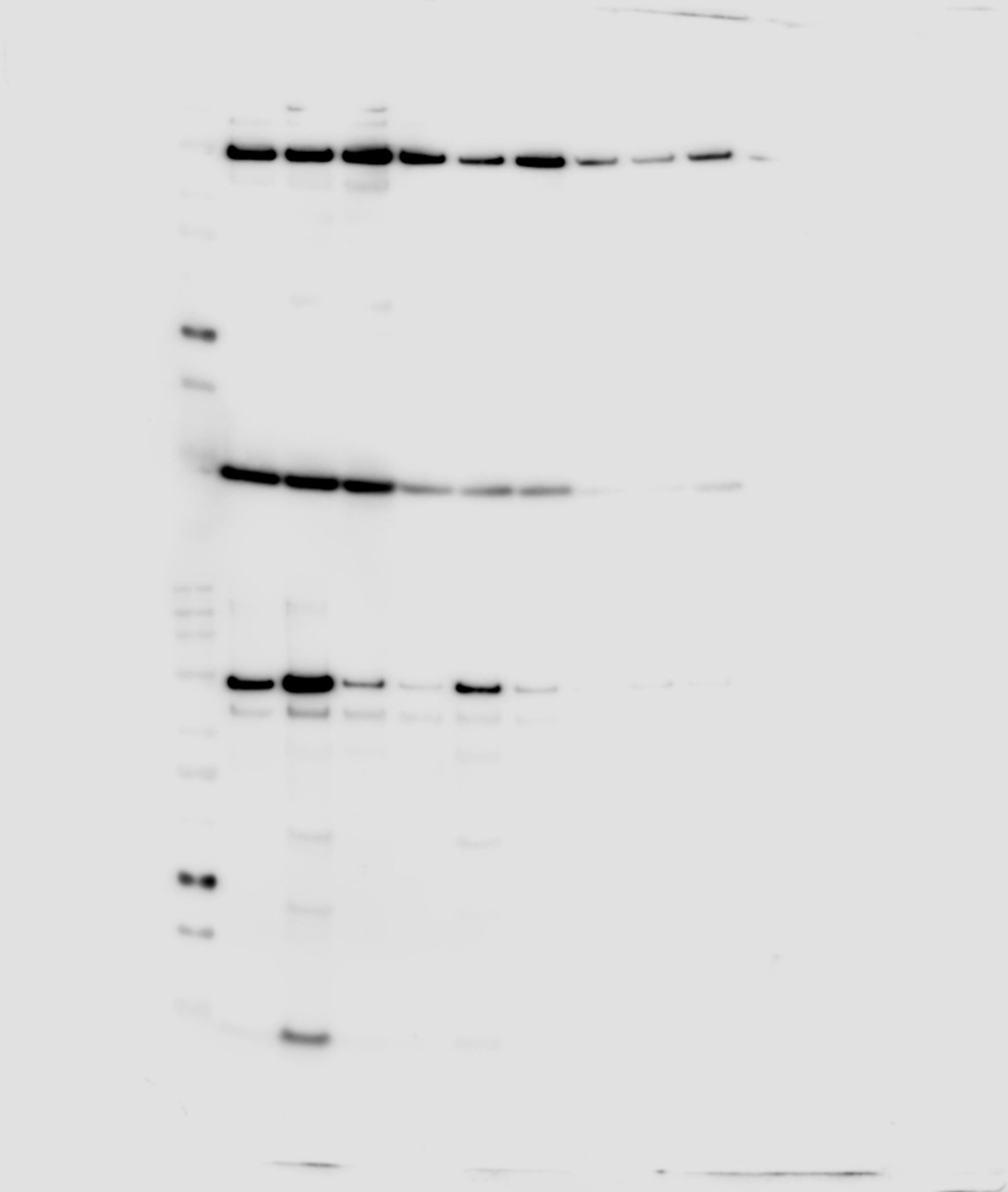

Supplement: Source data 2. [file elife-59999-data2.zip › Raw Unedited blots copy/Figure4G_inputpulldown_HA]

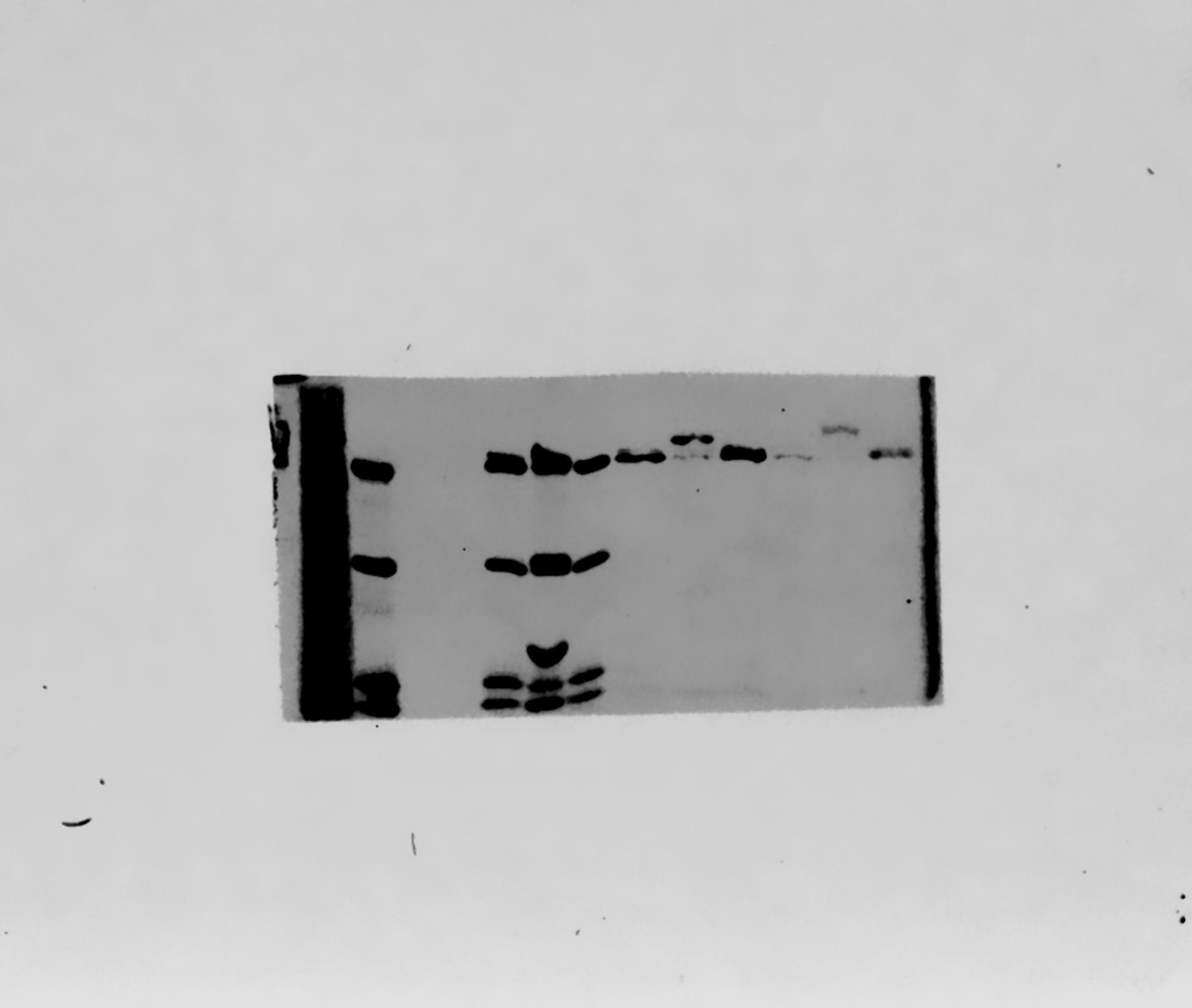

Supplement: Source data 2. [file elife-59999-data2.zip › Raw Unedited blots copy/Figure3C_PhostagGFP.tif]

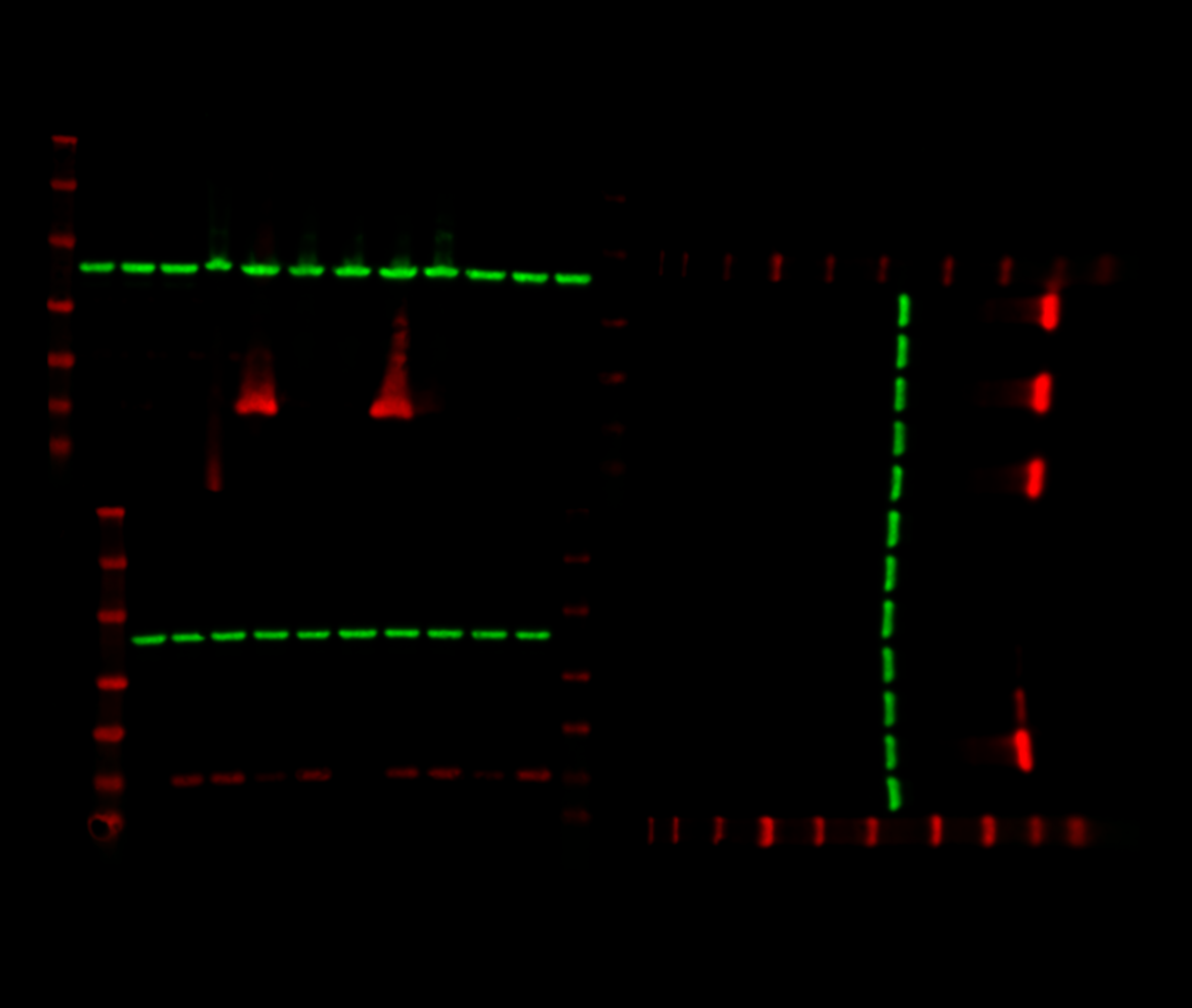

Supplement: Source data 2. [file elife-59999-data2.zip › Raw Unedited blots copy/Figure3_S1B_GAPDHpH3 GAPDHpH3.tif]

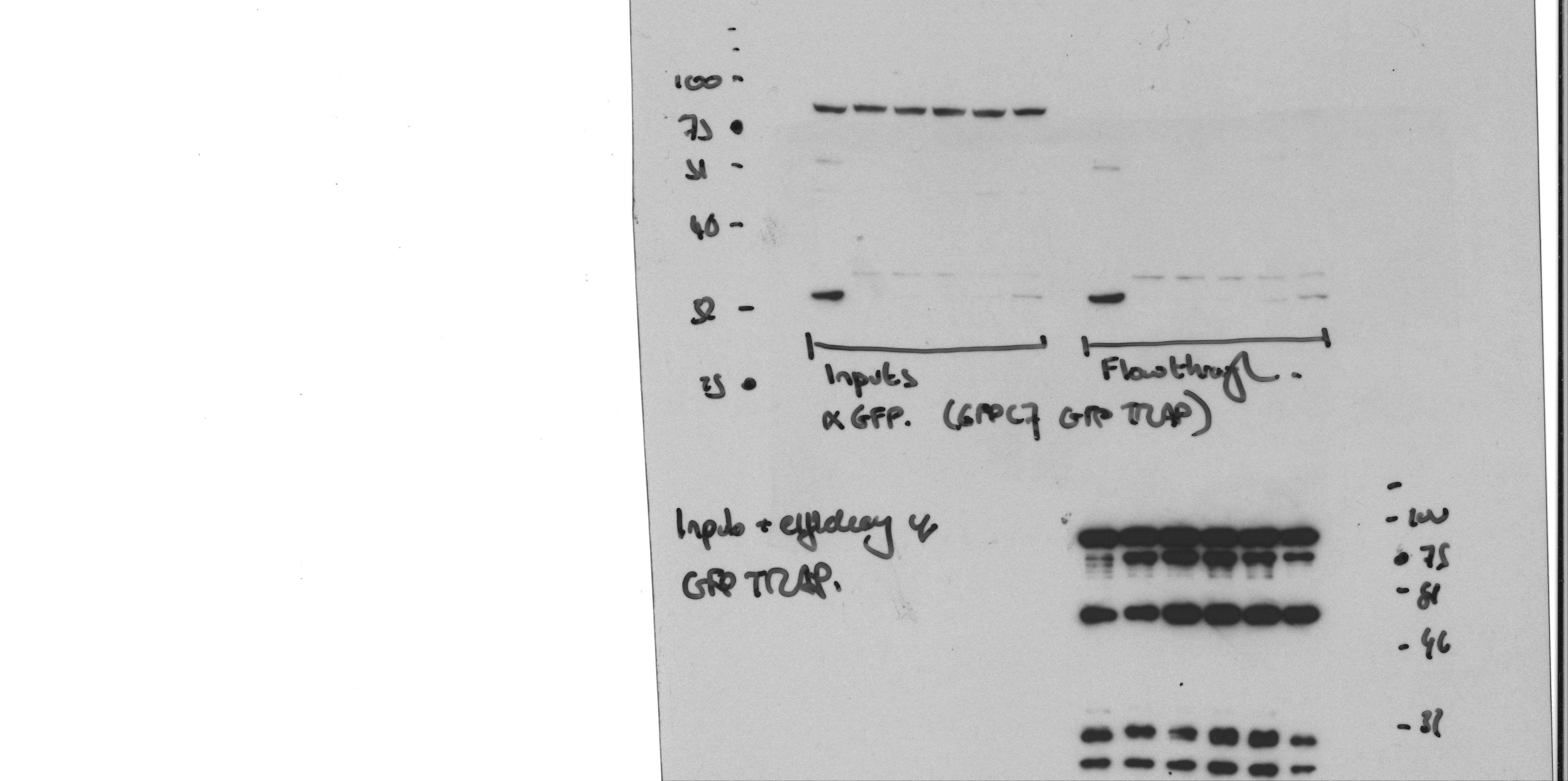

Supplement: Source data 2. [file elife-59999-data2.zip › Raw Unedited blots copy/Figure5E_GFP_innputs.jpg]

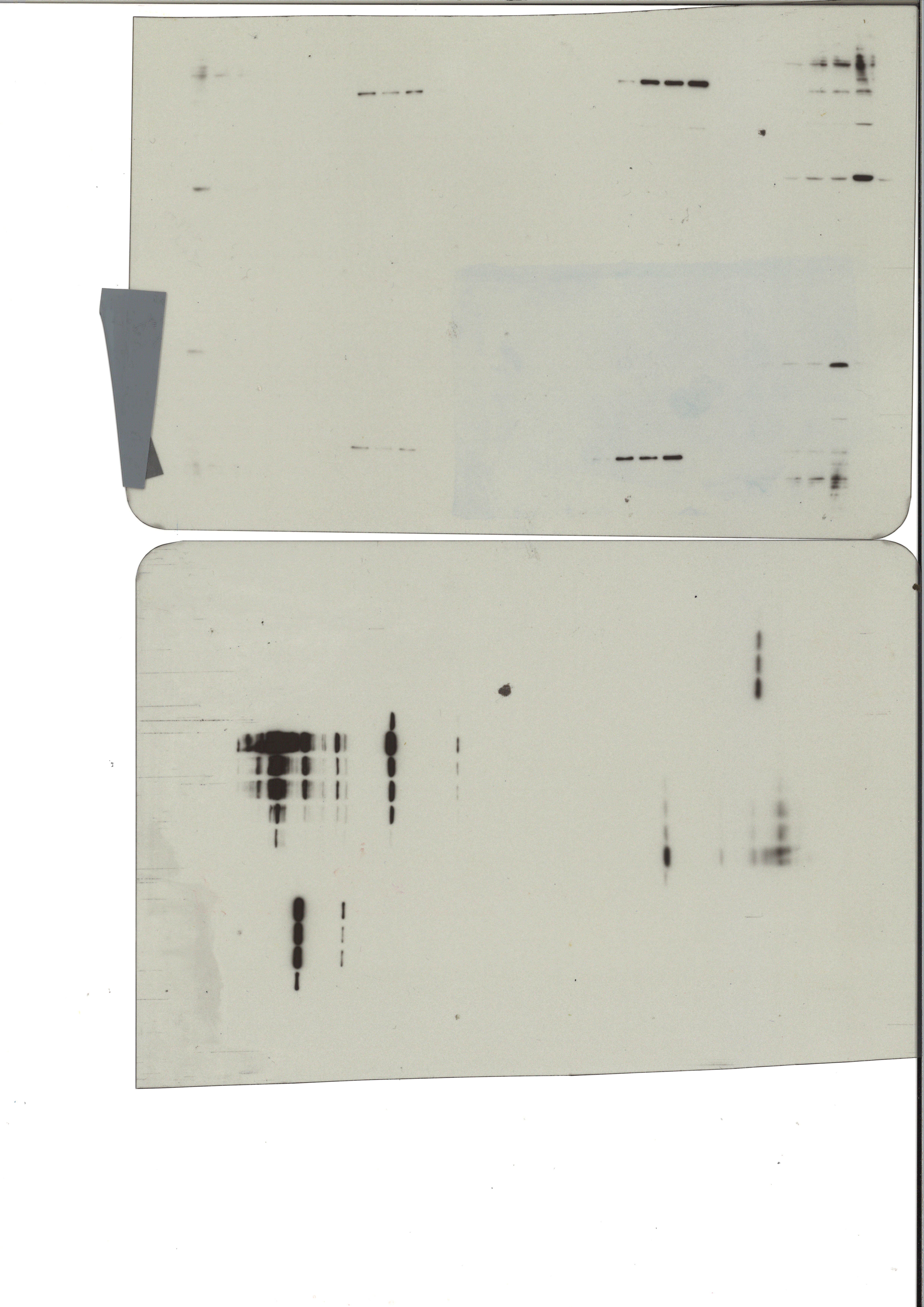

Supplement: Source data 2. [file elife-59999-data2.zip › Raw Unedited blots copy/FIgure5E_KH-pS-P_Pulldown.jpg]

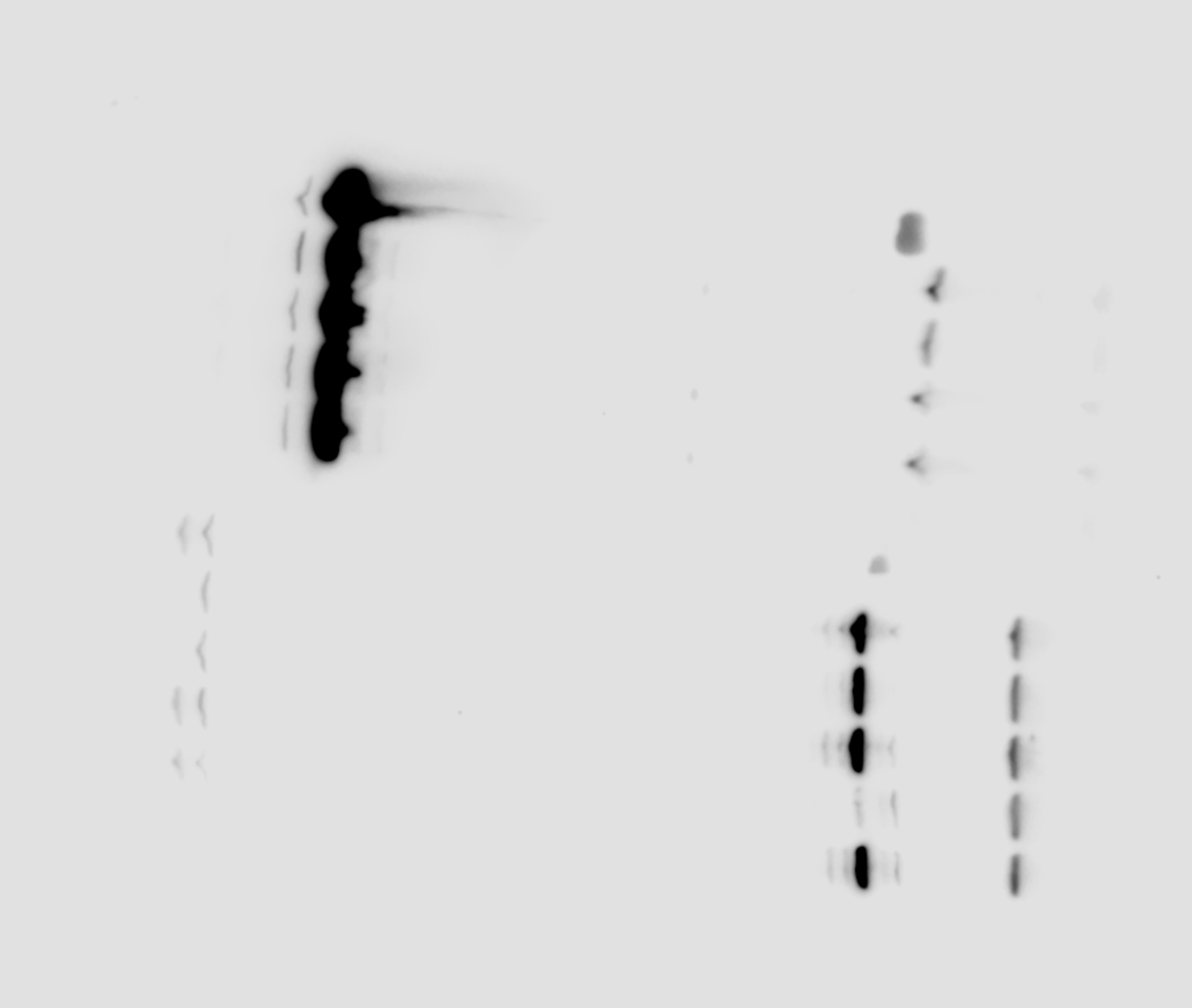

Supplement: Source data 2. [file elife-59999-data2.zip › Raw Unedited blots copy/Figure1B_IST1.tif]

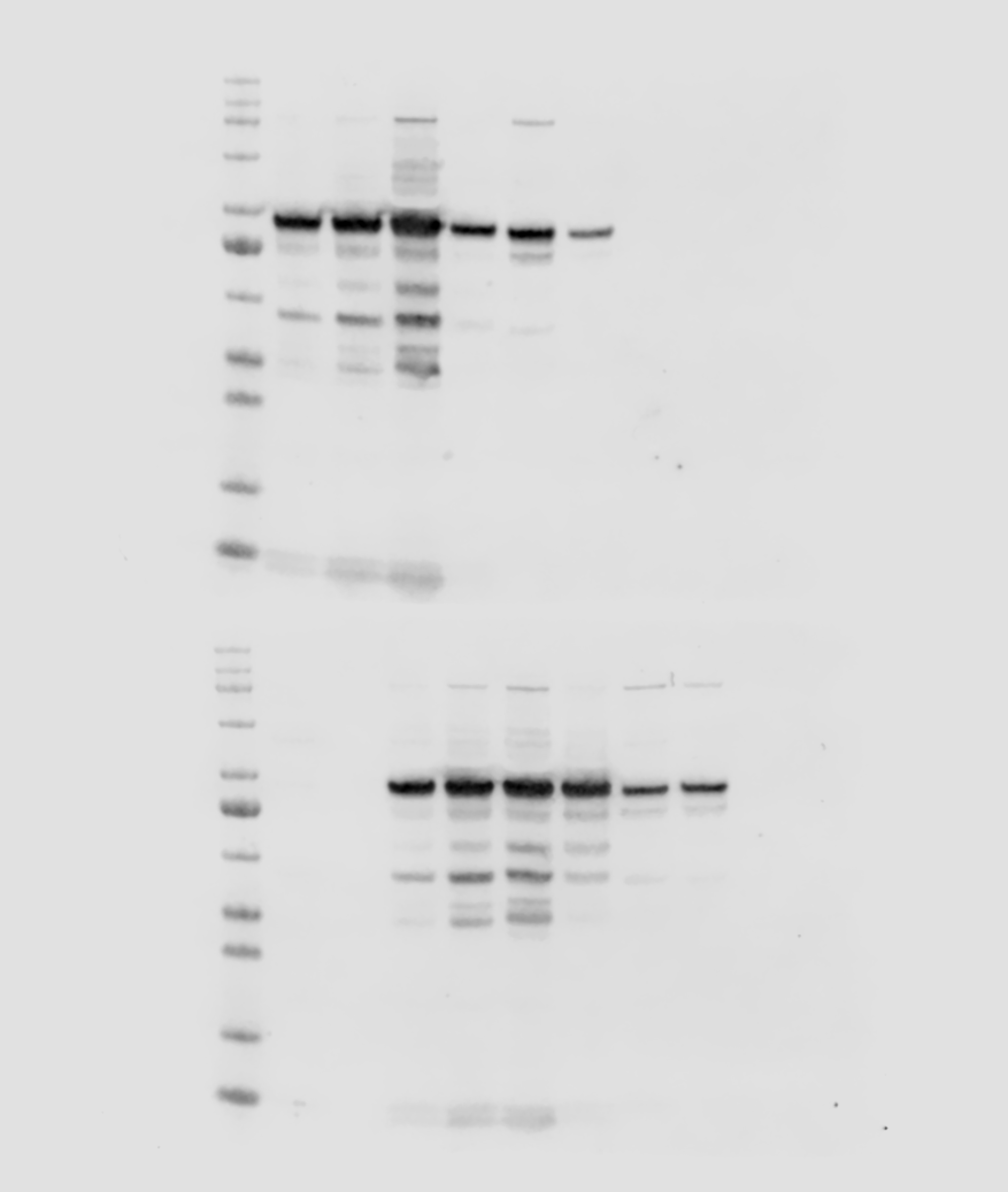

Supplement: Source data 2. [file elife-59999-data2.zip › Raw Unedited blots copy/Figure4_S1A_TopiswithoutLEM2CT_bottomiswithLEM2CT_CHMP7.tif]

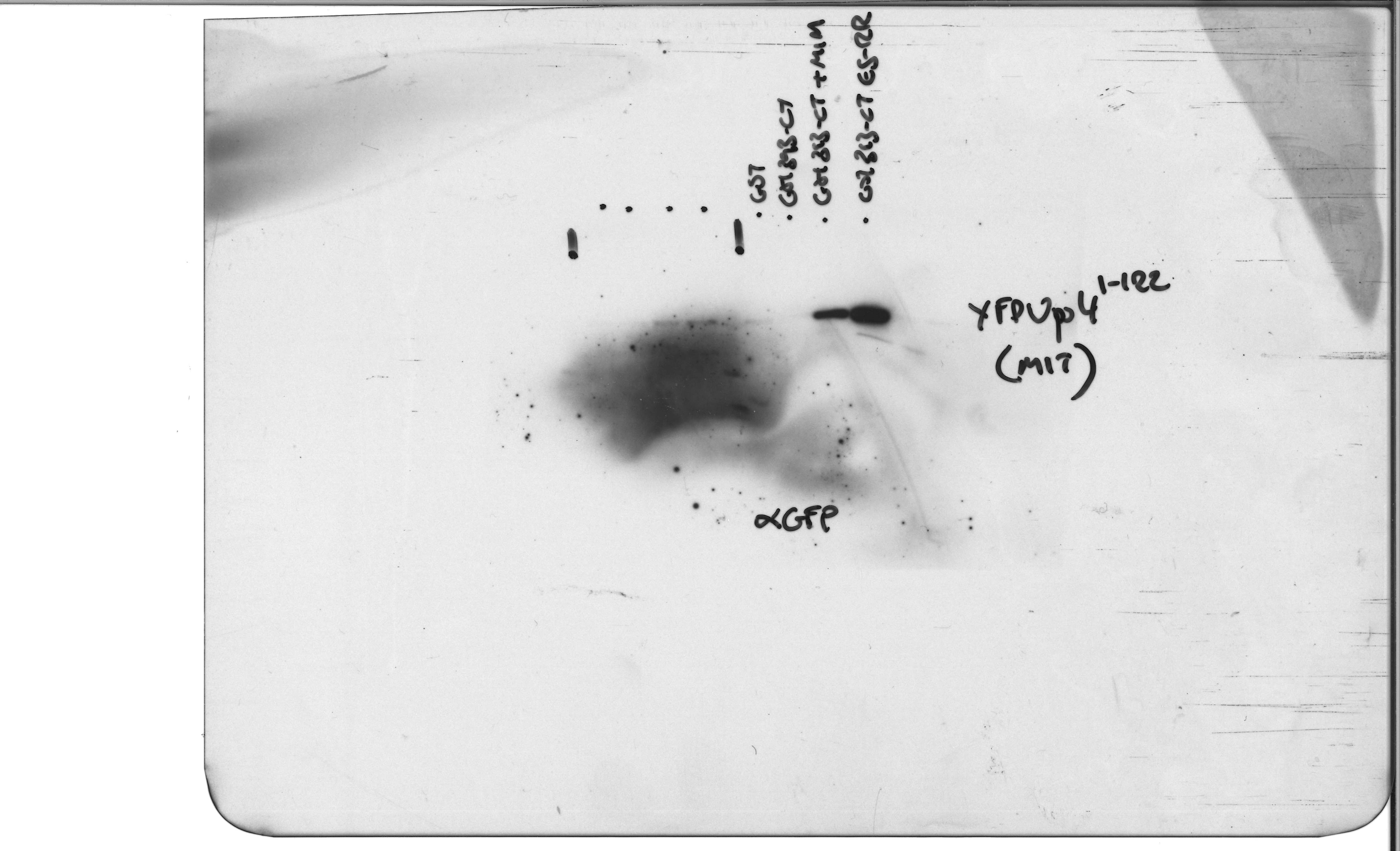

Supplement: Source data 2. [file elife-59999-data2.zip › Raw Unedited blots copy/Figure2_S2B_Pulldown_GFP.jpg]

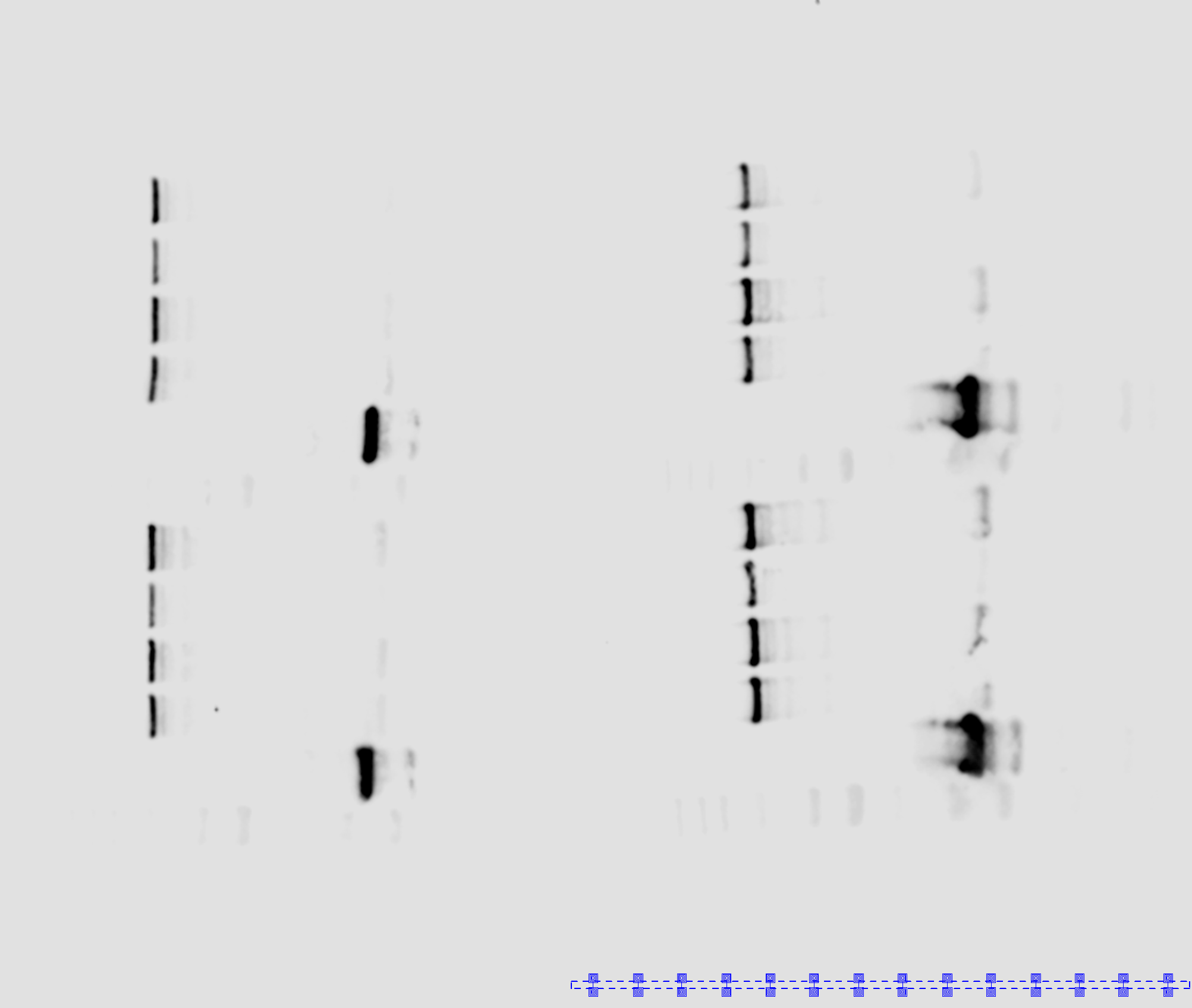

Supplement: Source data 2. [file elife-59999-data2.zip › Raw Unedited blots copy/Figure5_S2D_3892_IPtop_GTrapbottom_GFP.tif]

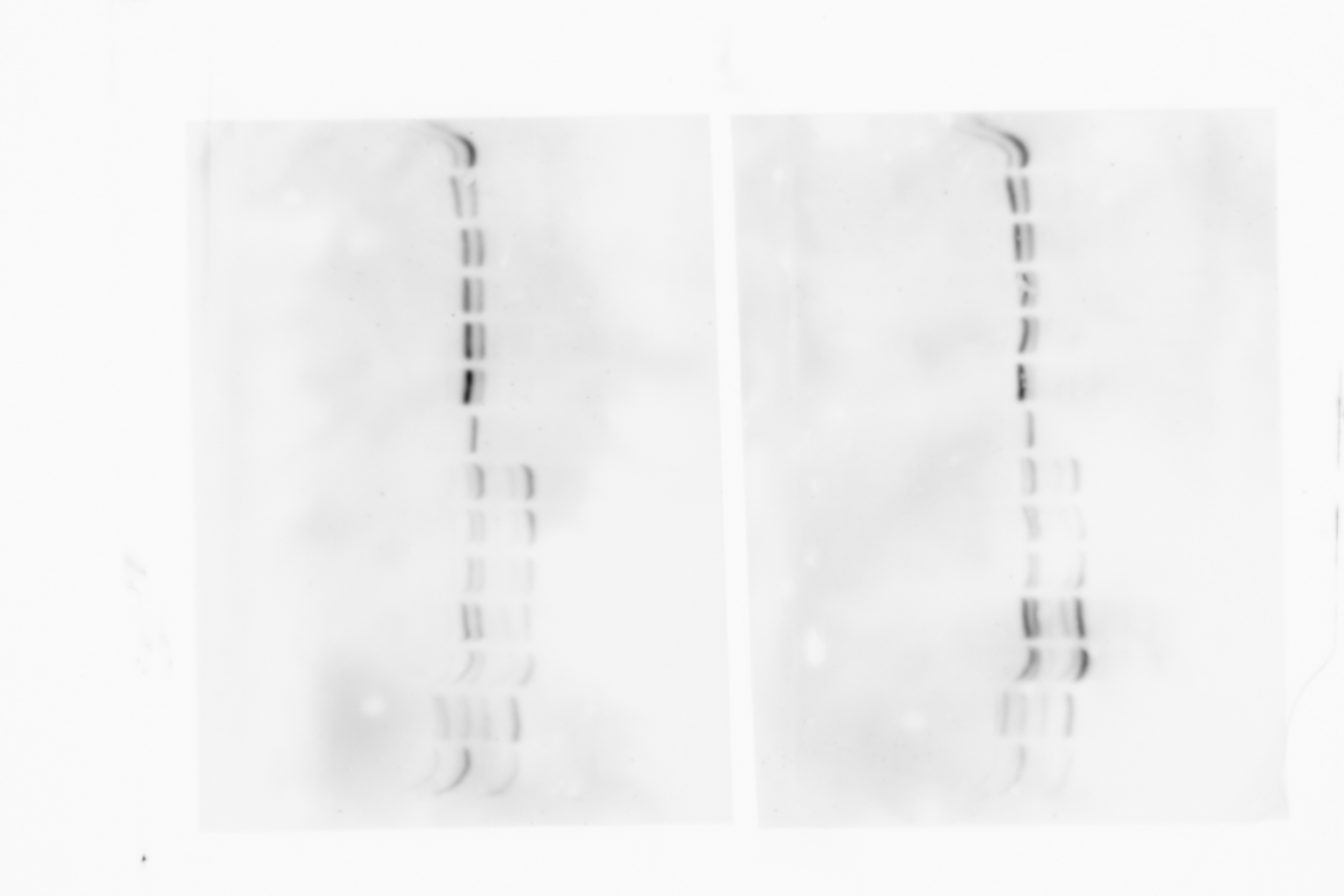

Supplement: Source data 2. [file elife-59999-data2.zip › Raw Unedited blots copy/Figure5_S2B_ERcytosol.tif]

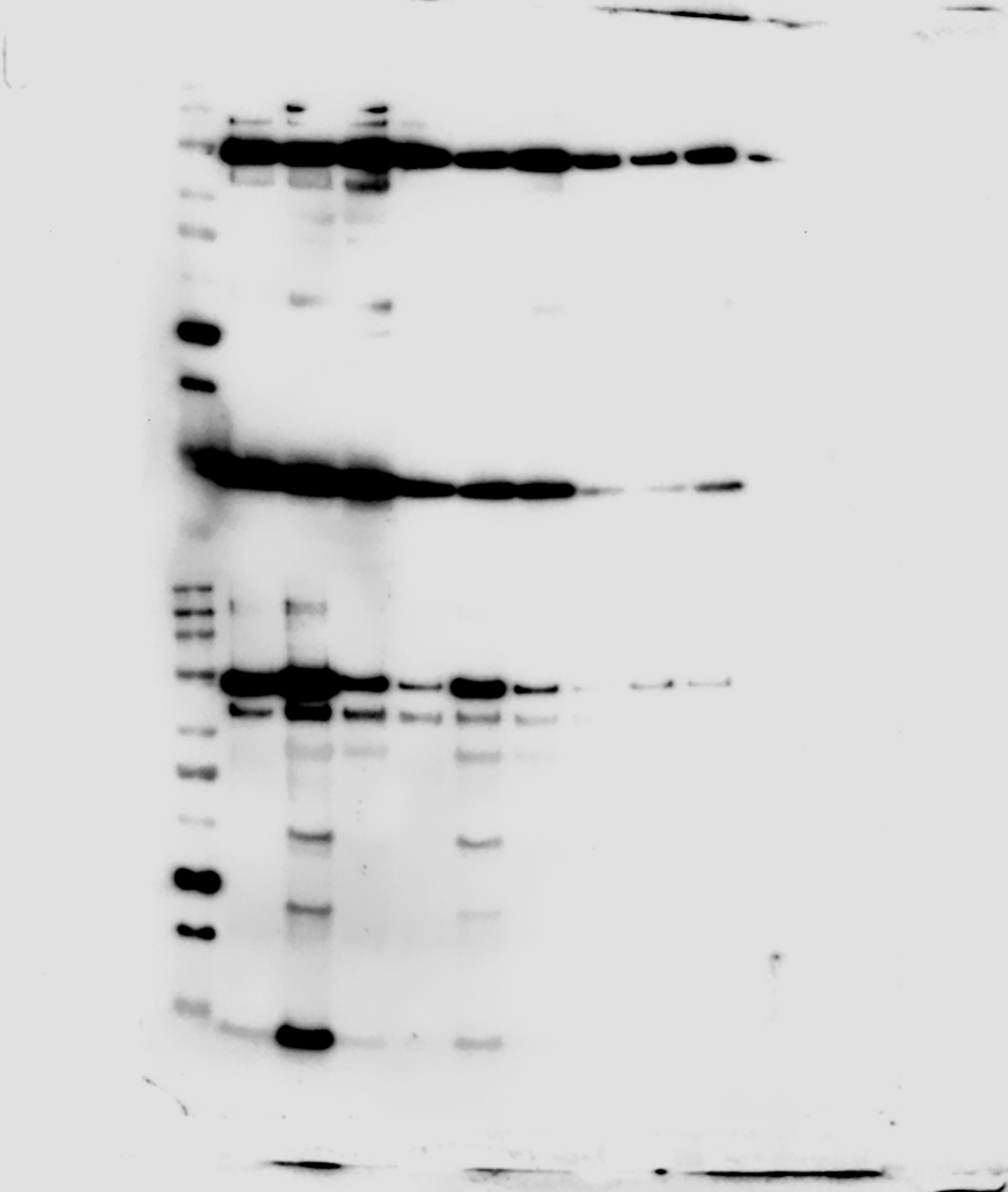

Supplement: Source data 2. [file elife-59999-data2.zip › Raw Unedited blots copy/Figure4G_inputpulldown_darkerexposure_HA]

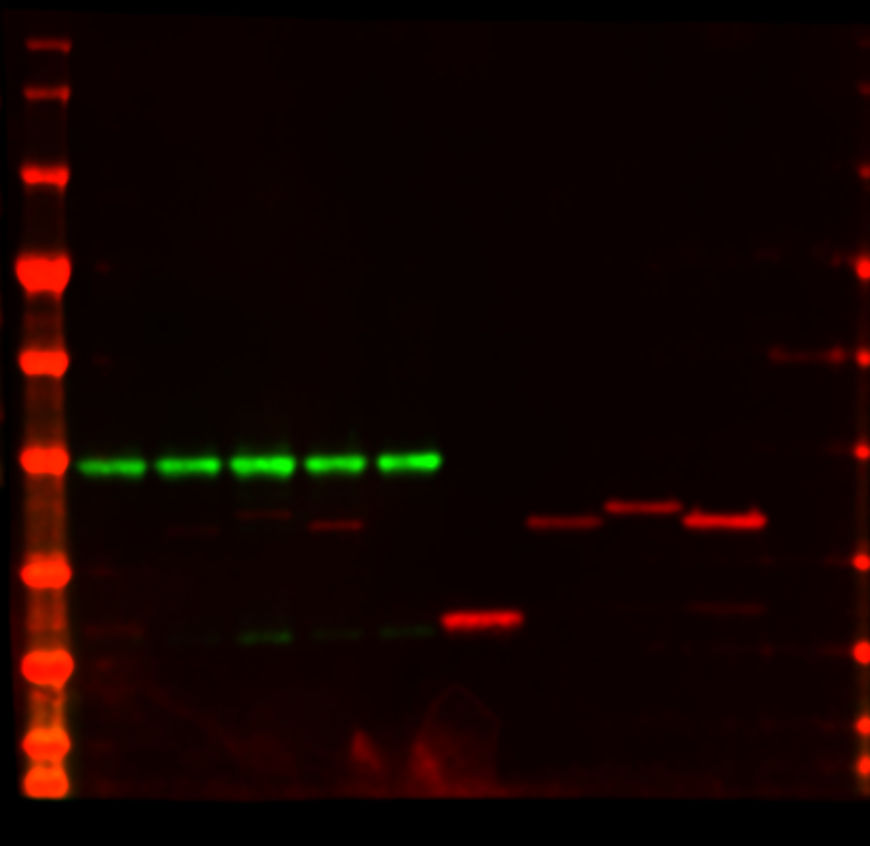

Supplement: Source data 2. [file elife-59999-data2.zip › Raw Unedited blots copy/Figure2_S2B_Input_GFPgreen_Pulldown_GSTred_copy.tif]

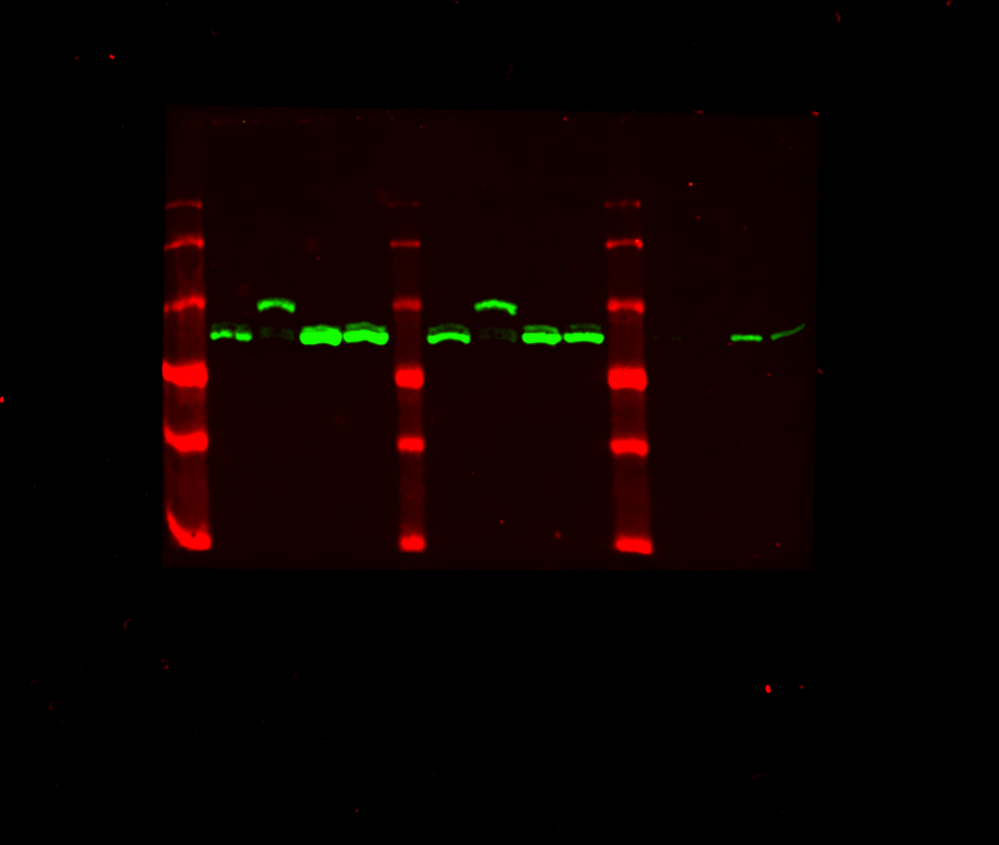

Supplement: Source data 2. [file elife-59999-data2.zip › Raw Unedited blots copy/Figure3D_GFP_phostag.png]
